# TTLL12 counteracts BPOZ-2 to stabilize eEF1A1 and promote hepatocarcinogenesis

**DOI:** 10.1038/s41419-026-08694-1

**Published:** 2026-04-21

**Authors:** Kaiming Leng, Zhen Yang, Yao Liming, Nanfeng Meng, Yi Xu, Guanqun Liao, Mei Han, Guangjun Shi

**Affiliations:** 1 Department of Hepatopancreatobiliary Surgery, Qingdao Municipal Hospital, Qingdao University, Qingdao, PR China; 2 Qingdao Hospital, University of Health and Rehabilitation Sciences (Qingdao Municipal Hospital), Qingdao, China; 3 Department of Colorectal Surgery, Shandong Cancer Hospital and Institute, Shandong First Medical University and Shandong Academy of Medical Sciences, Jinan, China; 4 Department of Colorectal Oncological Surgery, Cancer Center, The Second Affiliated Hospital of Harbin Medical University, Harbin, China; 5 Department of Hepatopancreatobiliary Surgery, The Second Affiliated Hospital of Harbin Medical University, Harbin, China; 6 Anhui Province Key Laboratory of Translational Cancer Research, Bengbu Medical College, Bengbu, China; 7 State Key Laboratory of Oncology in South China, Cancer Center of Sun Yat-Sen University, Guangzhou, China; 8 Shanghai Key Laboratory of Molecular Imaging, Shanghai University of Medicine and Health Sciences, Shanghai, China; 9 Department of Pathology, Li Ka Shing Faculty of Medicine, The University of Hong Kong, Hong Kong SAR, China; 10 Department of Hepatobiliary Surgery, Foshan Hospital Affiliated to Southern Medical University, Foshan, China; 11 Department of Pharmacology, School of Pharmacy, Qingdao University, Qingdao, China

**Keywords:** Mechanisms of disease, Gastrointestinal diseases

## Abstract

Tubulin tyrosine ligase-like enzyme-12 (TTLL-12), belonging to the TTLL family, holds a crucial position in posttranslational tubulin modifications implicated in cancer development, making it a potential target for anti-tumor therapy. However, the precise mechanisms of TTLL12 in HCC remain inadequately elucidated. In this study, we found markedly elevated expression of TTLL12 in HCC tissues compared to their adjacent normal counterparts, which is strongly linked to an unfavorable prognosis. Functional assays demonstrated that knockdown of TTLL12 significantly suppressed HCC cell proliferation, whereas overexpression of TTLL12 promoted oncogenic behaviors. In vivo models further confirmed that TTLL12 enhances tumor growth, as indicated by larger tumors in TTLL12-overexpressing cells compared to vector controls. Mechanistically, TTLL12 suppressed ubiquitin-proteasome-mediated degradation of eEF1A1 via competing with Bood POZ-containing gene type 2 (BPOZ-2), which typically recruits eEF1A1 to CULLIN (CUL3), and ultimately results in an enhancement of cancer cell proliferation. Our analyses underscore the oncogenic potential of TTLL12 in hepatocarcinogenesis by safeguarding eEF1A1 from BPOZ-2- mediated degradation, introducing a novel therapeutic target for combating HCC.

## Introduction

Liver cancer is a formidable global health challenge, ranking fourth in incidence and third in overall mortality among all cancers [1, 2]. Hepatocellular carcinoma (HCC), the predominant histological subtype, accounts for about 90% of all primary liver cancer. With high morbidity, mortality, and limited treatment efficacy, managing HCC is exceptionally challenging in clinical practice. Despite advancements in surgery, targeted treatments, and emerging immunotherapies, the 5-year overall survival for HCC patients remains disappointingly unfavorable, emphasizing the urgent need for novel tumor biomarkers to enhance early detection and molecular-targeted therapeutic strategies aimed at improving survival in individuals with HCC [3, 4].

Elevated polyglutamylation and dysregulated tyrosination/detyrosination of tubulin significantly influence microtubule function and behavior, and are increasingly being recognized as important regulators to cancer development [5, 6]. Polyglutamylation, catalyzed by tubulin tyrosine ligase-like enzymes (TTLL), has been established to be linked with tumorigenesis [7, 8]. Recent investigations into the TTLL family have shed light on cancer-related discoveries [9]. As a member of the TTLL family, TTLL12 functions as a prominent player in posttranslational tubulin modifications, making it a prospective target for anti-tumor therapy. However, the molecular mechanisms of TTLL12 in HCC remain inadequately characterized.

The highly conserved eukaryotic translation elongation factor 1A1 (eEF1A1) is traditionally recognized for its critical role in the elongation phase of protein translation in eukaryotic cells [10, 11]. However, emerging evidence indicates that eEF1A1 exerts regulatory effects beyond its canonical role in translation by modulating multiple cellular processes, including cell cycle progression, cytoskeletal organization, and signal transduction [12]. Specifically, eEF1A1 interacts with key regulators of the cell cycle, such as cyclins and cyclin-dependent kinases, which facilitate the orderly transition through different cell cycle phases. Additionally, eEF1A1’s non-canonical functions include its capacity to bind to actin filaments, thereby influencing cytoskeletal dynamics and impacting cellular processes such as migration, adhesion, and division, all of which are tightly linked to cancer cell proliferation [13, 14]. Furthermore, eEF1A1 is involved in major signaling pathways, including the PI3K/AKT and MAPK pathways, which are critical for promoting cell survival, growth, and proliferation [15, 16]. Dysregulation of eEF1A1 expression, characterized by its aberrant overexpression, has been observed in various malignancies, including HCC, where it is closely associated with increased tumor cell proliferation and poor prognosis [17, 18]. Conversely, suppression of eEF1A1 expression has been shown to reduce cell proliferation, highlighting its role as a potential therapeutic target. Therefore, further elucidation of eEF1A1’s multifaceted functions in cellular homeostasis and tumorigenesis could provide crucial insights for the development of targeted therapeutic strategies in HCC.

In this study, we investigated mechanisms underlying eEF1A1 overexpression in HCC and found that TTLL12 regulates eEF1A1 expression by competing with BPOZ-2 and inhibiting BPOZ-2-mediated eEF1A1 ubiquitination and degradation. These findings establish the rationale for considering TTLL12 as a potential drug target for HCC.

## Materials and methods

### Cell lines and reagents

Human embryonic kidney 293 (HEK293) cells, along with human liver cells MIHA, THLE-2, and HCC cell lines Huh7, Hep3B, MHCC97H, MHCCLM3, were maintained in Dulbecco’s Modified Eagle’s Medium containing 10% fetal bovine serum. Regular mycoplasma contamination tests were conducted on all cell lines, ensuring their negative status.

Western blotting analyses were conducted utilizing a panel of primary antibodies targeting specific proteins: TTLL12 (Proteintech, Cat No. 84493-6-RR), eEF1A1 (Abcam, Cat No. ab157455), BPOZ-2, β-catenin (Abcam, Cat No. ab32572), Flag (Abcam, Cat No. ab205606), human hemagglutinin, ubiquitin (Abcam, Cat No. ab140601), His (Abcam, Cat No. ab18184), and GAPDH (Proteintech, Cat No. 60004-1-Ig).

### Clinical sample collection

Human samples included two cohorts: (1) tissue microarrays commercially obtained from Shanghai Xinchao Biotechnology Co., Ltd., which were ethically approved by the company’s committee (No. SHYJS-CP-1804007) and used without additional informed consent due to full anonymization; (2) fresh clinical tissues collected from Qingdao Municipal Hospital with written informed consent from all participants and approval from the hospital’s ethics committee. Inclusion criteria for the latter cohort required primary HCC diagnosis, no prior chemotherapy/radiotherapy, and complete clinical records, while exclusion criteria excluded cases with concurrent extrahepatic malignancies, insufficient tissue quantity, or incomplete follow-up data. Tissues were immediately snap-frozen in liquid nitrogen and stored at −80 °C to preserve integrity.

### Plasmid construction and mutagenesis

All plasmid construction and source information are as follows: The mammalian expression vectors for Flag-TTLL12, HA-TTLL12, and Flag-BPOZ2 were constructed by cloning the full-length coding sequences of human TTLL12 or BPOZ-2 into the pcDNA3.1 vector with an N-terminal Flag or HA tag. The HA-eEF1A1 and His-eEF1A1 plasmids were generated by cloning the eEF1A1 gene into a mammalian expression vector with an HA tag and the prokaryotic expression vector pET-28a (with a 6xHis tag), respectively. The His-Ub and HA-Ub plasmids were generated by cloning the human ubiquitin coding sequence into pET-28a and pCMV-HA vectors, respectively. The GST control plasmid was the empty pGEX-4T-1 vector. In addition to the plasmids constructed as described above, some plasmids were gifts from Harbin Medical University. All point mutant constructs were generated using a site-directed mutagenesis kit and verified by Sanger sequencing. shRNA expressing plasmids were constructed in a pLKO.1 vector.

### RT-qPCR

Total RNA extraction was carried out using TRIzolTM (Sigma, USA), and subsequent cDNA synthesis was performed using a reverse transcription kit (Roche, Germany) in accordance with the manufacturer’s protocols. Quantitative PCR was conducted employing the ABI 7500 instrument and SYBR Green (Roche, Germany). The sequences of eEF1A1 and GAPDH primers were shown in Supplementary Table S1.

### Colony formation assay

Cells were resuspended in culture medium, and an appropriate number of cells from both the control and treatment groups were seeded into a 6-well plate. Following a 2-week incubation period at 37 °C with 5% CO_2_, the resultant colonies underwent fixation with methanol and acetic acid and subsequent staining with crystal violet. The quantification process involved determining the number of viable colonies, specifically defined as those surpassing a threshold of 50 cells.

### Immunofluorescence

Cell slides were initially fixed with 4% paraformaldehyde and permeabilized with Triton X-100. Subsequent steps involved incubation with specific primary antibodies, followed by fluorescence-labeled secondary antibodies and staining with DAPI to label the nuclei. Images were captured using a confocal microscope, and the analysis was conducted using ImageJ.

### Immunohistochemistry (IHC)

Human tumor samples, accompanied by clinical details from patients diagnosed with HCC, were collected for our study. Both human and animal tumor tissues were fixed by formalin and embedded into paraffin. Subsequently, the tumor tissue sections underwent incubation with specific primary antibodies. The staining index score for samples was calculated by combining the intensity of the immune reaction with the percentage values.

### Construction of TTLL12 expression vectors

Utilizing the GenBank information associated with TTLL12 (NM_015140), the nucleotide sequences of three short hairpin RNAs (sh-TTLL12-1, sh-TTLL12-2, and sh-TTLL12-3) targeting TTLL12, as well as the negative control short hairpin RNA (sh-NC), have been delineated in Supplementary Table S1. Lentiviral vectors containing sh-TTLL12 and sh-NC were meticulously engineered using the GV493 vector sourced from Genechem Inc., headquartered in Shanghai, China. The lentiviral infection procedure adhered strictly to the guidelines stipulated by the manufacturer. Subsequent to this, the selection of singular-cell clonal isolates was executed through exposure to puromycin over a period spanning 2–4 weeks.

### Immunoblotting and co-immunoprecipitation assay

Whole cell extracts were prepared with lysis buffer, supplemented with protease inhibitor cocktail and phosphatase inhibitor. The solubilized proteins were then separated through SDS-PAGE and subjected to Western blot analysis. For co-IP, whole cell extracts were gently suspended in 1 mL of cell lysis buffer, which included a 1% protease inhibitor cocktail, and kept at 4 °C for 20 min. Anti-Myc, anti-Flag, or anti-HA immunomagnetic beads were then introduced into the solubilized proteins and allowed to incubate overnight at 4 °C. Then the immunomagnetic beads were retrieved using a magnet and eluted, before proceeding with the subsequent western blotting.

### Ethynyldeoxyuridine (EdU) staining

The EdU incorporation assay was conducted according to the manufacturer’s instructions to evaluate the ability of cell proliferation. Culture medium containing EdU was used for cell incubation to facilitate DNA labeling. Then, the cells underwent fixation and permeabilization. Subsequently, a reaction mixture incorporating a fluorescent dye was introduced, and the plates were subjected to dark incubation.

### RNA sequencing

RNA sequencing was performed to investigate the transcriptional changes induced by TTLL12 overexpression. Huh7 cells were stably transfected with either the Flag-TTLL12 plasmid (TTLL12 overexpression group) or the empty pcDNA3.1 vector (Control group), with three independent biological replicates prepared for each group. Total RNA was extracted using TRIzol reagent. Following quality control, sequencing libraries were constructed using the NEBNext Ultra II Directional RNA Library Prep Kit and subjected to sequencing on an Illumina platform. Differential expression analysis was performed with DESeq2, with genes exhibiting an adjusted *p*-value < 0.05 defined as differentially expressed genes (DEGs). The expression profile of significant DEGs was analyzed by hierarchical clustering to generate the heatmap.

### Animal experiments

Animal experiments were conducted in accordance with the NIH Guide for the Care and Use of Laboratory Animals and approved by the Animal Ethics Committee of Shandong University (No. SYDWLL-2024-169). To initiate the experiment, 1 × 10^6^ HCC cells were suspended in PBS for the subcutaneously injection. Tumor volume was calculated every 3 days for 18 days (6 timepoints), and was measured using the formula: 0.5 × (*L* × *W*^2^)(“*L”*, the length; “*W*“, the width). Upon completion of the measurement, when the nude mice were euthanized, tumor tissues were harvested. For the implantation of tumors into the liver, tumor seeds were meticulously sectioned into approximately 1 mm^3^ cubes and introduced into the livers of 6-week-old mice. Under anesthesia, the mice’s livers were surgically exposed and subjected to controlled mechanical injury using a surgical needle. The implanted tumor cubes were then secured within the incision using sutures. Post-implantation, we systematically monitored hepatic tumor growth on a weekly basis for a duration of 6 weeks employing bioluminescent imaging. This involved the administration of D-luciferin (Xenogen, Hopkinton, MA, USA) to the anesthetized animals to facilitate bioluminescent signal acquisition. The resultant images were meticulously captured and analyzed using the IVIS 100 Imaging System (Xenogen). Upon the culmination of the experimental timeline, the assessment of potential metastatic occurrences necessitated the removal of lungs from the animals before subjecting them to bioluminescent imaging. Animals were allocated to the experimental groups without randomization. Investigators were not blinded to group allocation during the experiments or outcome assessment.

### Statistical analysis

The GraphPad Prism 9 statistical software was used for statistical analysis. The data are expressed as average ± SD. ANOVA was used to evaluate the differences among groups, and *p* < 0.05 was considered to be statistically significant.

## Results

### TTLL12 regulates eEF1A1 expression and promotes HCC cell proliferation

**Fig. 1:**
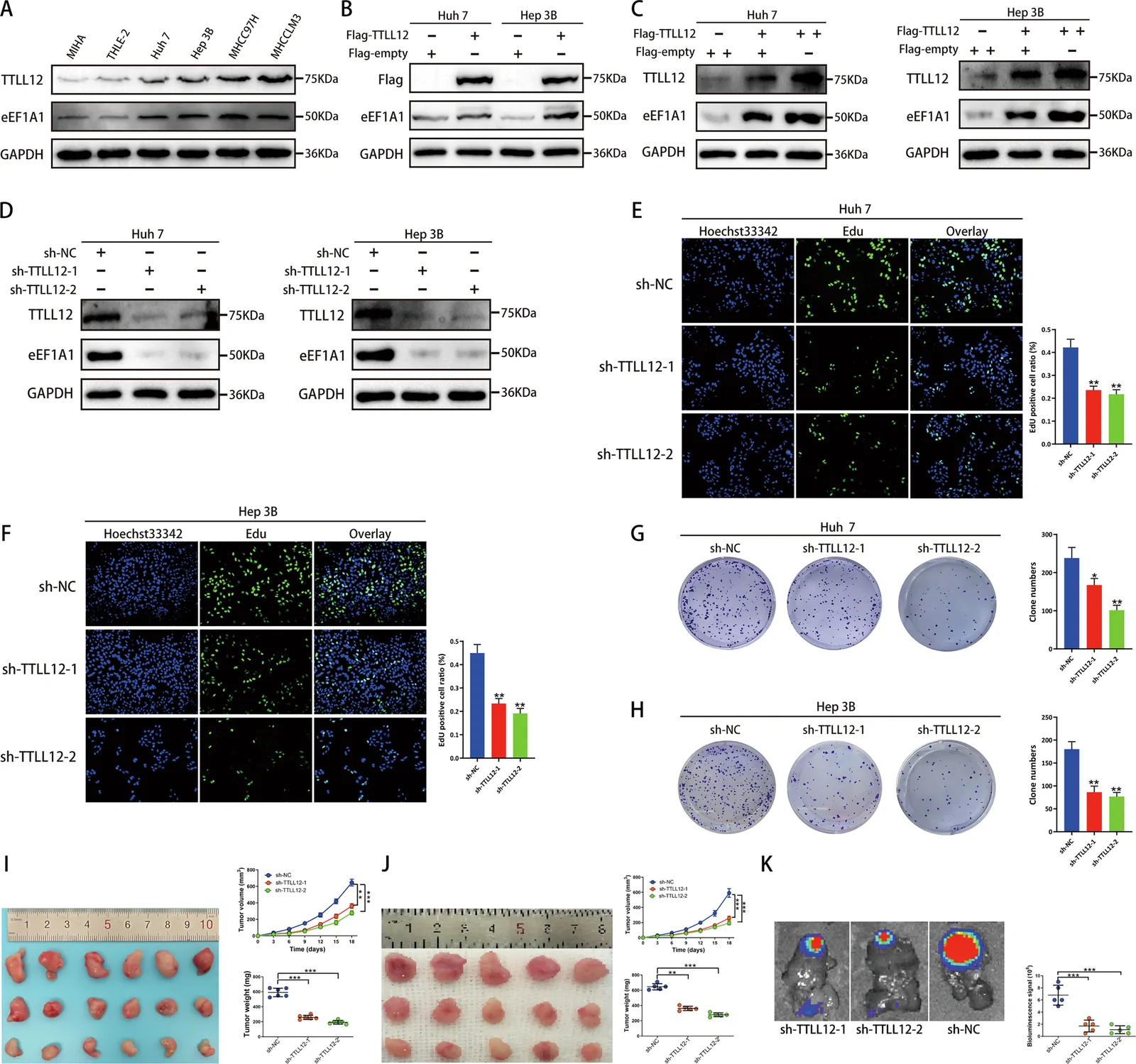
TTLL12 regulates eEF1A1 expression and promotes HCC cell proliferation. **A** Western blot analysis depicting the expression levels of TTLL12 and eEF1A1 in normal human liver cells and HCC cell lines. **B**–**D** Evaluation of eEF1A1 protein levels through western blot analysis in Huh7 and Hep3B cells subjected to increasing amounts of TTLL12 plasmid or knockdown of TTLL12. **E**–**H** Assessment of cancer cell proliferation capacity using EdU and colony formation assays in Huh7 and Hep3B cells transfected with shTTLL12 plasmid. **I**–**K** Demonstration of the inhibitory effect of stable TTLL12 knockdown on tumorigenicity in both nude mice and orthotopic liver implantation models.

Utilizing the STRING protein-protein interaction database [functional protein association networks (https://www.string-db.org/)], we identified eEF1A1 as a candidate interaction partner of TTLL12 (Supplementary Fig. S1A). The mass spectrometry results further confirmed eEF1A1 as one of the strongest interactors of TTLL12, consistent with the STRING predictions (Supplementary Fig. S1B and Table S2). To investigate the regulatory role of TTLL12 in eEF1A1 expression within HCC cells, we conducted western blot analysis. The results showed that both TTLL12 and eEF1A1 protein levels were elevated in HCC cells compared to normal liver cells (Fig. 1A). Moreover, overexpression of TTLL12 in Huh7 and Hep3B cells led to a dose- dependent increase in endogenous eEF1A1 levels (Fig. 1B, C), whereas knockdown of TTLL12 significantly reduced eEF1A1 expression at the protein level (Fig. 1D), with no corresponding changes in mRNA abundance (Supplementary Fig. S2). Colony formation and EdU proliferation assays revealed that TTLL12 knockdown significantly impaired HCC cell proliferation (Fig. 1E–H). To explore the in vivo role of TTLL12, we transplanted Huh7 and Hep3B cells with either control or TTLL12 knockdown lentiviral vectors into nude mice. Knockdown of TTLL12 was associated with a substantial inhibition of tumor growth (Fig. 1I, J and Supplementary Fig. S3). Compared to the control, the TTLL12 knockdown group exhibited significantly smaller tumors in an orthotopic liver implantation model (Fig. 1K). These findings underscore the regulatory role of TTLL12 in modulating eEF1A1 expression and promoting cell proliferation.

### eEF1A1 is required for TTLL12-mediated HCC cell proliferation

**Fig. 2:**
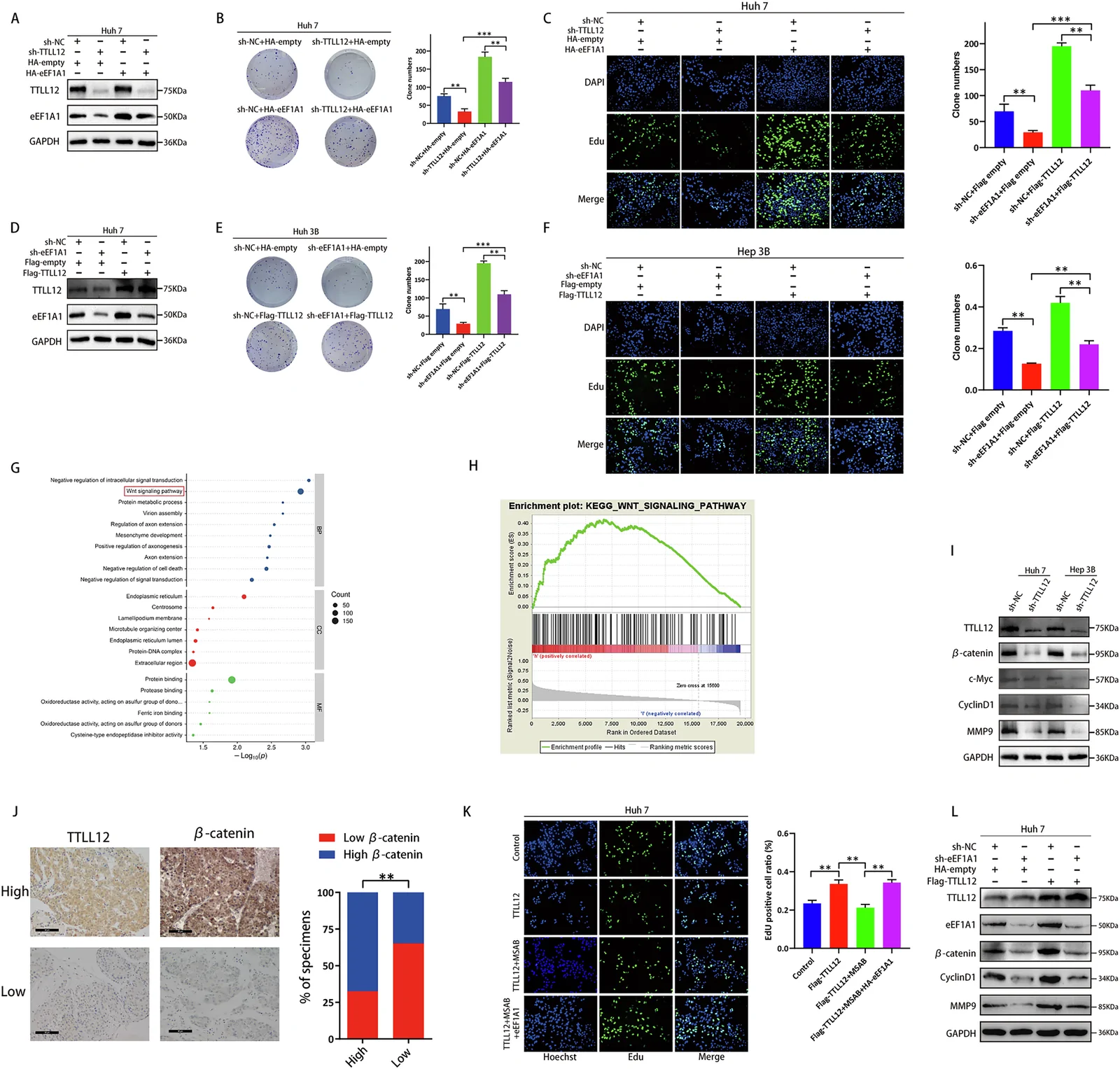
eEF1A1 is required for TTLL12-mediated HCC cell proliferation. **A**–**C** Huh7- shTTLL12 cells transfected with HA-eEF1A1 were subjected to western blot and EdU assays analyses for indicated proteins and cell proliferation capability. **D**–**F** Hep3B- sheEF1A1 cells transfected with Flag-TTLL12 were subjected to western blot and EdU assays analyses for indicated proteins and cell proliferation capability. **G** Bubble diagram illustrating enriched pathways in HCC tissues. **H** Gene Set Enrichment Analysis (GSEA) based on data from The Cancer Genome Atlas (TCGA). **I** Detection of protein levels associated with the Wnt signaling pathway via western blot in Huh7 and Hep3B cells treated with shNC or shTTLL12. **J**, Immunohistochemical (IHC) staining depicting TTLL12 and β-catenin expression. **K** Assessment of cell proliferation capacity through EdU assay in cells transfected with TTLL12 plasmid and treated with MSAB (Wnt signaling pathway inhibitor). **L** Impact of eEF1A1 knockdown on TTLL12-mediated expression of Wnt signaling-related proteins in Huh7 cells via western blot.

To further elucidate the pivotal role of TTLL12 in orchestrating HCC cell proliferation through the regulation of eEF1A1, we first elevated eEF1A1 expression in TTLL12 knockdown cells and assessed both TTLL12 and eEF1A1 expression levels, as well as cell proliferation. Our findings demonstrated that TTLL12 knockdown led to a decrease in eEF1A1 expression, whereas restoring eEF1A1 expression significantly reversed the reduction seen in TTLL12 knockdown Huh7 cells (Fig. 2A). Moreover, TTLL12 knockdown markedly inhibited cell proliferation, while enhanced eEF1A1 expression rescued the reduced proliferative ability caused by TTLL12 knockdown (Fig. 2B, C). In parallel, we attenuated eEF1A1 expression in TTLL12-overexpressing Hep3B cells and evaluated the corresponding levels of TTLL12 and eEF1A1, alongside cancer cell proliferation. The results showed that TTLL12 overexpression led to a significant increase in eEF1A1 expression, and eEF1A1 suppression effectively counteracted the TTLL12-induced elevation in eEF1A1 (Fig. 2D). Additionally, downregulation of eEF1A1 substantially diminished TTLL12-enhanced cell proliferation (Fig. 2E, F).

To gain a deeper insight into TTLL12’s role in HCC progression, mRNA sequencing was conducted, revealing genes with altered expression enriched in specific signaling pathways (Supplementary Fig. S4). Notably, TTLL12 overexpression was associated with the upregulation of genes linked to the Wnt signaling pathway (Fig. 2G), a finding consistent with data from the Cancer Genome Atlas (TCGA) (Fig. 2H). Western blot analysis further demonstrated that TTLL12 knockdown suppressed the expression of Wnt pathway-related genes (Fig. 2I). IHC staining revealed a positive correlation between TTLL12 and β-catenin protein expression (Fig. 2J). Additionally, inhibition of the Wnt signaling pathway via MSAB attenuated the oncogenic effects induced by TTLL12 (Fig. 2K). Western blot analysis also confirmed that eEF1A1 knockdown significantly reversed the TTLL12-mediated increase in β-catenin, Cyclin D1, and MMP9 expression in Huh7 cells (Fig. 2L). Collectively, these findings suggest that TTLL12 promotes HCC proliferation through modulation of the Wnt signaling pathway and highlight the critical role of eEF1A1 in TTLL12-driven cell proliferation.

### TTLL12 interacts with eEF1A1 in vitro

**Fig. 3:**
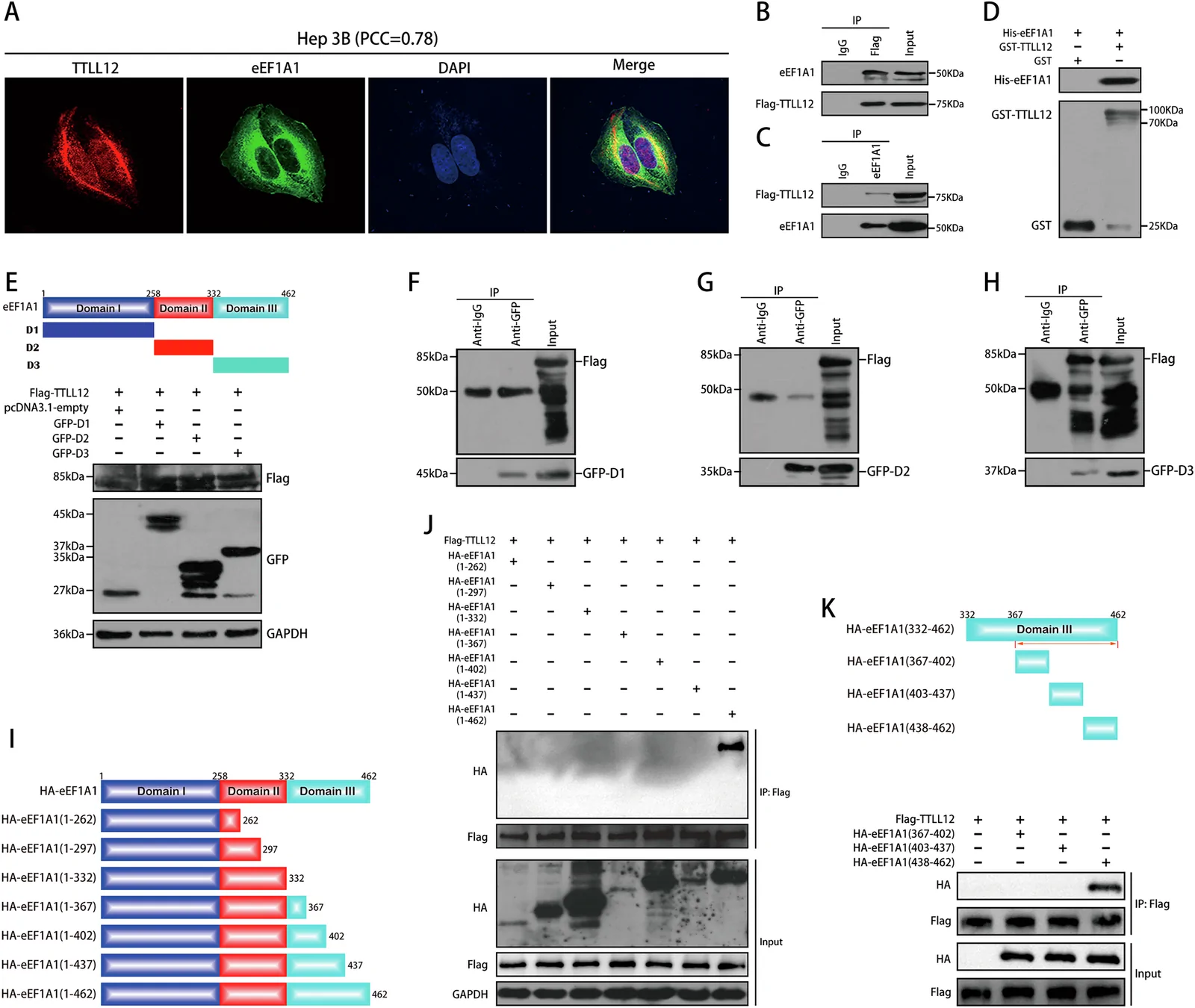
TTLL12 interacts with eEF1A1 in vitro. **A**–**C** Immunofluorescent staining and Co-IP analysis of TTLL12 and eEF1A1 in Hep3B cells. **D** GST pull-down experiments employing GST-TTLL12 protein and in vitro translated eEF1A1 protein. **E** Schematic representation of eEF1A1, comprising three functional domains. HEK293 cells co-transfected with HA-eEF1A1 domains 1, 2, and 3 and Flag-TTLL12. Cell lysates subjected to IP using anti-GFP beads to detect Flag binding. **F**–**H** Immunoblotting of lysates after IP from HEK293 cells transfected with indicated plasmids. **I**, Schematic representation of eEF1A1 (462aa) highlighting different mutants. **J**, **K** HEK293 cells transfected with indicated constructs. Subsequent cell lysis for Co-IP using anti-Flag beads to detect HA binding.

To elucidate the mechanisms by which TTLL12 modulates eEF1A1 expression, we investigated the interaction between TTLL12 and eEF1A1. Immunofluorescence assays revealed co-localization of TTLL12 with eEF1A1 in the cytoplasm of Hep3B cells (Fig. 3A). Co-IP experiments demonstrated that Flag-TTLL12 effectively precipitated eEF1A1 upon transfection into Hep3B cells (Fig. 3B). Similarly, eEF1A1 was able to reciprocally precipitate Flag-TTLL12 (Fig. 3C). Additionally, GST pull-down assays using GST-TTLL12 and His-eEF1A1, both expressed in *Escherichia coli*, confirmed efficient binding between the two proteins (Fig. 3D). These findings strongly suggest a direct interaction between TTLL12 and eEF1A1.

To further identify the specific interaction sites within eEF1A1 that engage with TTLL12, eEF1A1 was divided into three structural domains based on prior studies. Co-IP experiments indicated that domain 3 (D3) of eEF1A1, encompassing amino acids (aa) 332–462, harbors the interaction site for TTLL12 (Fig. 3E–H). For a more precise localization of the binding site, eEF1A1 was subdivided into smaller structural domains. Co-IP assays revealed that TTLL12 binds to eEF1A1 specifically within the segment spanning aa 438–462 (Fig. 3I, J). Subsequently, the eEF1A1 region from aa 367–462 was further divided into three structural domains. Co-IP assays confirmed that the TTLL12 interaction region is localized within aa 438–462 of eEF1A1 (Fig. 3K). Taken together, these results provide compelling evidence that the interaction between TTLL12 and eEF1A1 is mediated specifically through the D3 region of eEF1A1, particularly the segment encompassing aa 438–462.

### TTLL12 regulates eEF1A1 expression through ubiquitination pathway

**Fig. 4:**
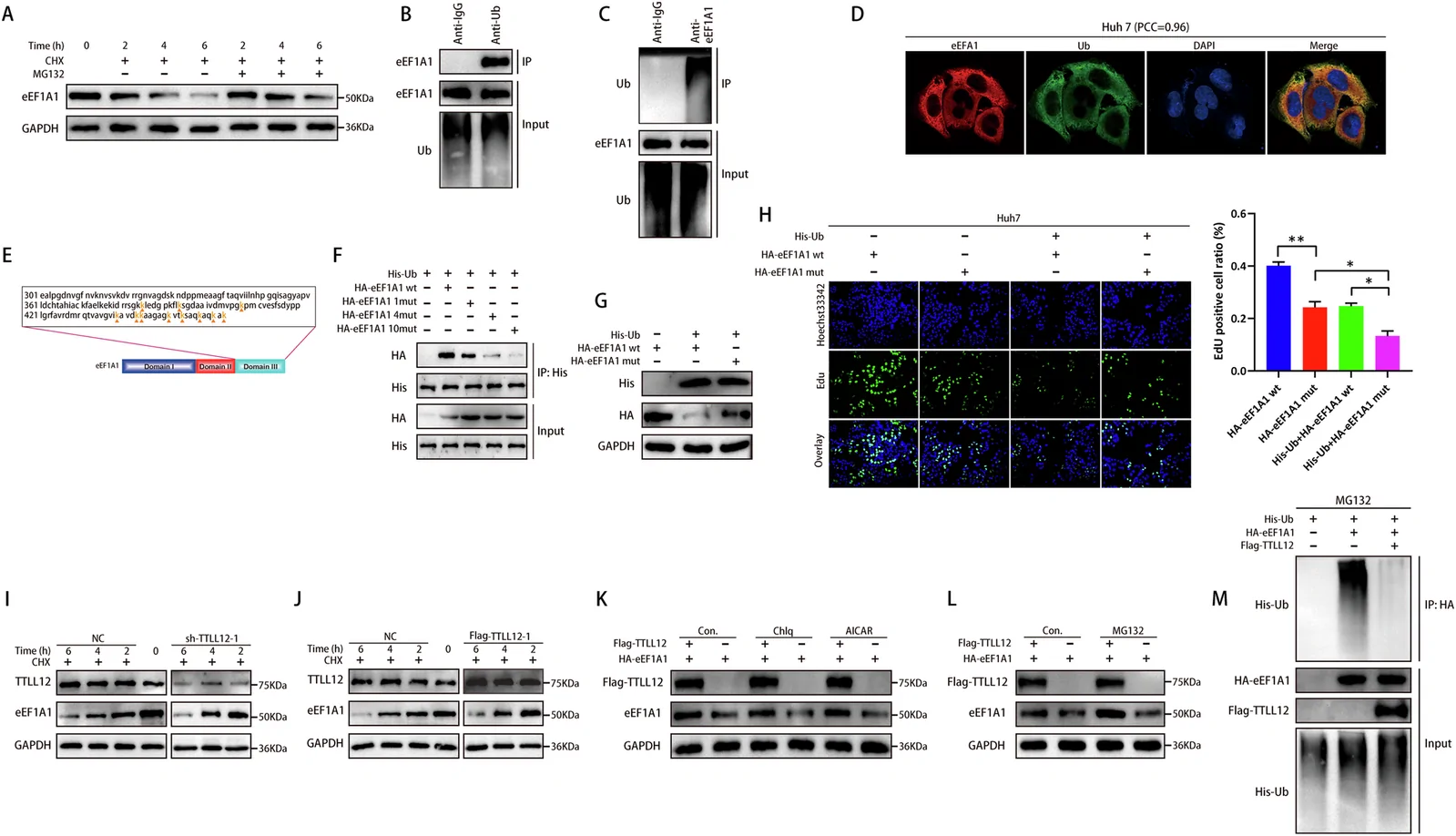
TTLL12 regulates eEF1A1 expression through ubiquitination pathway. **A** Stability of eEF1A1 protein in Huh7 cells treated with CHX at indicated intervals. **B**, **C** Co-IP between endogenous Ubiquitin (Ub) and eEF1A1. **D** Co-localization of Ub and eEF1A1 demonstrated by immunofluorescent staining. **E**, **F** HEK293 cells transfected with indicated constructs; cells lysed for IP using anti-His beads to detect HA binding. **G** Huh7 cells transfected with indicated plasmids and treated with MG132. Detection of eEF1A1 ubiquitination using His antibody. **H** Cell proliferation capacity assessed by EdU assays after Huh7 cells transfected with indicated plasmids. **I**, **J** Analysis of the effect of silencing or overexpressing TTLL12 on the half-life of eEF1A1 in cells treated with CHX at indicated times. **K**, **L** Various inhibitors used to examine the effect on eEF1A1 degradation. **M** Ubiquitination assays of eEF1A1 in lysates from TTLL12-overexpressing cells.

Research has revealed that eEF1A1 undergoes degradation through the ubiquitin- proteasome system (UPS), a process dependent on the conjugation of ubiquitin (Ub) to specific lysine residues. Huh7 cells were treated with MG132 over defined time intervals, during which we monitored eEF1A1 levels. The results indicated that eEF1A1 is prone to degradation via ubiquitination (Fig. 4A). Co-IP assays further demonstrated that Ub could effectively precipitate eEF1A1 when co-transfected into Huh7 cells (Fig. 4B, C). Complementary immunofluorescence assays confirmed the co-localization of Ub and eEF1A1 in the cytoplasm of Huh7 cells (Fig. 4D). To identify the specific lysine residues in eEF1A1 that interact with Ub, we introduced mutations at ten lysine residues in eEF1A1, replacing them with arginine, and generated several mutant plasmids (Fig. 4E). Co-IP assays revealed that eEF1A1 binding to Ub progressively weakened as the number of mutated sites increased, with the eEF1A1-Ub interaction completely abolished when all ten lysine residues were mutated (Fig. 4F).

To further investigate the impact of these lysine residues on eEF1A1 function, Huh7 cells were co-transfected with a His-Ub plasmid and either wild-type (wt) HA-eEF1A1 or mutant HA-eEF1A1 (10mut) plasmids. In the wt eEF1A1 group, upregulation of His-Ub led to decreased eEF1A1 protein levels (Fig. 4G). In contrast, cell proliferation remained stable in the eEF1A1 mutant group (Fig. 4H). These findings underscore the critical role of disrupting the eEF1A1-Ub interaction in regulating tumor cell proliferation. A dynamic degradation assay showed that TTLL12-knockdown cancer cells exhibited reduced eEF1A1 expression compared to control cells (Fig. 4I), whereas TTLL12-overexpressing cells displayed significantly increased eEF1A1 levels relative to controls (Fig. 4J). To identify the specific degradation pathway regulated by TTLL12, we used various inhibitors, including chloroquine and NH4Cl (lysosomal proteolysis inhibitors), AICAR (macro-autophagy inhibitor), and MG132 (proteasome inhibitor). Notably, only MG132 effectively blocked TTLL12-mediated eEF1A1 degradation (Fig. 4K, L). Co-IP assays further confirmed that increased TTLL12 expression led to a reduction in the ubiquitination of eEF1A1 in Huh7 cells (Fig. 4M). In conclusion, these findings demonstrate that TTLL12 regulates eEF1A1 stability by modulating its ubiquitination and degradation.

### BPOZ-2 interacts with eEF1A1 and promotes its degradation

**Fig. 5:**
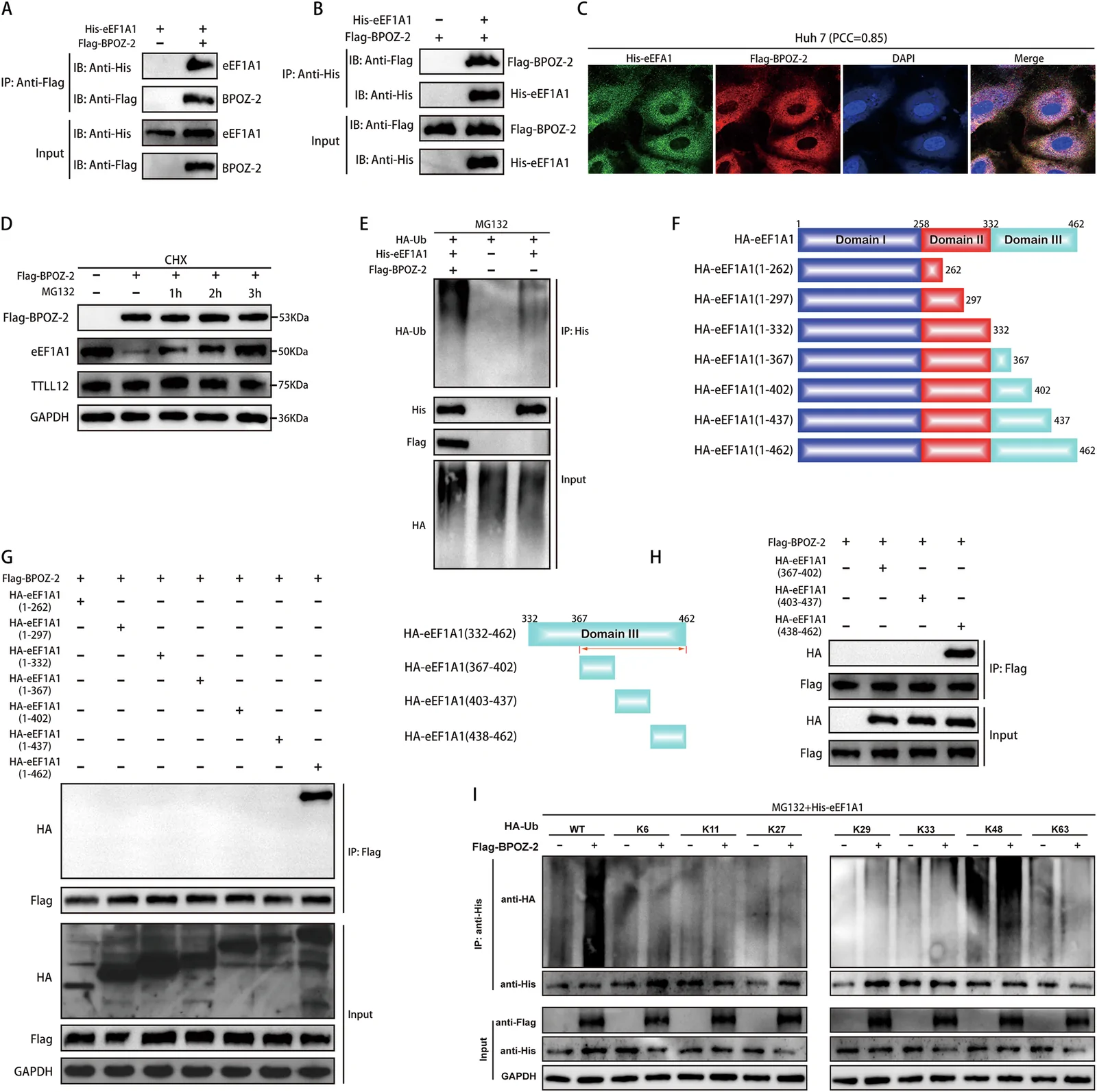
BPOZ-2 interacts with eEF1A1 and promotes its degradation. **A**, **B** Co-IP assays were employed to investigate the interaction between BPOZ-2 and eEF1A1 following co-transfection of Flag-BPOZ-2 and His-eEF1A1 plasmids in HEK293 cells. **C** Immunofluorescent staining of BPOZ-2 and eEF1A1 in Huh7 cells. **D** HEK293 cells were transfected with the Flag-BPOZ-2 plasmid and treated with CHX at the indicated times. The degradation of eEF1A1 was detected by western blotting. **E** Ubiquitination assays of eEF1A1 in lysates from TTLL12-overexpressing cells. **F**–**H** Schematic diagram mapping the eEF1A1 structural domains. Lysates from cells co-transfected with Flag-BPOZ-2 and indicated HA-eEF1A1 constructs were immunoprecipitated with anti-Flag antibodies and subjected to western blotting. **I** HEK293 cells were transfected with His-eEF1A1 and/or Flag-BPOZ-2 together with ubiquitin WT or different ubiquitin mutants. After treatment with MG132 for 6 h, the cells were collected and subjected to IP and western blot assays.

BPOZ-2, a human homolog of yeast Btb3p, functions as a potential substrate-specific adaptor for the E3 ubiquitin ligase Pcu3p [19]. Previous studies have suggested that BPOZ-2 facilitates eEF1A1 ubiquitylation and subsequent degradation [20]. To unravel the mechanisms underlying BPOZ-2-mediated eEF1A1 degradation, we examined the potential interaction between BPOZ-2 and eEF1A1. The outcomes revealed that Flag- BPOZ-2 could co-precipitate with His-eEF1A1 (Fig. 5A). Similarly, His-eEF1A1 had the ability to pull down Flag-BPOZ-2 (Fig. 5B). Immunofluorescence assays further substantiated this interaction by demonstrating the co-localization of BPOZ-2 with eEF1A1 in the cytoplasm of Huh7 cells, signifying their close association within these cellular compartments (Fig. 5C). Subsequently, we examined whether BPOZ-2 facilitates the degradation of eEF1A1 under conditions of protein synthesis inhibition induced by CHX. Specifically, eEF1A1 levels were monitored in HEK293 cells over a 3-h period under various experimental conditions: with or without the proteasome inhibitor MG132, and in the presence or absence of BPOZ-2 overexpression. Notably, the overexpression of BPOZ-2 inhibited the degradation of eEF1A1 following CHX treatment in the presence of MG132 (Fig. 5D). Furthermore, BPOZ-2 overexpression led to a significant increase in the ubiquitination of eEF1A1 (Fig. 5E). These findings suggest that BPOZ-2 acts as a facilitator of eEF1A1 ubiquitination and subsequent degradation.

To further explore the interaction between BPOZ-2 and eEF1A1, we dissected eEF1A1 into several structural domains, as previously described (Fig. 5F). Co-IP assays revealed that the eEF1A1 region spanning aa 438–462 mediates the interaction with BPOZ-2 (Fig. 5G). Additional segmentation of the eEF1A1 region between aa 367–462 confirmed the aa 438–462 domain as the critical region for BPOZ-2 binding (Fig. 5H). And overexpression of BPOZ-2 significantly promotes K48-linked ubiquitination of eEF1A1 (Fig. 5I). Collectively, these results suggest that BPOZ-2 regulates both the ubiquitination and stabilization of eEF1A1 via specific interactions within this defined region.

### TTLL12 competes with BPOZ-2 to bind to eEF1A1

**Fig. 6:**
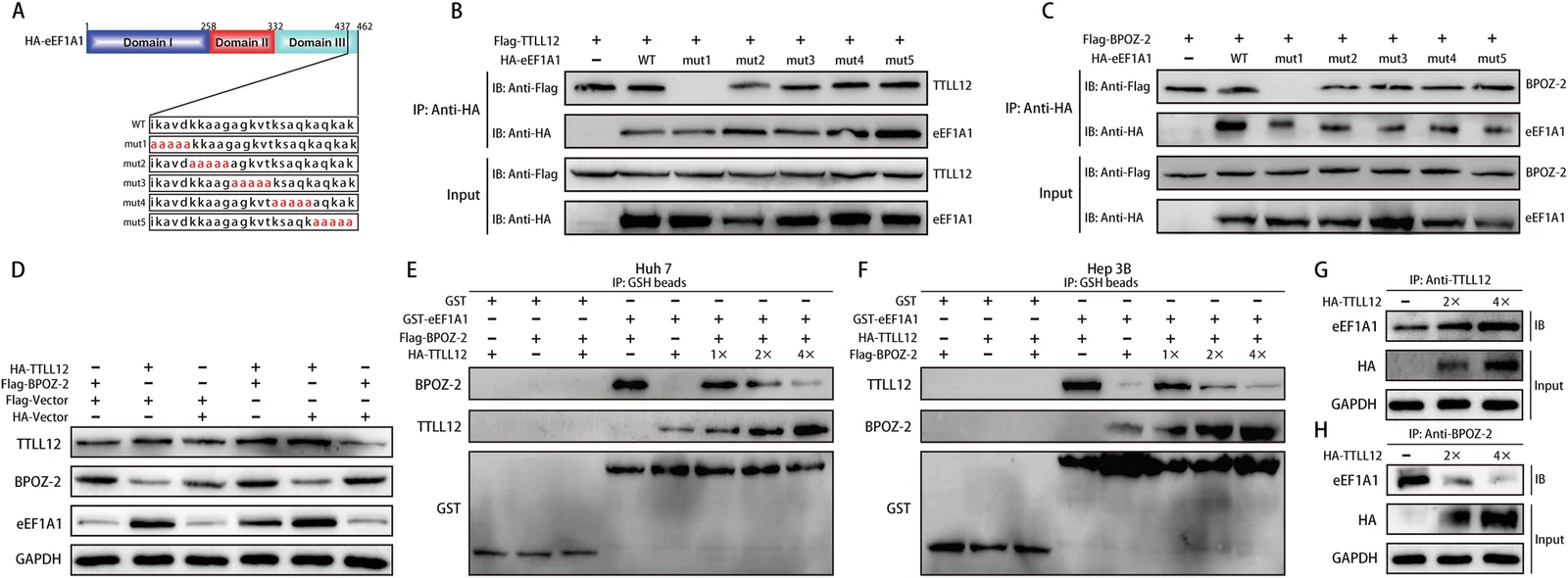
TTLL12 competes with BPOZ-2 to bind to eEF1A1. **A**–**C** HEK293 cells were transfected with indicated constructs, and cells were lysed for Co-IP using anti-HA beads to detect Flag binding. **D** HEK293 cells were co-transfected with indicated plasmids, and western blot analyses were performed to examine the eEF1A1 protein level. **E**, **F** The binding of eEF1A1 during the course of the competition was analyzed by GST pull-down experiment. **G**, **H** Huh7 cells were transfected with increasing amounts of HA-TTLL12 plasmid. The cells were lysed for IP to detect eEF1A1 binding.

Based on the aforementioned data, it is evident that TTLL12 and BPOZ-2 share a common binding site within the eEF1A1 region. To further define the eEF1A1-binding region, five alanine-scanning mutants (mut1, mut2, mut3, mut4, and mut5) were generated (Fig. 6A). Remarkably, Co-IP analyses revealed that mut1 failed to interact with the TTLL12 protein, whereas the binding activity of the other four mutants remained unaffected (Fig. 6B, C). Additionally, a degradation dynamics assay showed that the ectopic expression of TTLL12 significantly increased eEF1A1 levels compared to control cells (Fig. 6D). In contrast, overexpression of BPOZ-2 hindered the TTLL12-induced increase in eEF1A1 expression. These combined results underscore the competitive nature of TTLL12 and BPOZ-2 binding to the identical lysine sites on eEF1A1. Additionally, GST pull-down assays confirmed the competitive binding of TTLL12 and BPOZ-2 to eEF1A1 in both Huh7 and Hep3B cells (Fig. 6E, F). Our findings further demonstrated that increasing the amount of TTLL12 plasmid led to a dose-dependent rise in TTLL12-eEF1A1 complex formation, accompanied by a corresponding decrease in BPOZ-2-eEF1A1 complexes. Conversely, elevating BPOZ-2 levels resulted in a reduction in TTLL12-eEF1A1 complexes while promoting the formation of BPOZ-2-eEF1A1 complexes in Huh7 cells (Fig. 6G, H). These results provide strong evidence of a competitive binding relationship between TTLL12 and BPOZ-2 for eEF1A1, where the overexpression of TTLL12 enhances TTLL12-eEF1A1 complex formation while simultaneously reducing the association between BPOZ-2 and eEF1A1.

### Upregulation of TTLL12 correlates with poor prognosis in HCC

**Fig. 7:**
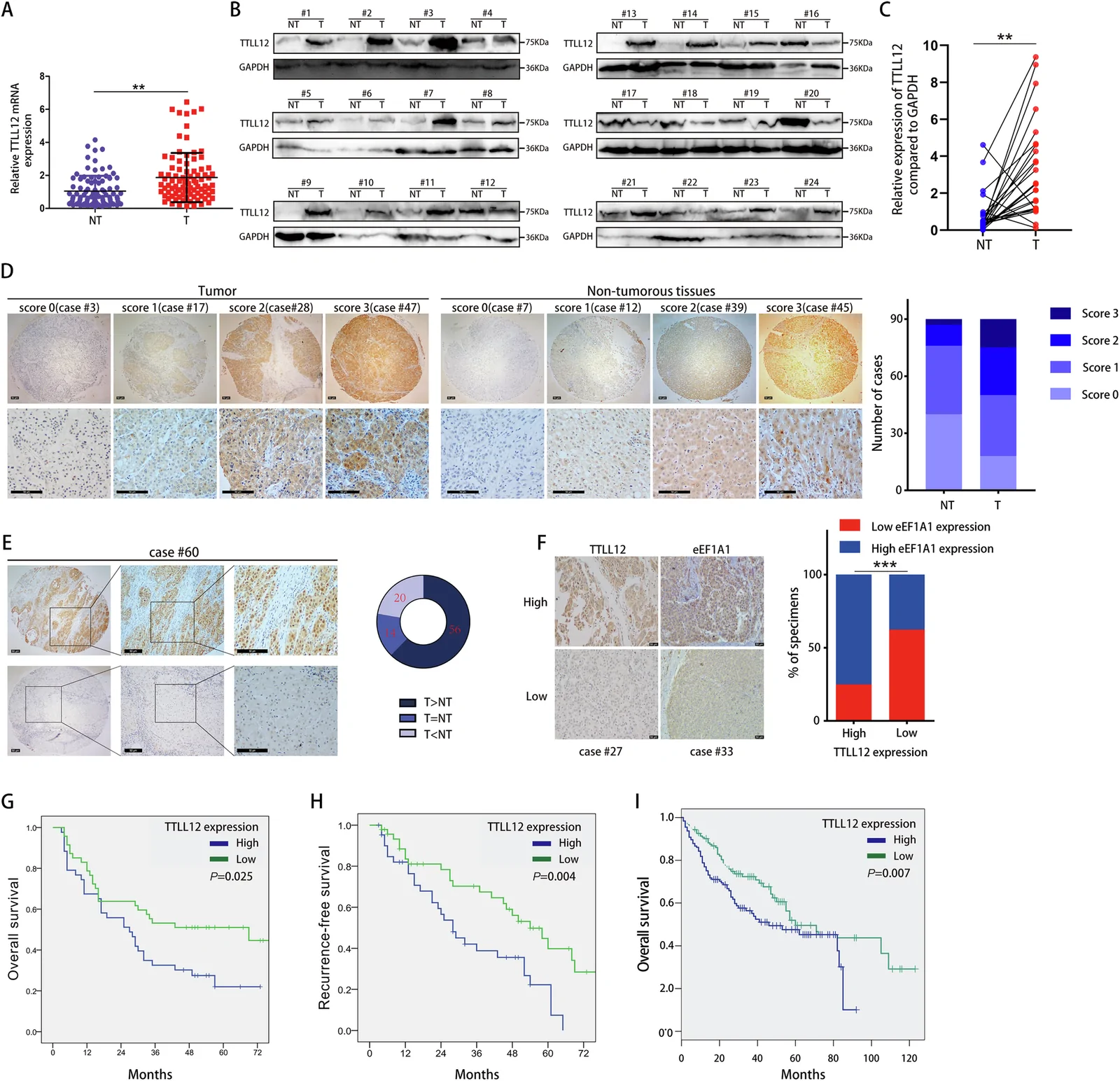
Upregulation of TTLL12 correlates with poor prognosis in HCC. **A** QRT-PCR analysis of relative TTLL12 mRNA expression in 90 cases of HCC tissue samples. **B**, **C** Immunoblotting for TTLL12 protein in HCC patients. Presented are 24 randomly selected pairs of HCC tumors and adjacent normal tissues. **D**, **E** IHC staining of TTLL12 in representative carcinoma and the surrounding tissues of clinical HCC samples. **F** Correlation analysis of eEF1A1 with TTLL12 protein in selected HCC patients by IHC staining. **G**–**I** Kaplan–Meier survival curves of HCC patients with low and high TTLL12 expression in (A) and TCGA database.

To evaluate the prognostic significance of TTLL12 in HCC patients, we performed RT-qPCR analysis to quantify TTLL12 transcription levels in 90 paired human HCC tissues and their corresponding non-tumorous counterparts. The analysis revealed a significant elevation of TTLL12 mRNA expression in HCC tissues compared to adjacent normal tissues (Fig. 7A), consistent with data from the Cancer Genome Atlas (TCGA) database (Supplementary Fig. S5). This upregulation at the mRNA level was corroborated by immunoblotting assays performed on 24 paired samples, which further confirmed increased TTLL12 expression at the protein level (Fig. 7B, C). In addition, patient characteristics according to the TTLL12 expression were shown in Supplementary Table S3. High expression of TTLL12 was associated with more advanced tumor stage. In this cohort, a score of 3 was observed in only 3 non-tumorous tissues, compared to 15 HCC tissues, indicating a notable increase in TTLL12 expression in tumor samples (Fig. 7D). Overall, 62.22% (56/90) of cases exhibited elevated TTLL12 expression (Fig. 7E). To further explore the relationship between TTLL12 and eEF1A1, we examined another panel of HCC samples. In 20 out of 32 cases (62.5%) with high TTLL12 expression, strong eEF1A1 expression was also detected. In contrast, only 5 out of 20 cases (25%) with low TTLL12 expression exhibited high eEF1A1 levels (Fig. 7F). Moreover, Kaplan-Meier survival analysis showed that patients with elevated TTLL12 expression had significantly poorer survival outcomes compared to those with lower expression levels (Fig. 7G, H), a trend that was consistent with observations from TCGA database (*P* < 0.05) (Fig. 7I). Collectively, these findings indicate that increased TTLL12 expression is associated with tumor progression and serves as a valuable prognostic marker for poor outcomes in HCC.

**Fig. 8:**
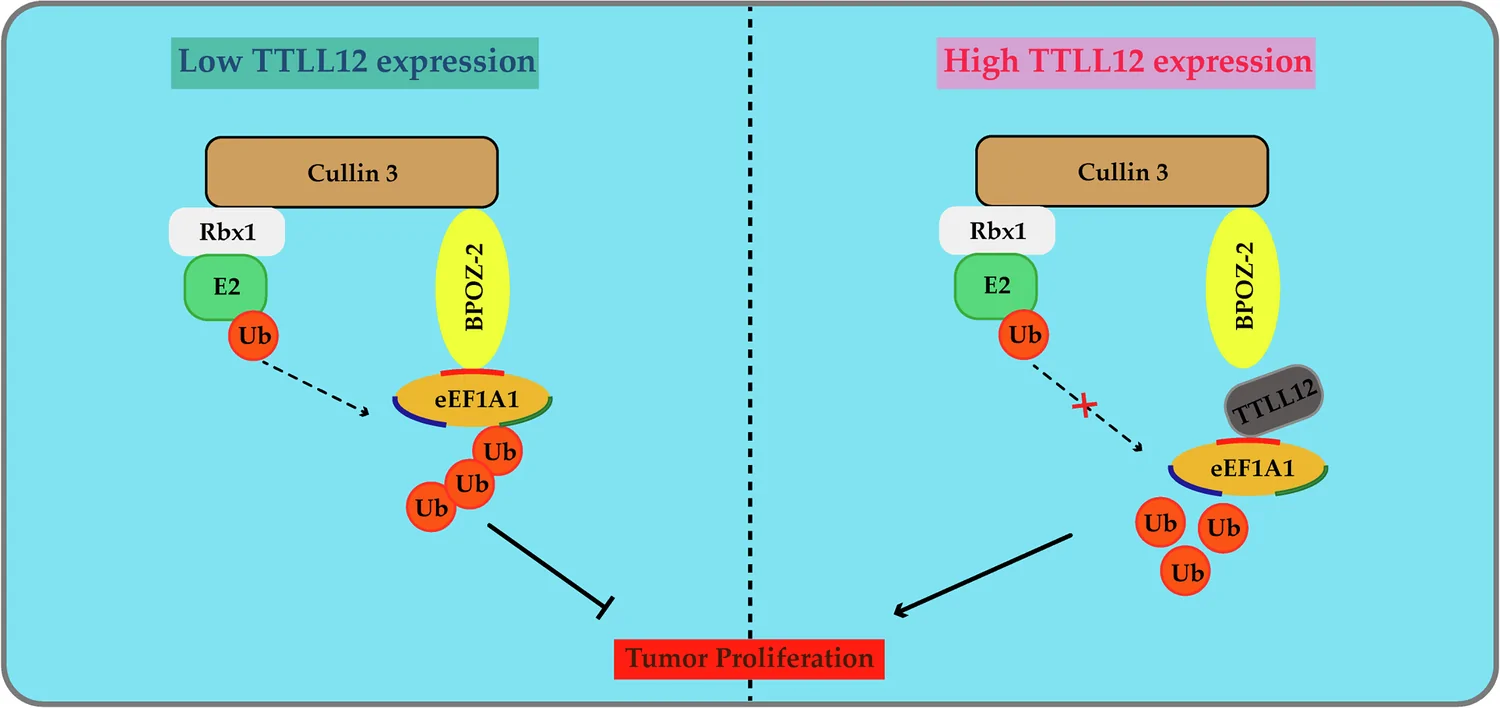
A proposed model illustrating the role of TTLL12 in HCC. Overexpression of TTLL12 results in a simultaneous reduction in BPOZ-2-eEF1A1 complexes and an increase in TTLL12-eEF1A1 complexes. TTLL12 inhibits eEF1A1 ubiquitination through reduced BPOZ-2-eEF1A1 complexes. Notably, TTLL12-eEF1A1 complexes remain resistant to degradation by the proteasome.

Based on these results, we propose a model in which TTLL12 promotes cancer cell proliferation through inhibiting BPOZ-2-mediated ubiquitination and degradation of eEF1A1 (Fig. 8).

## Discussion

Tumor proliferation is a critical driver of cancer progression, a phenomenon that is well-documented in HCC. The unchecked and accelerated growth of cancerous cells underscores the importance of elucidating the regulatory mechanisms that govern tumor proliferation, as such insights are pivotal for developing effective strategies in cancer prevention and treatment. In our investigation, we observed a marked upregulation of TTLL12 expression in HCC tissue samples, with elevated levels of TTLL12 significantly correlating with poorer patient survival outcomes. eEF1A1, a protein responsible for initiating protein translation elongation, plays a fundamental role in regulating various cellular processes. Previous studies have demonstrated that eEF1A1 is overexpressed in multiple types of tumors, where its elevated expression is associated with enhanced cancer cell proliferation and progression [21, 22]. Additionally, research has revealed that BPOZ-2 directly interacts with eEF1A1 to modulate its expression [20]. Our study discovered that both TTLL12 and eEF1A1 were upregulated in HCC cells, and further investigation unveiled that TTLL12 can bind to eEF1A1, thereby impacting eEF1A1 expression. Knockdown of TTLL12 led to a significant reduction in eEF1A1 expression, which in turn resulted in decreased cancer cell proliferation. Conversely, overexpression of eEF1A1 counteracted the reduced proliferation caused by TTLL12 knockdown. Our research unveils a novel mechanism through which TTLL12 promotes HCC cell proliferation by upregulating eEF1A1 expression, highlighting the potential of TTLL12 as a diagnostic biomarker and a promising therapeutic target for HCC.

TTLL12, belonging to the TTLL family, demonstrates remarkable conservation across phylogeny, and it holds a pivotal role in catalyzing posttranslational modifications of tubulin proteins [23]. Recent advancements in the elucidation and characterization of the TTLL family have unveiled intriguing prospects for novel discoveries, particularly in the context of cellular regulation and cancer biology [8]. Several studies have illuminated TTLL12’s involvement in cancer development and its emergence as a promising target for anti-tumor therapy [24, 25]. However, despite these advances, the precise regulatory mechanisms by which TTLL12 contributes to HCC remain poorly understood, highlighting the need for more tailored molecular therapeutics directed at tubulin and its modifications. In the current study, we have discerned that TTLL12 is a key regulator of eEF1A1 expression within the milieu of HCC. More specifically, TTLL12 engages in a competitive interaction with BPOZ-2 to bind with eEF1A1, which effectively impedes the ubiquitination and degradation of eEF1A1 induced by BPOZ-2, culminating in the potentiation of HCC cell proliferation. These findings establish a mechanistic link between TTLL12 and eEF1A1, providing valuable insights into the role of TTLL12 in driving tumor progression.

Translation stands as the pivotal process governing protein synthesis within cells, and dysregulation within translational mechanisms frequently underlies the etiology of cancers [26]. In particular, translation factors, acting as critical orchestrators in the translation process, have emerged as potential oncoproteins that possess the capacity to facilitate cellular transformation through the introduction of translation inaccuracies, interaction with cytoskeletal alterations, and modulation of signaling pathways [27]. Of these factors, eEF1A1 occupies a central role as an evolutionarily conserved and fundamental non-ribosomal component within the translational machinery, and it represents as one of the most abundant proteins constituting eukaryotic proteomes [28]. Recently, the mechanisms governing the expression of eEF1A1 have garnered considerable attention [29]. Prior research has suggested increased eEF1A1 expression in HCC cell lines relative to the normal surrounding tissue [30]. Nonetheless, the regulatory underpinnings of eEF1A1 within HCC remain largely unknown. Thus, this study attempts to unravel the mechanism governing the impact of TTLL12 on eEF1A1 expression. Our results indicate that TTLL12 enhances the stability of eEF1A1 by countering its ubiquitination and subsequent degradation. A prior study has already established an association between BPOZ-2 and eEF1A1 [20]. Consequently, we postulate that TTLL12 promotes hepatocarcinogenesis by competitively binding to eEF1A1, forming TTLL12-eEF1A1 complexes rather than BPOZ-2-eEF1A1 complexes, thereby enhancing the stability of eEF1A1. Subsequent analyses within our study provide additional support to the hypothesis by revealing that TTLL12 and BPOZ-2 both interact with eEF1A1 at the same lysine residues, underscoring the inverse correlation between the formation of TTLL12-eEF1A1 complexes and BPOZ-2-eEF1A1 complexes. Therefore, our study elucidates a novel mechanism whereby TTLL12 inhibits eEF1A1 ubiquitination, thereby upregulating its expression by reducing the formation of BPOZ-2-eEF1A1 complexes.

While this study elucidates key mechanistic aspects of the TTLL12-eEF1A1 interaction, its limitations must be acknowledged. The experimental evidence derives exclusively from in vitro models using immortalized cell lines, which inherently lack the physiological complexity of native tissue microenvironments, including three-dimensional cellular architecture, dynamic signaling gradients, and systemic regulatory networks. Although rigorous biochemical assays confirm their physical association under controlled conditions, functional validation in in vivo systems remains critical to establish pathological relevance and exclude culture-induced artifacts. Future studies employing tissue-specific knockout models or clinical specimens will bridge this gap between mechanistic discovery and translational applicability.

## Conclusions

In summary, we demonstrate that TTLL12 could promote the progression of HCC by suppressing BPOZ-2-induced eEF1A1 ubiquitination and degradation, which may inaugurate a novel intervention strategy for the management of HCC.

## Supplementary information

Supplementary legends

Table S1

Table S2

Table S3

Figure S1

Figure S2

Figure S3

Figure S4

Figure S5

Original data

## Data Availability

The data substantiating the outcomes of this investigation are obtainable from the corresponding author upon reasonable request.
