# Supplementary material for: TTLL12 counteracts BPOZ-2 to stabilize eEF1A1 and promote hepatocarcinogenesis
Source: Cell Death Dis. 2026 Apr 21;17(1):837. doi: 10.1038/s41419-026-08694-1 (PMC13616918; doi:10.1038/s41419-026-08694-1)
Supplement: Supplementary file 1 [file 41419_2026_8694_MOESM10_ESM.pdf]

Figure 1A

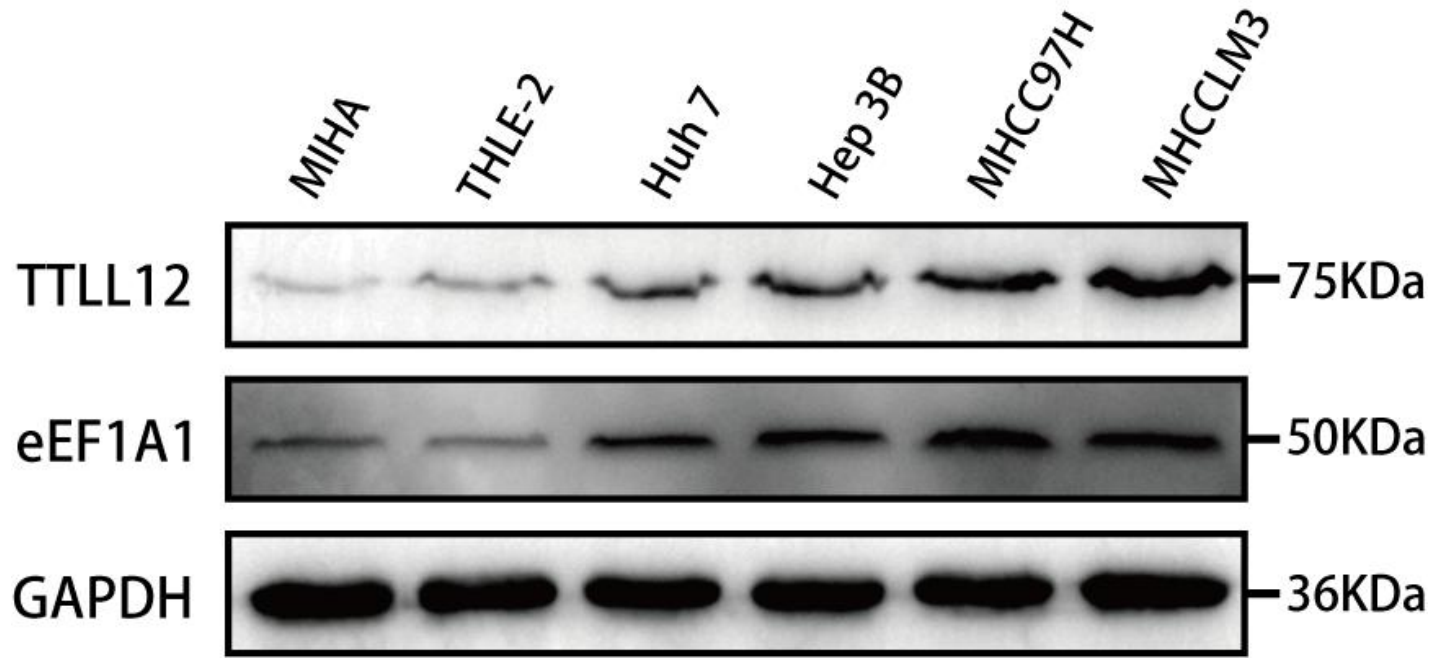

TTLL12

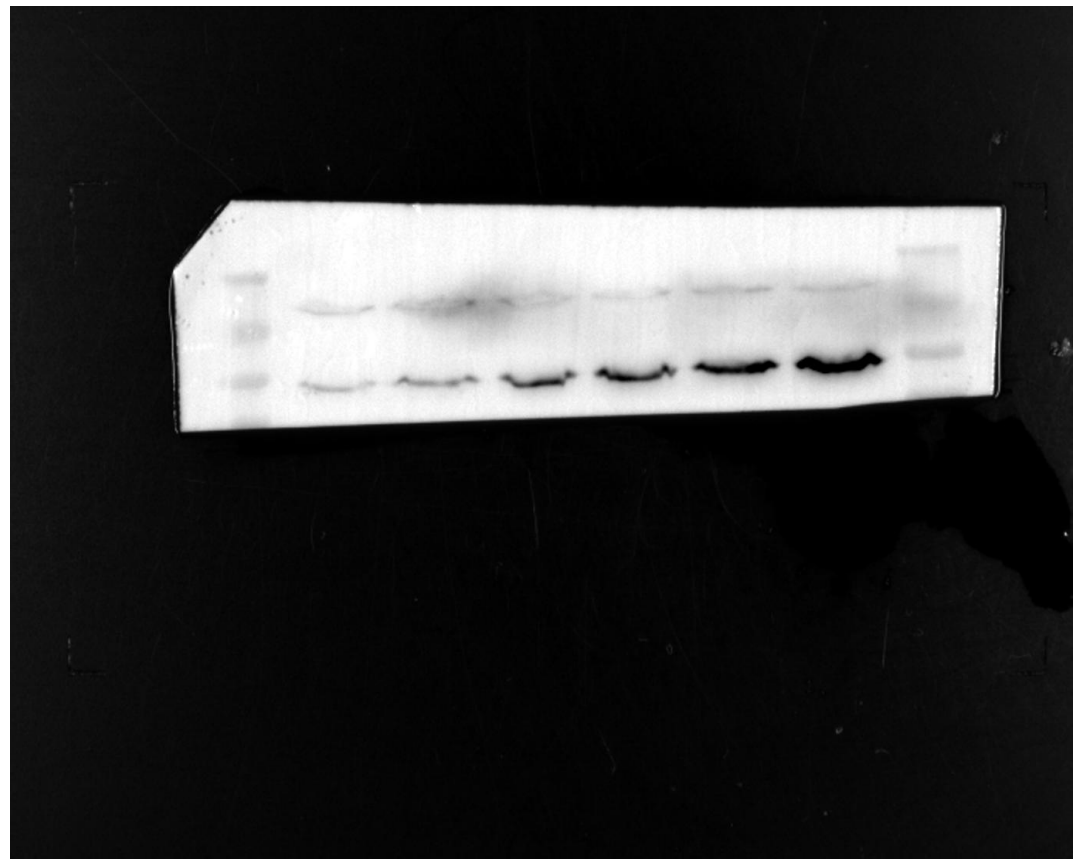

eEF1A1

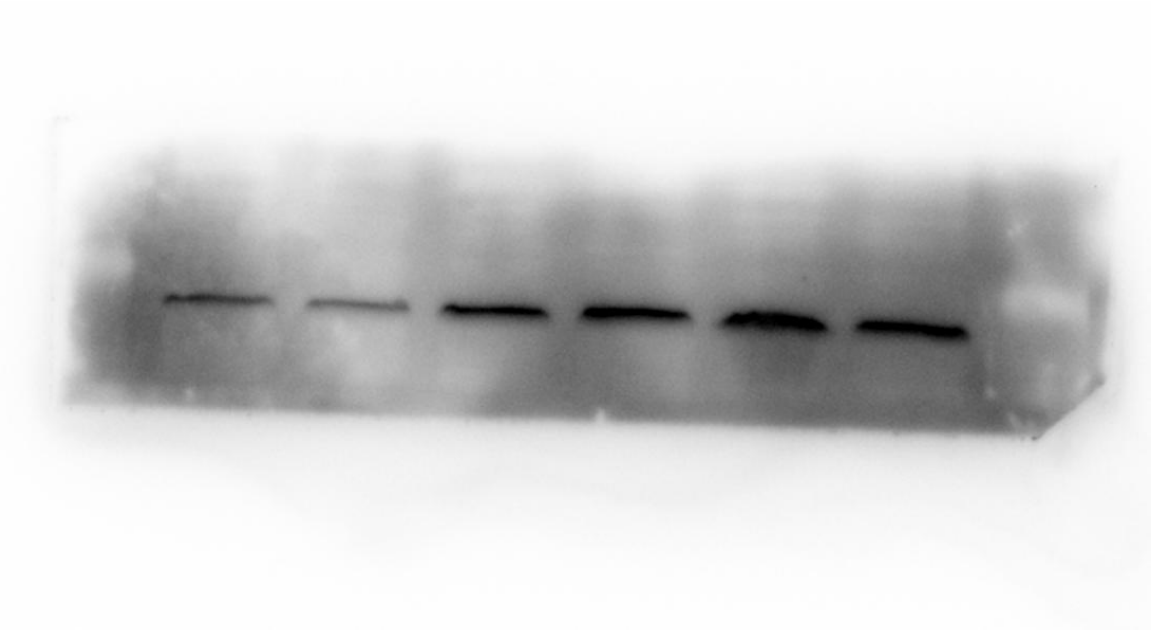

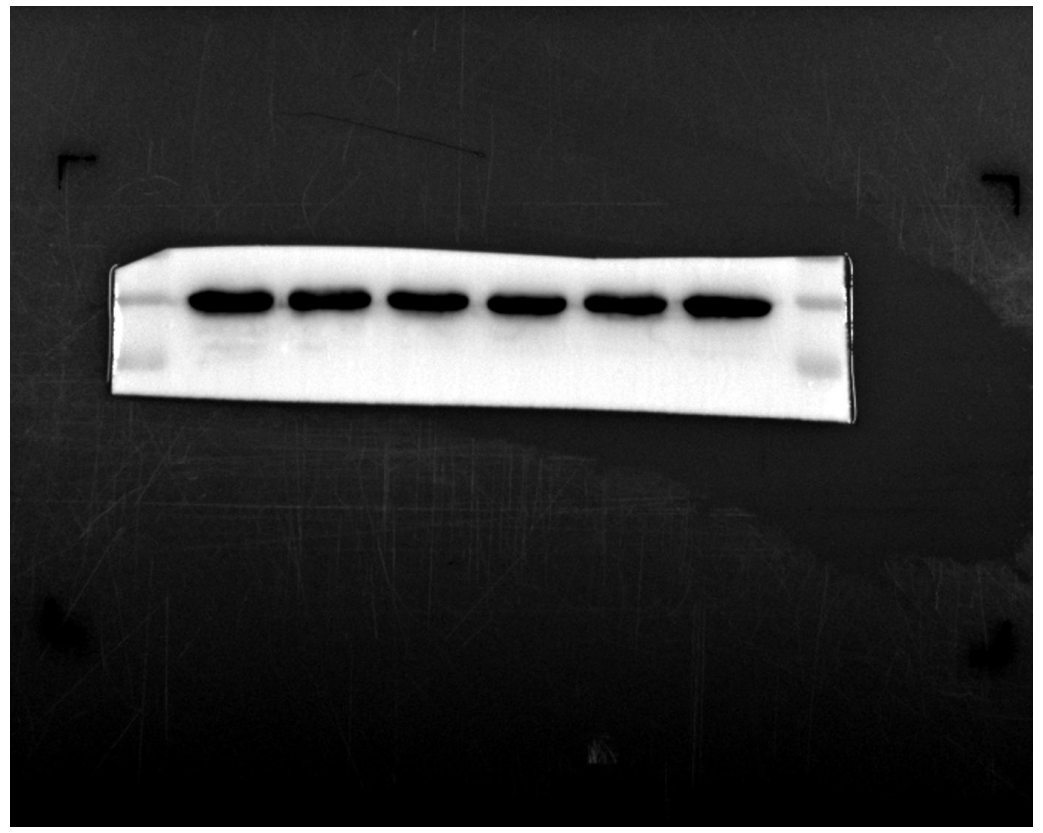

GAPDH

Figure 1B

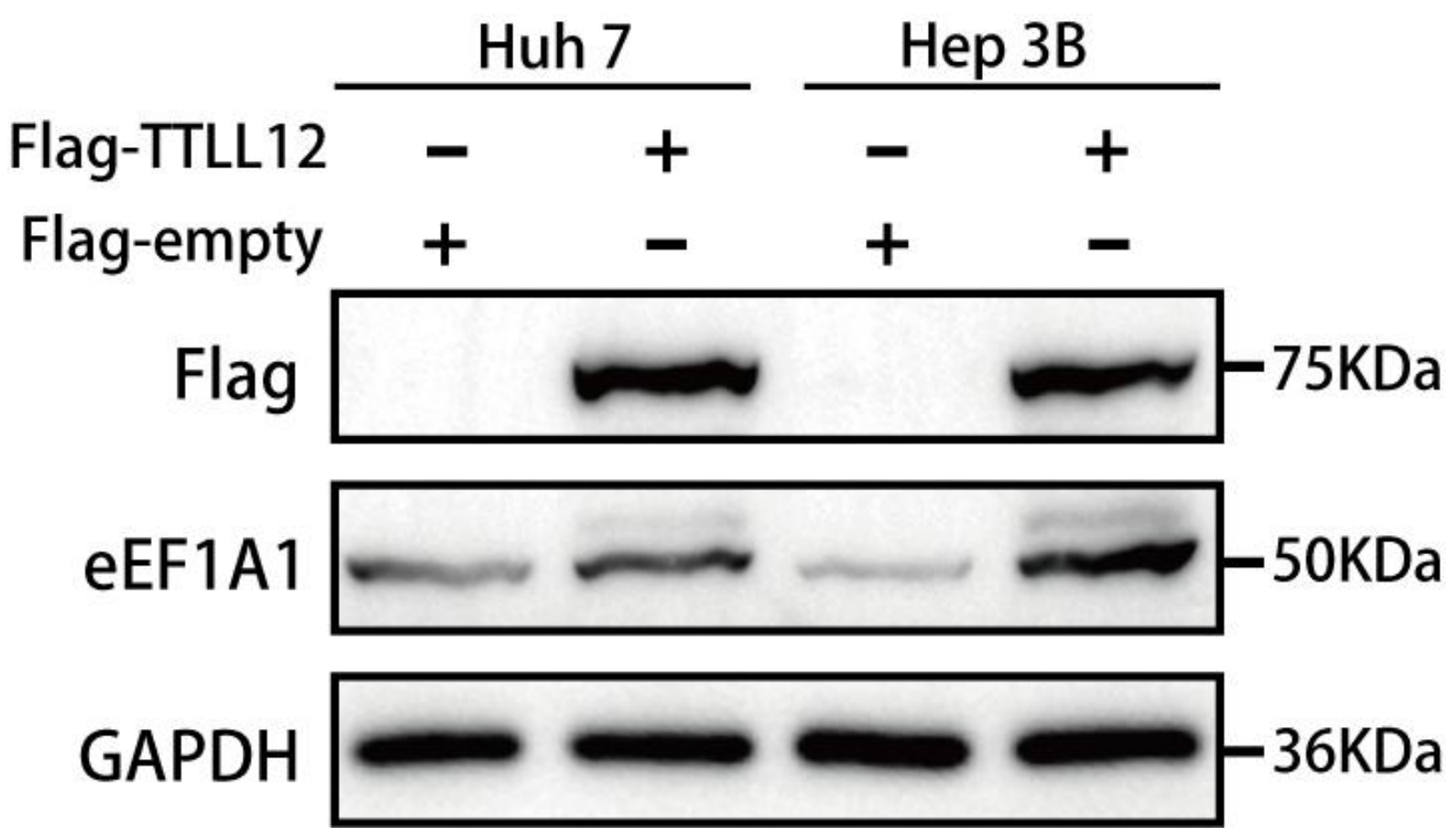

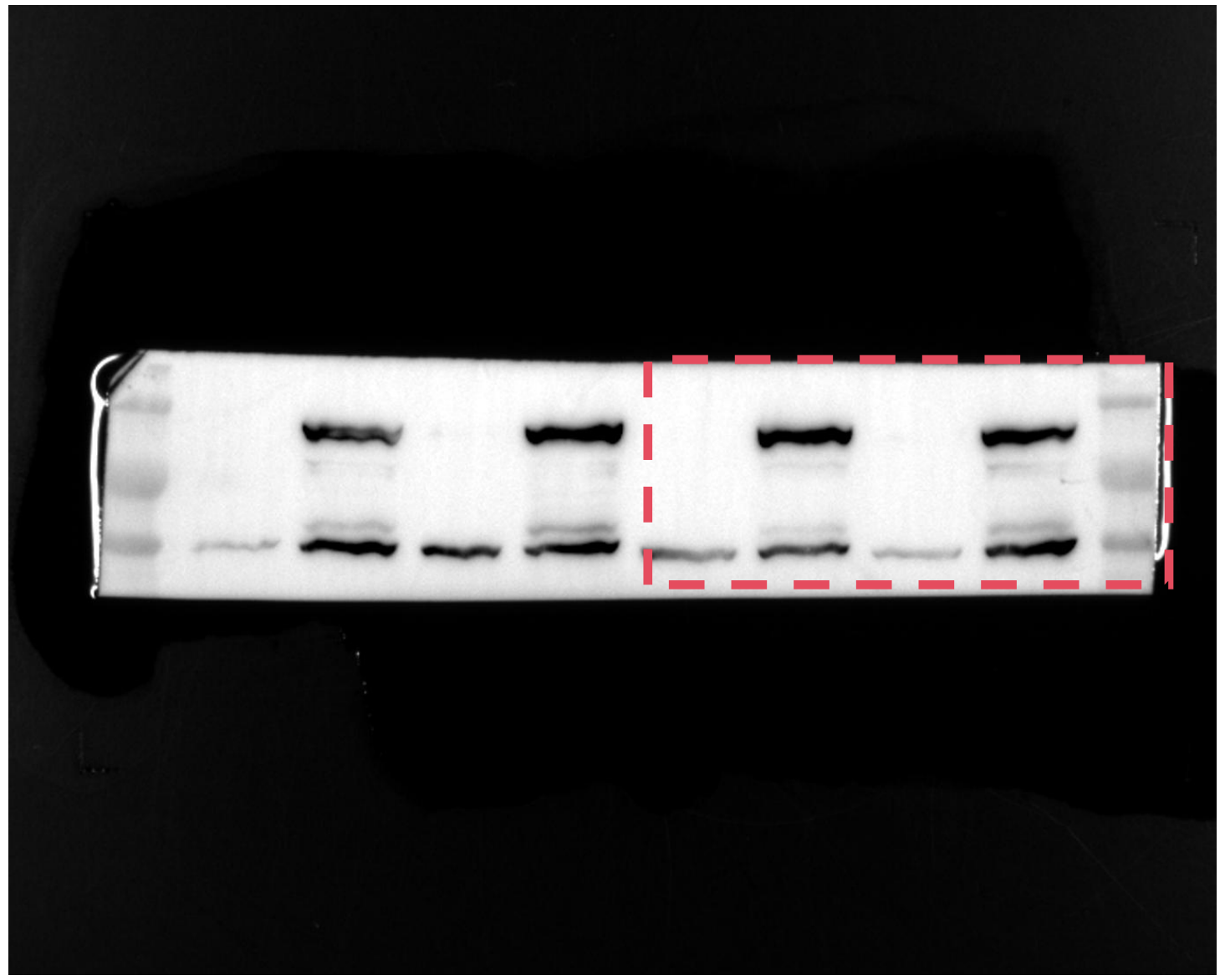

Flag-TTLL12

eEF1A1

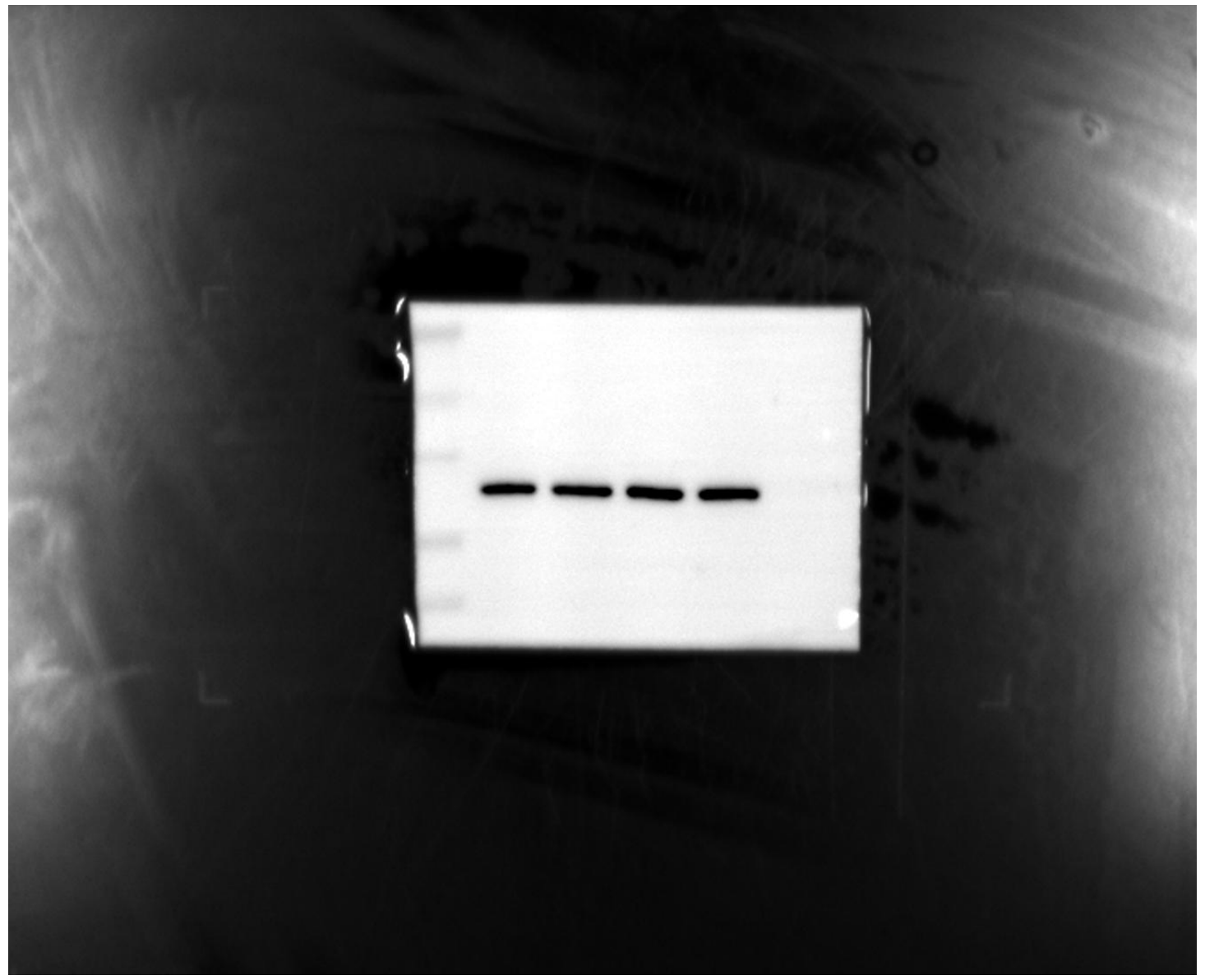

GAPDH

Figure 1C

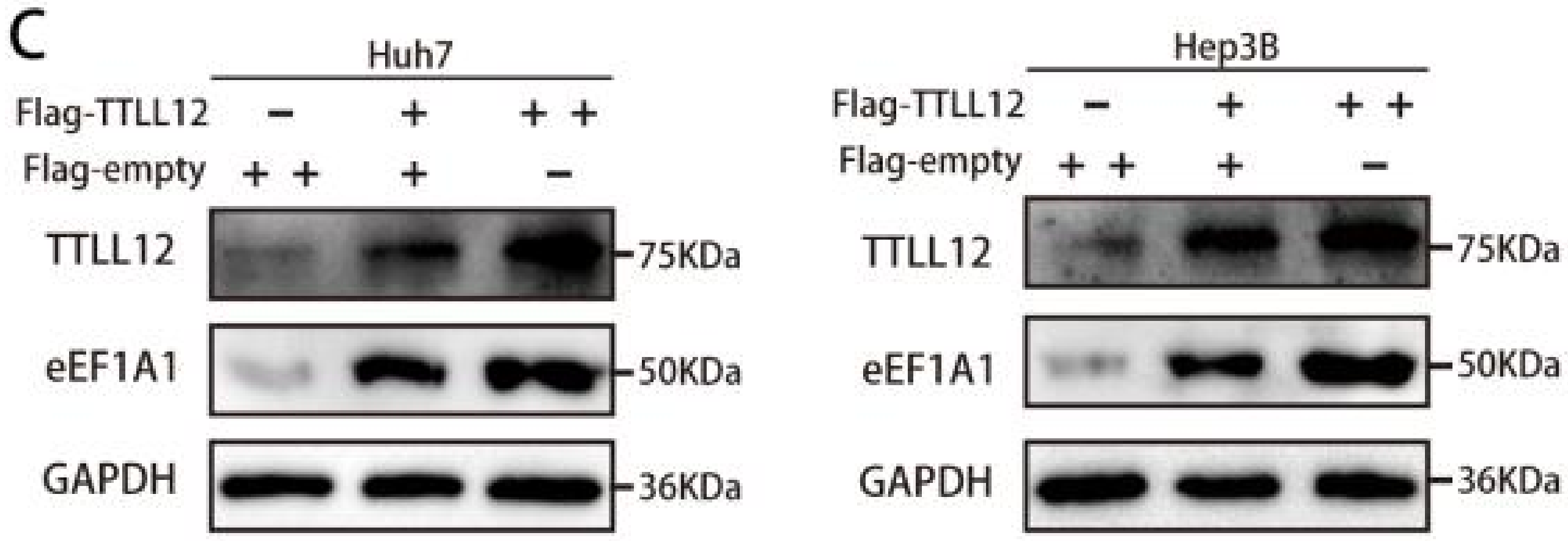

TTLL12

eEF1A1

GAPDH

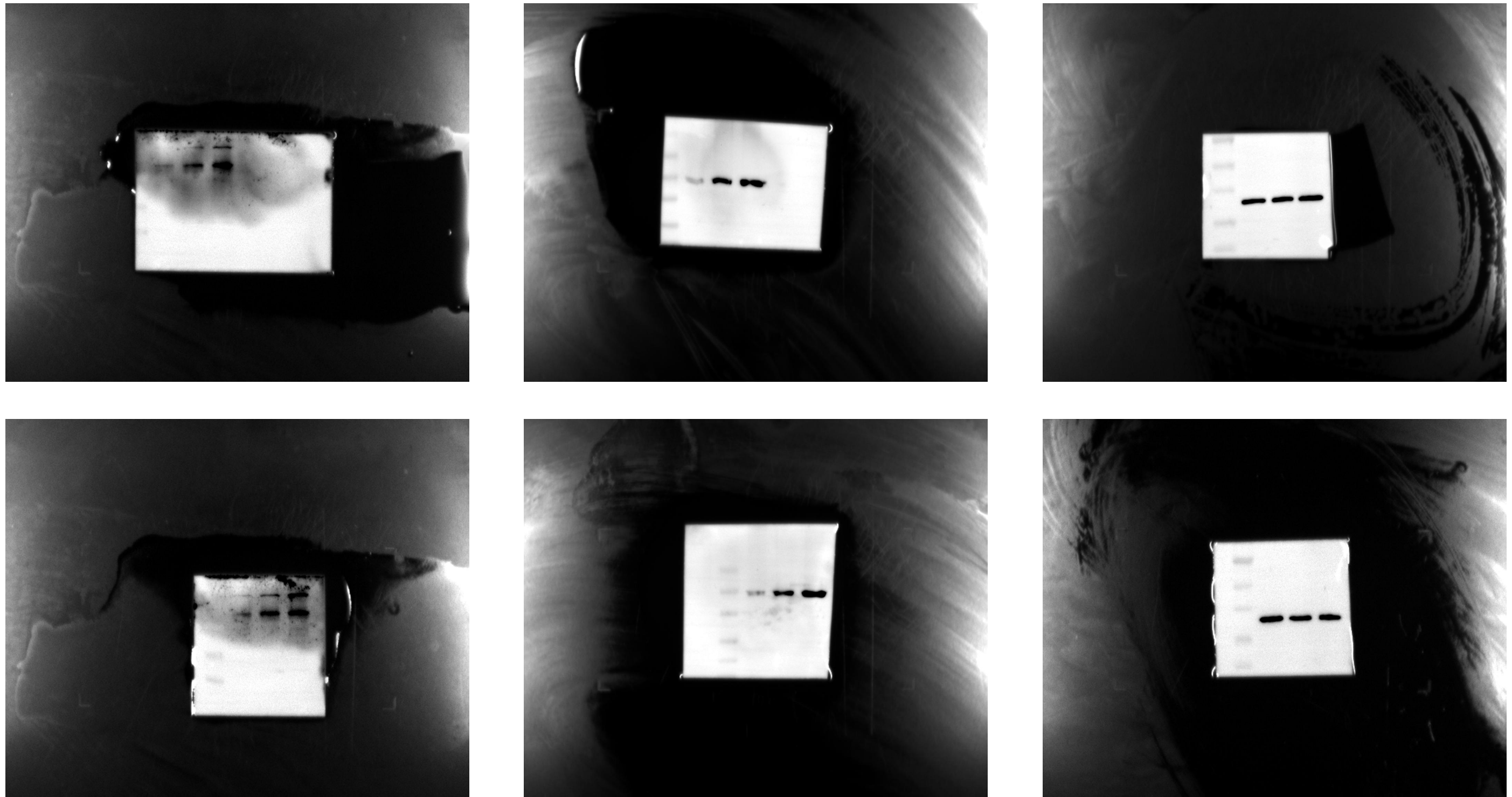

Figure 1D

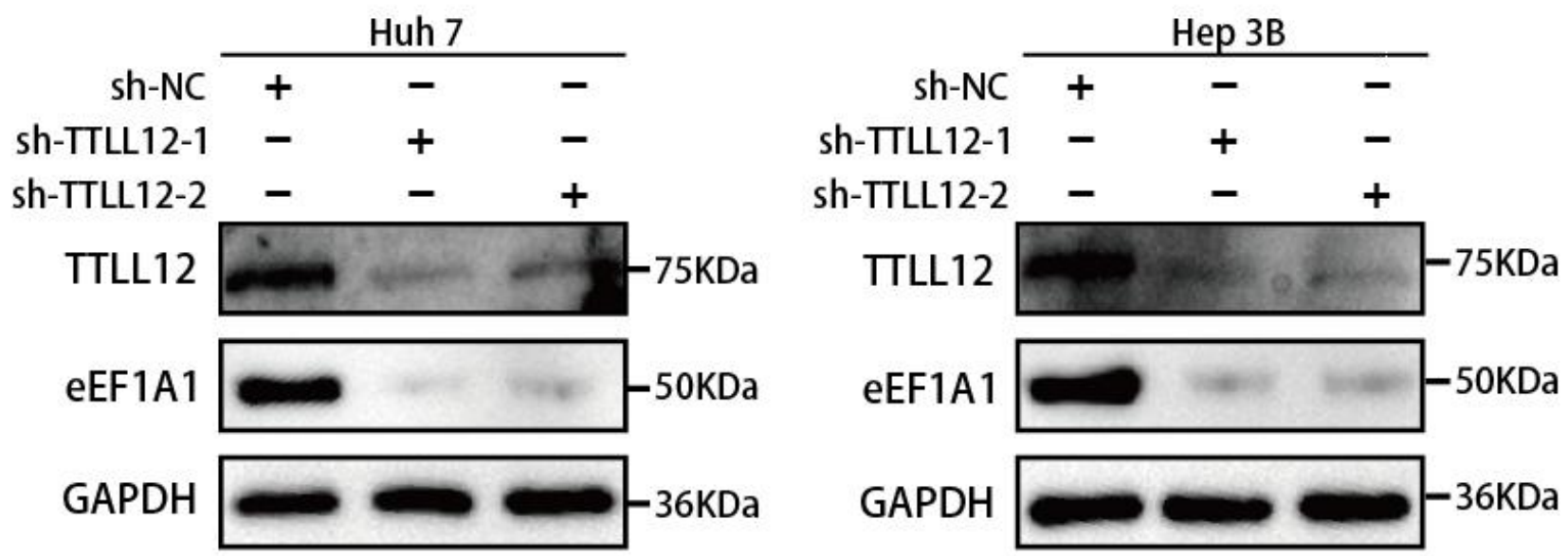

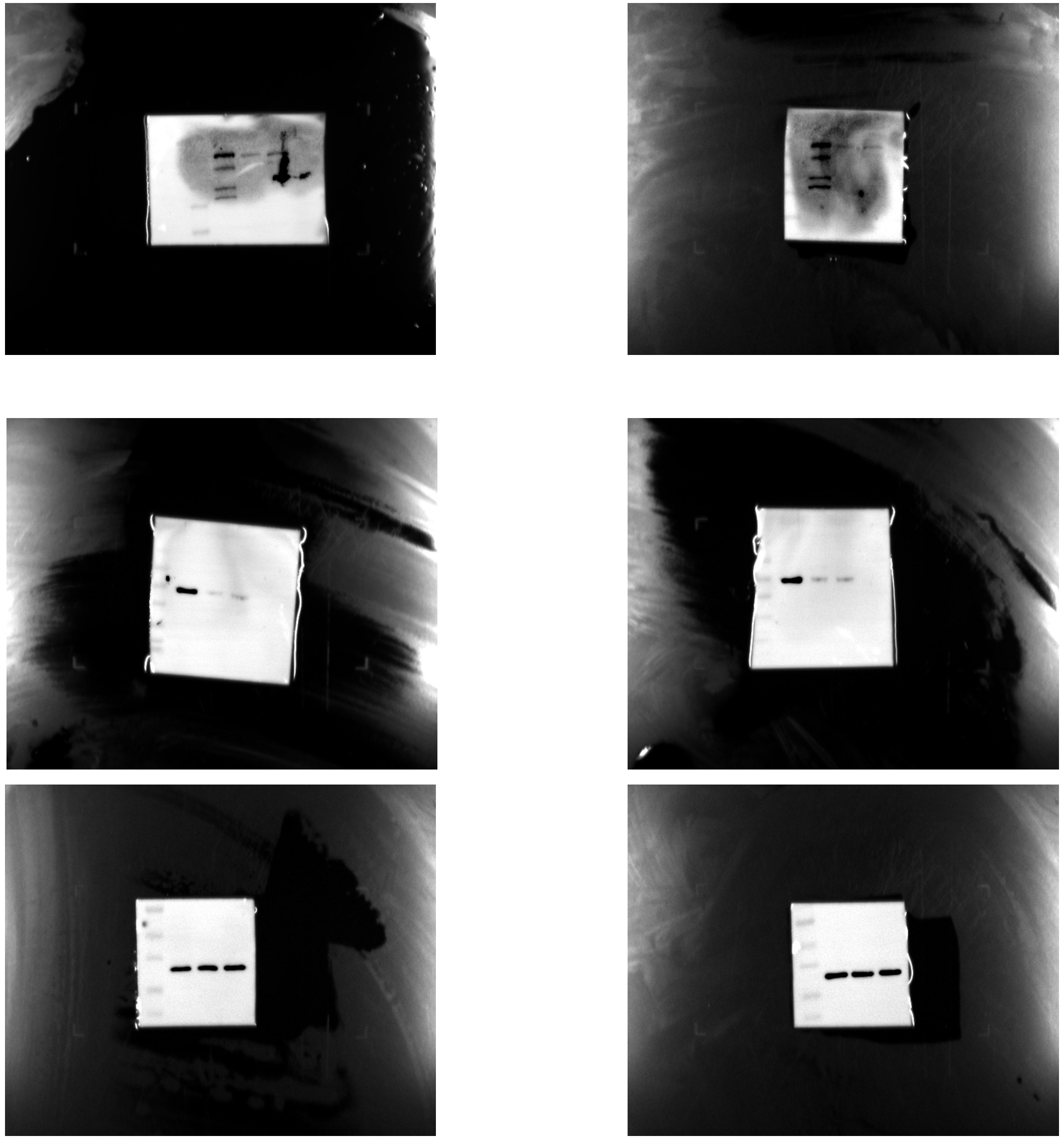

TTLL12

eEF1A1

GAPDH

Figure 2A

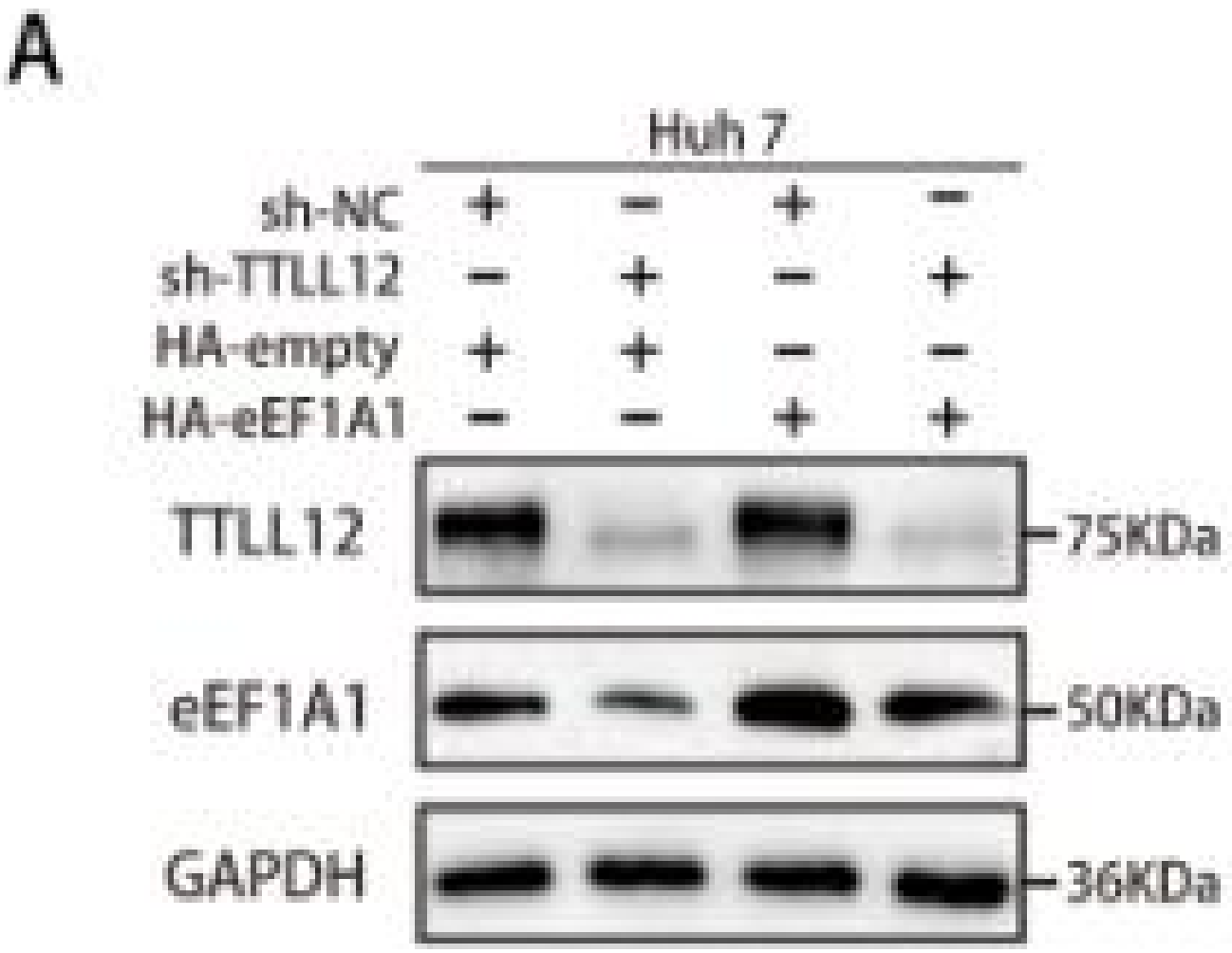

TTLL12

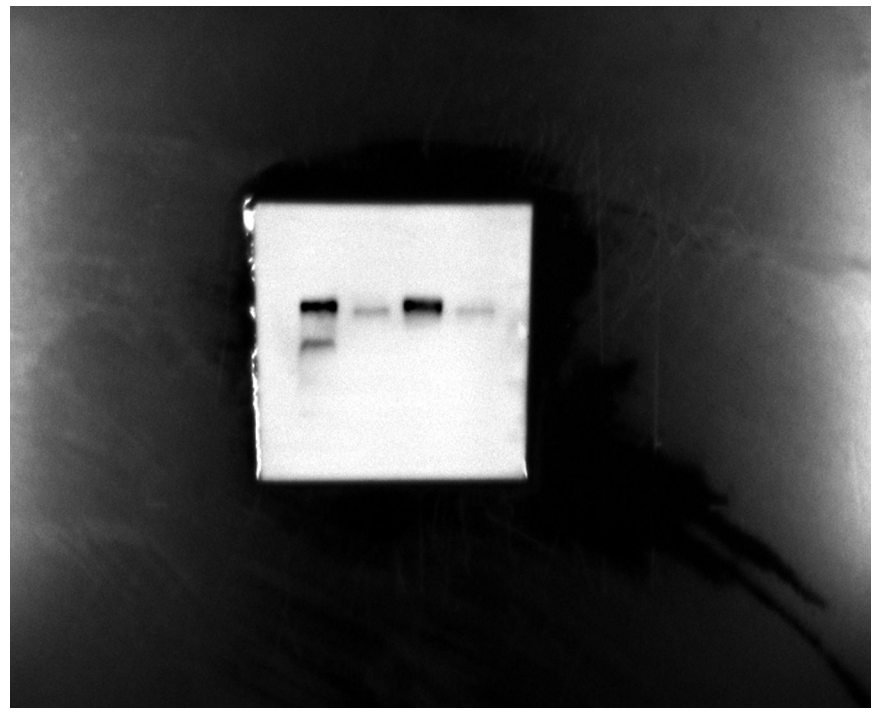

eEF1A1

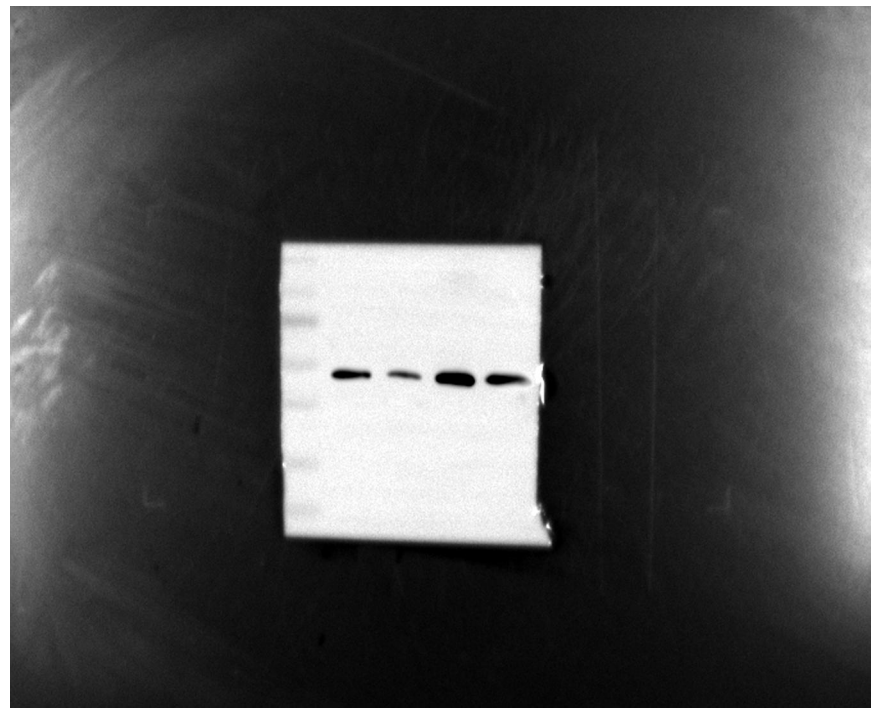

GAPDH

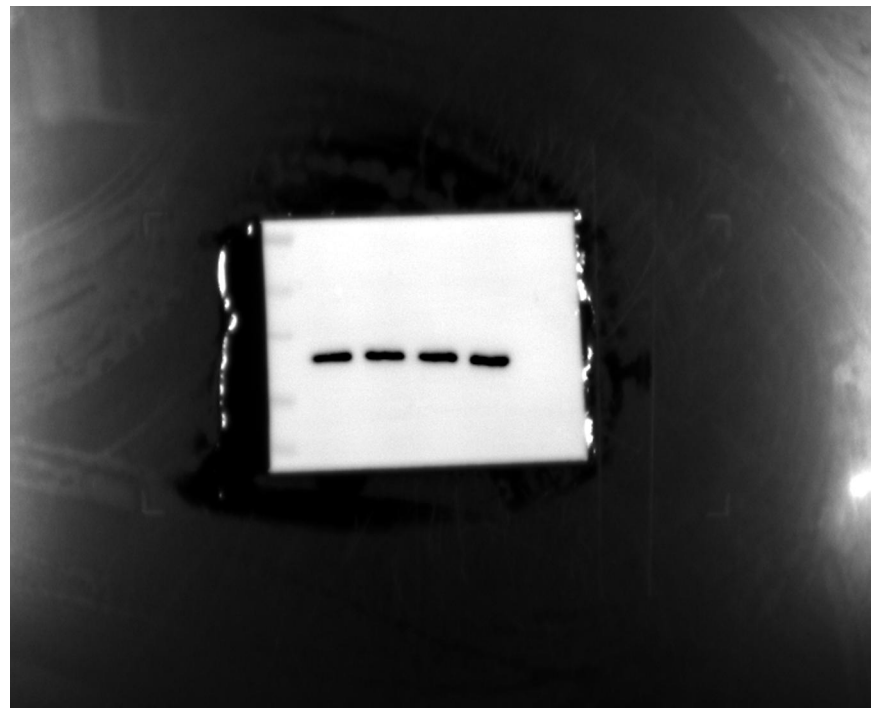

Figure 2D

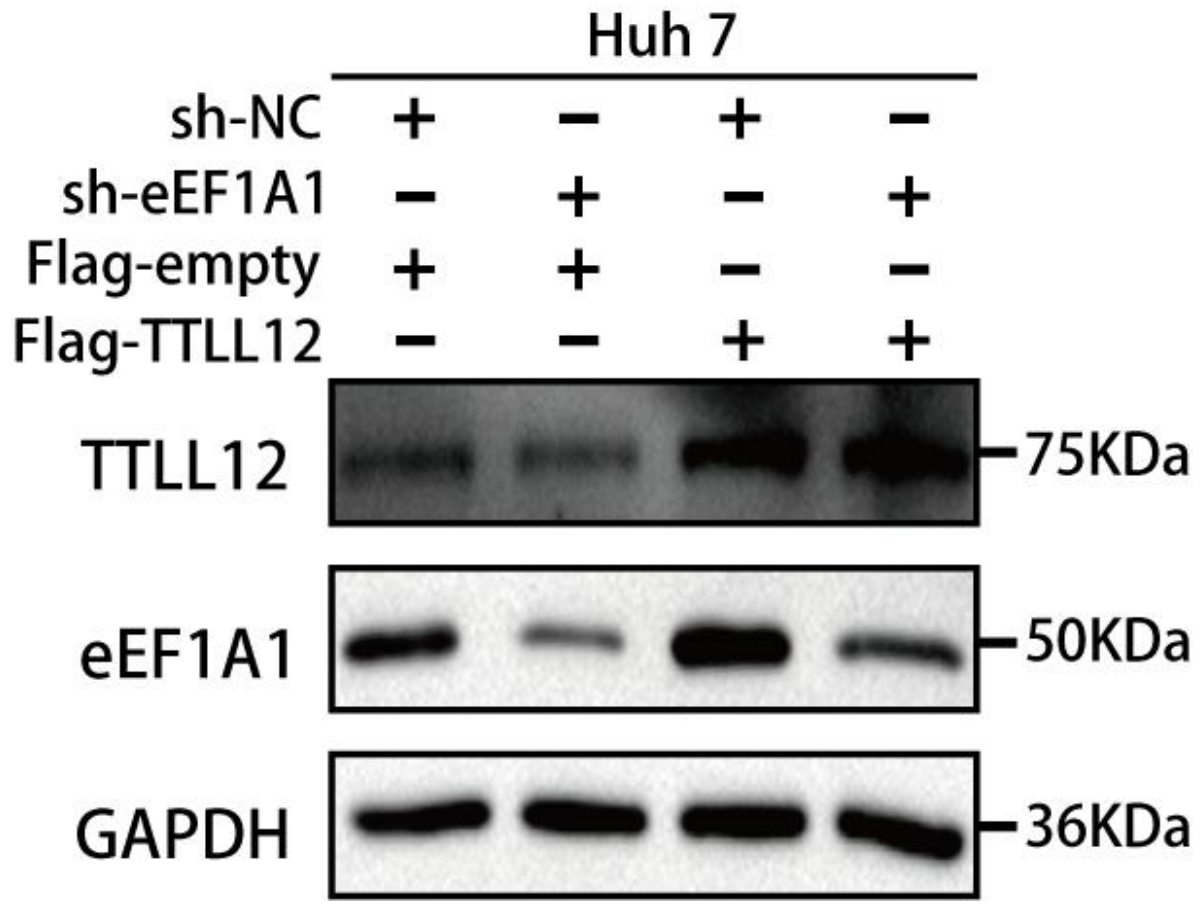

TTLL12

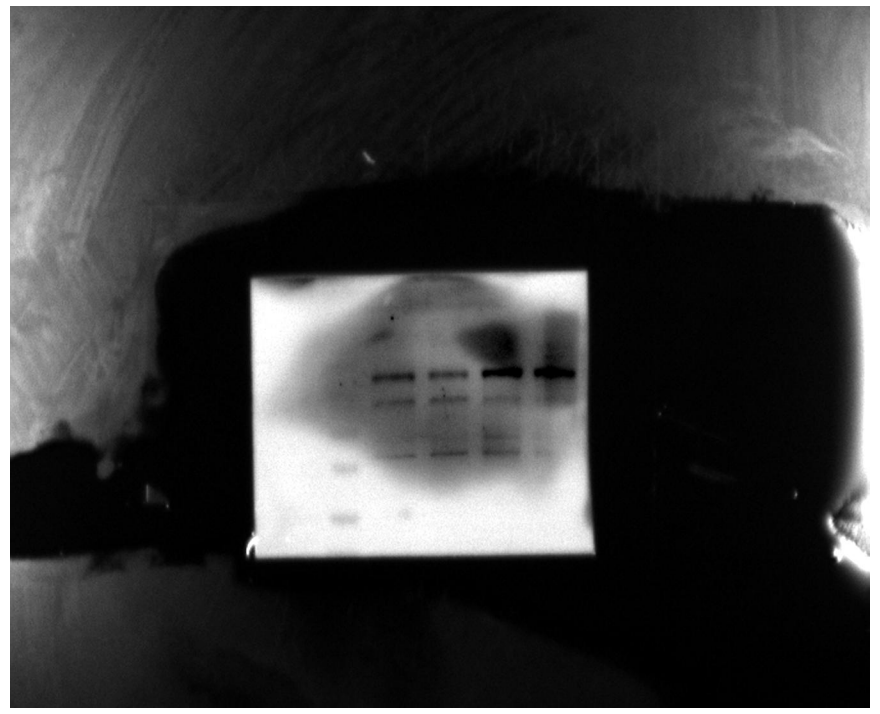

eEF1A1

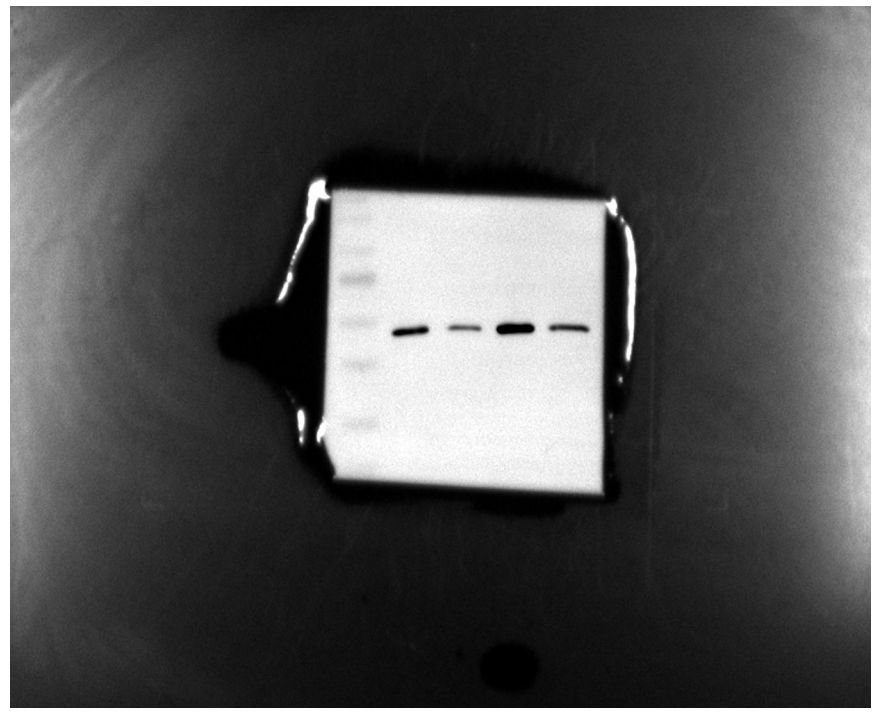

GAPDH

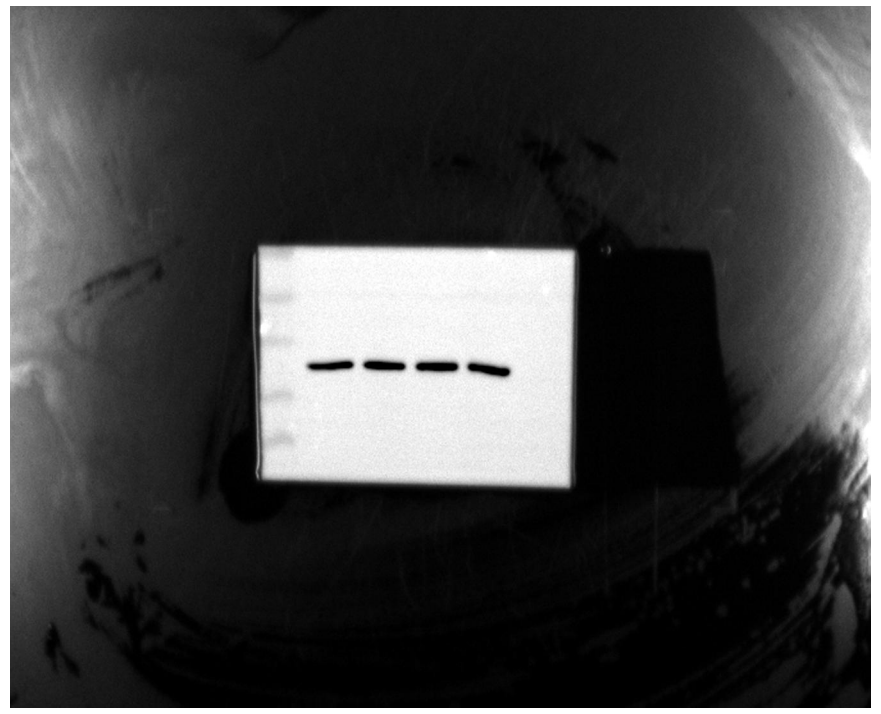

Figure 2I

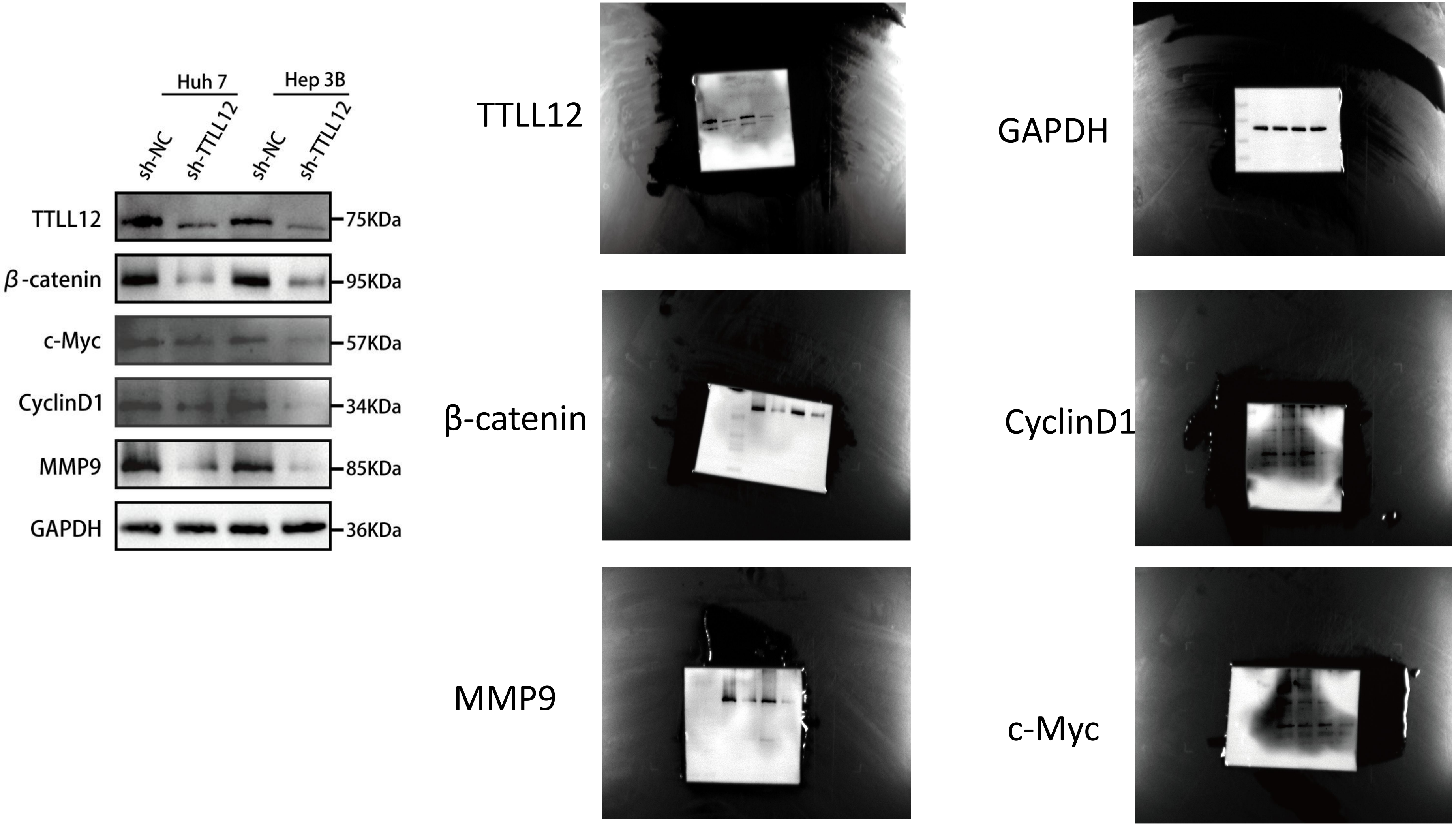

Huh 7
Hep 3B
sh-NC
sh-TTLL12
sh-NC
sh-TTLL12
TTLL12
β-catenin
c-Myc
CyclinD1
MMP9
GAPDH
75KDa
95KDa
57KDa
34KDa
85KDa
36KDa
TTLL12
GAPDH
β-catenin
CyclinD1
MMP9
c-Myc

Figure 3B

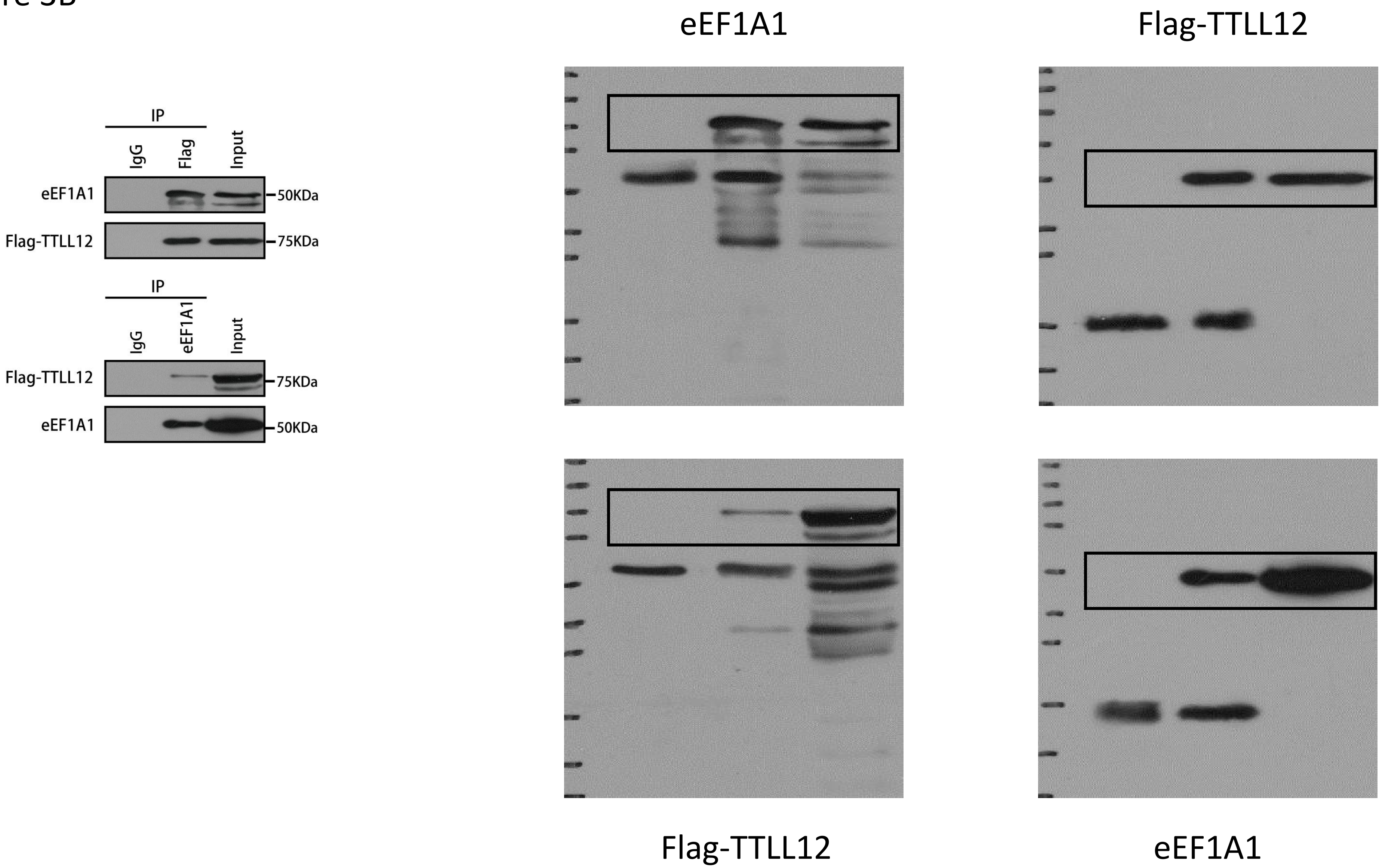
IP
IgG
Flag
Input
eEF1A1
50KDa
Flag-TTLL12
75KDa
IP
IgG
eEF1A1
Input
Flag-TTLL12
75KDa
eEF1A1
50KDa
eEF1A1
Flag-TTLL12
Flag-TTLL12
eEF1A1

Figure 3D

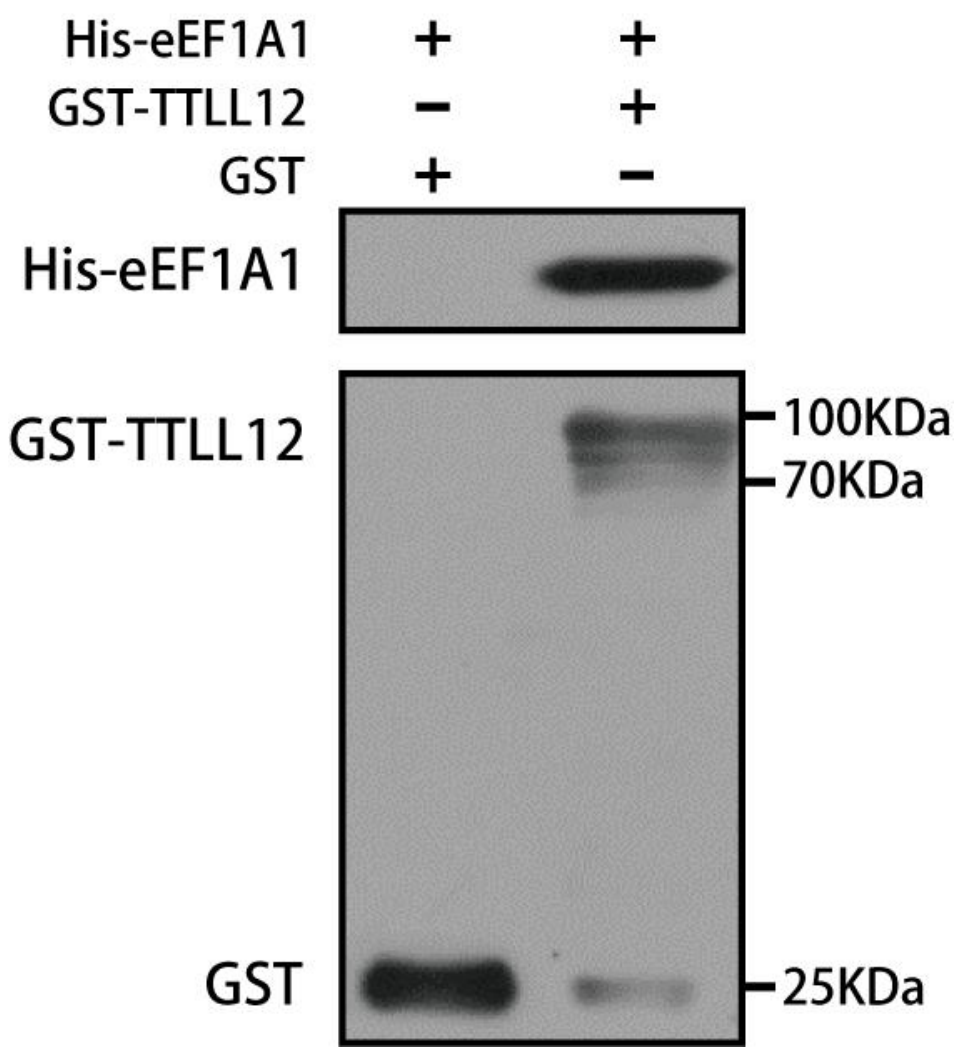

GST-TTLL12

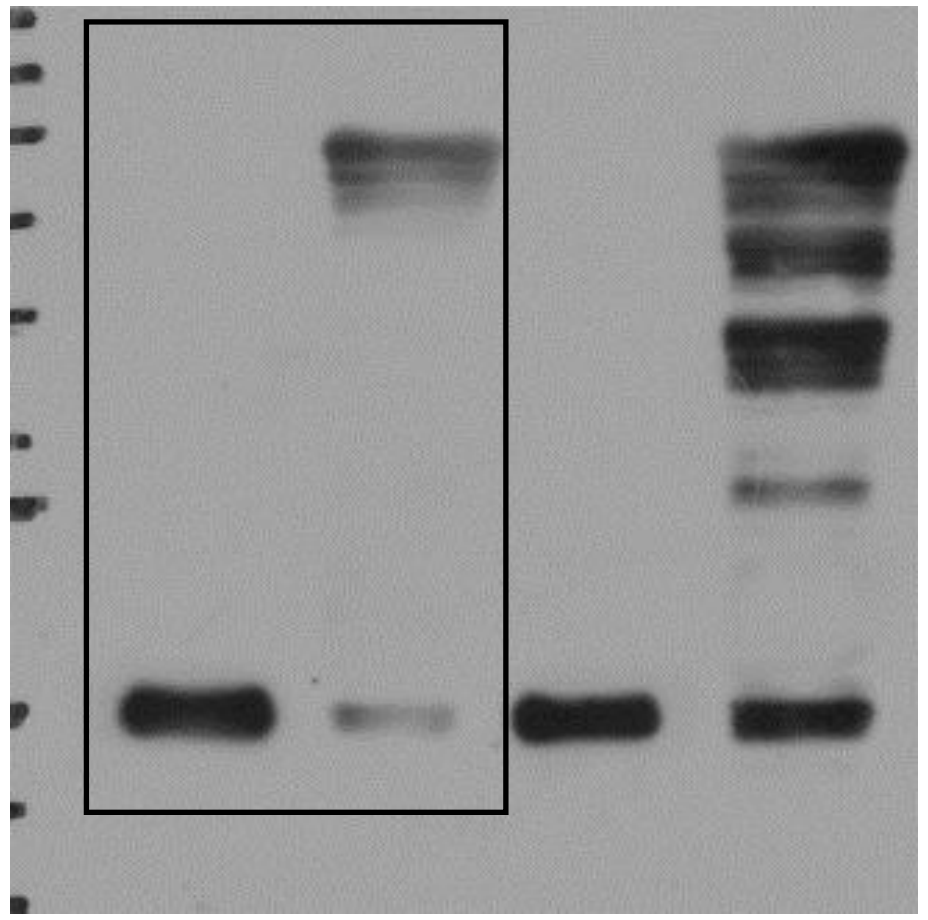

His-eEF1A1

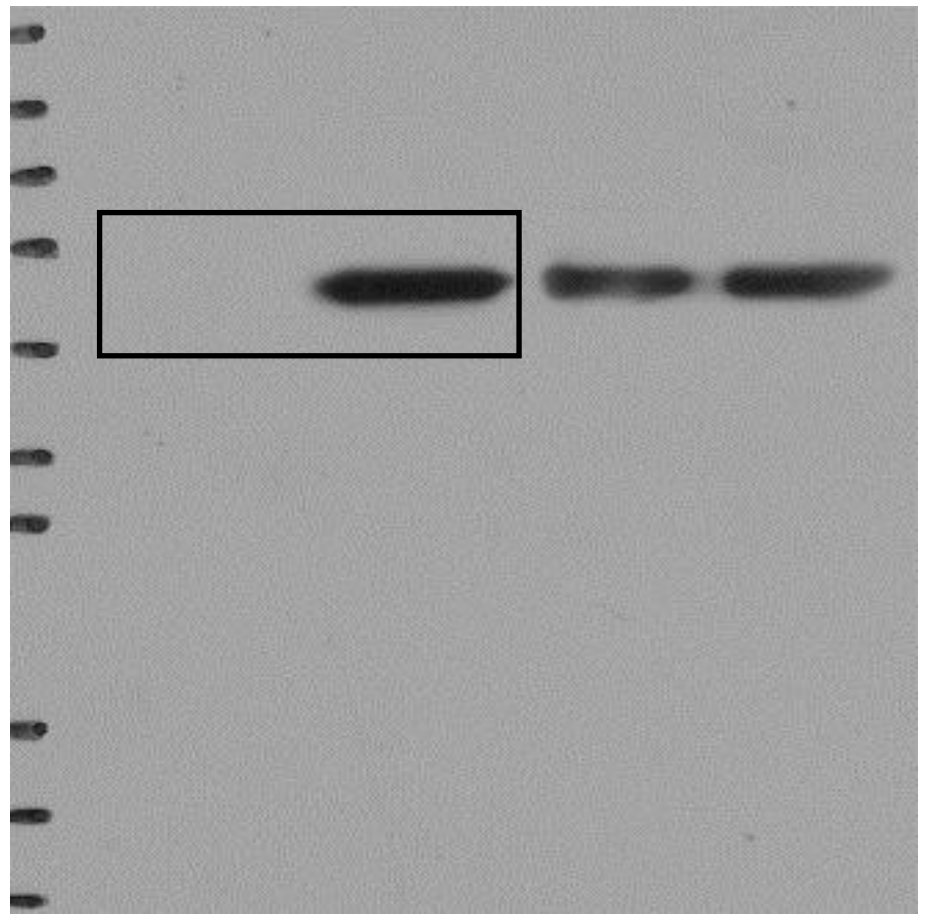

Figure 3E

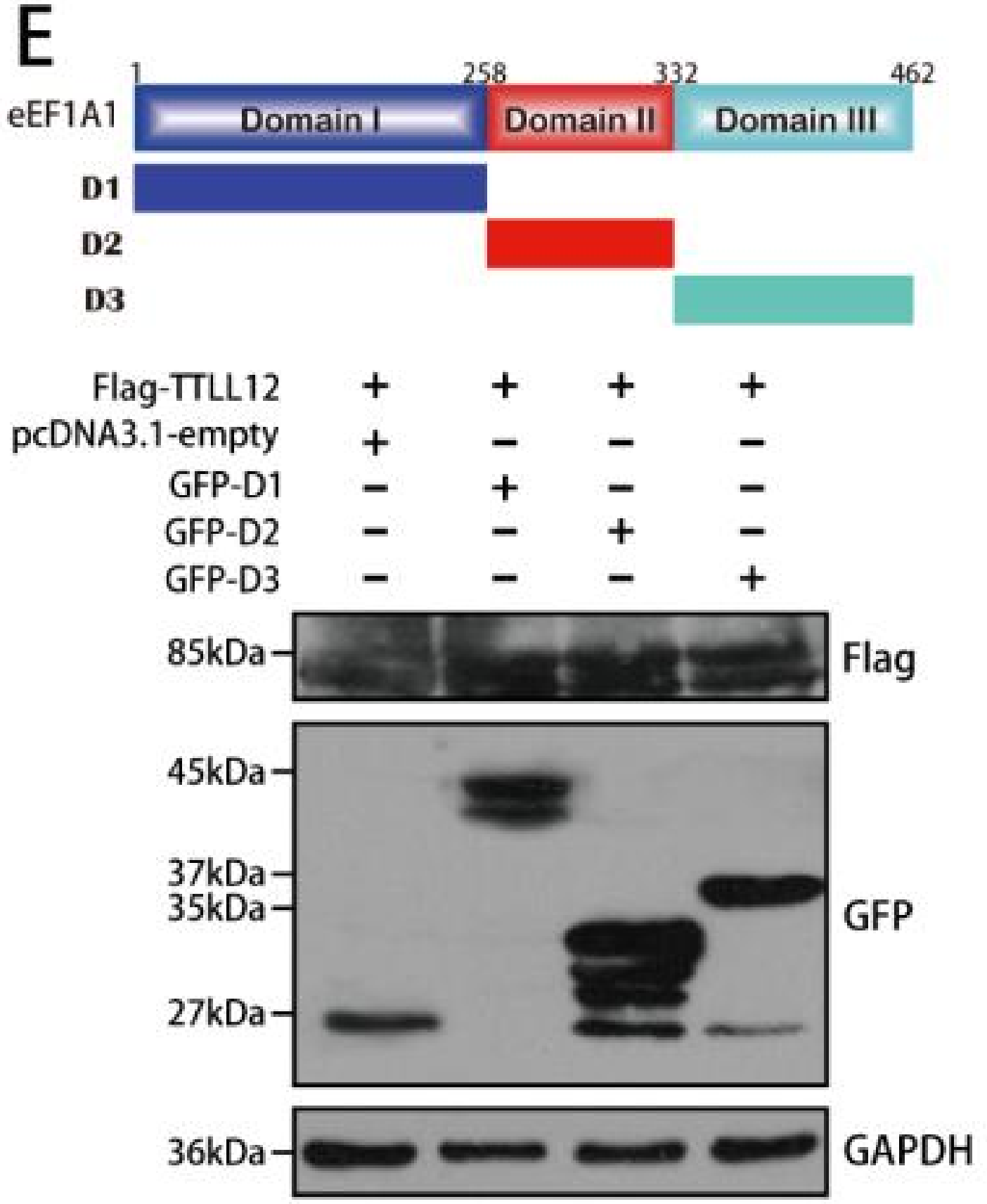

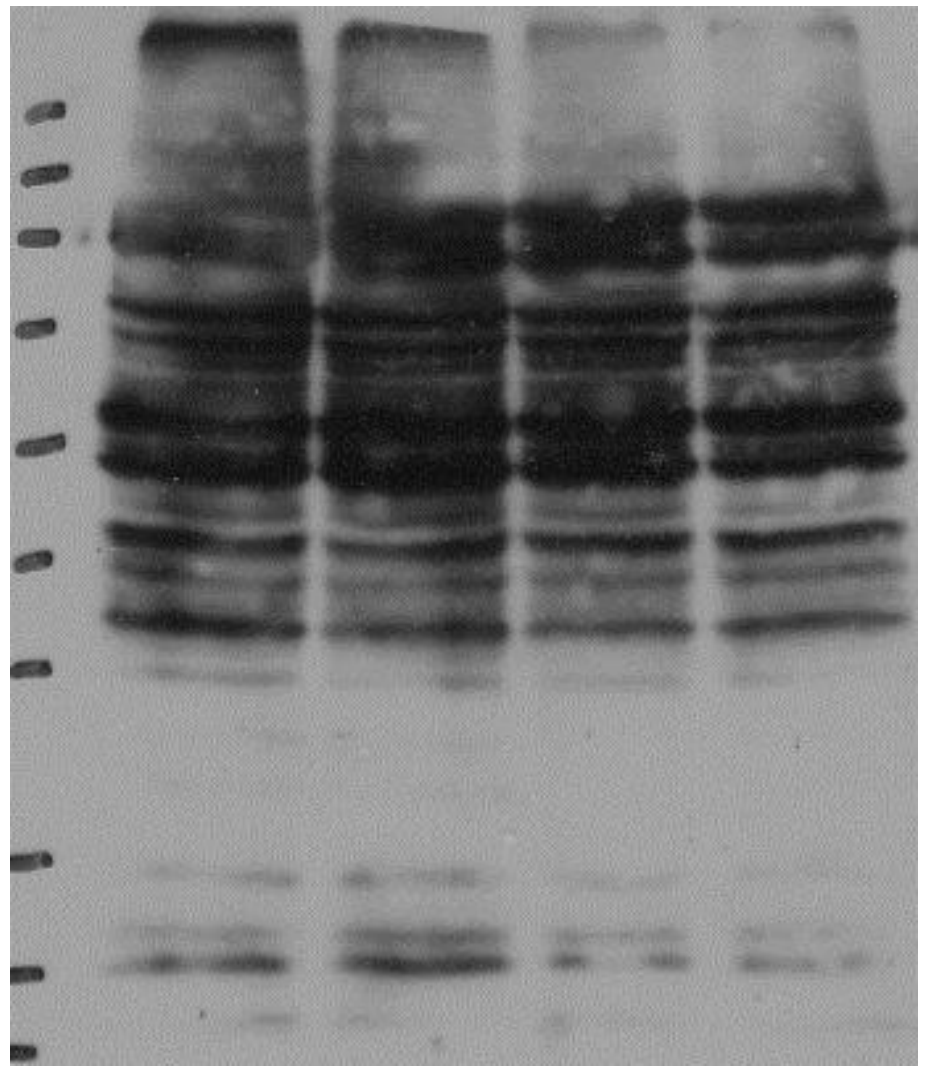

Flag-TTLL12

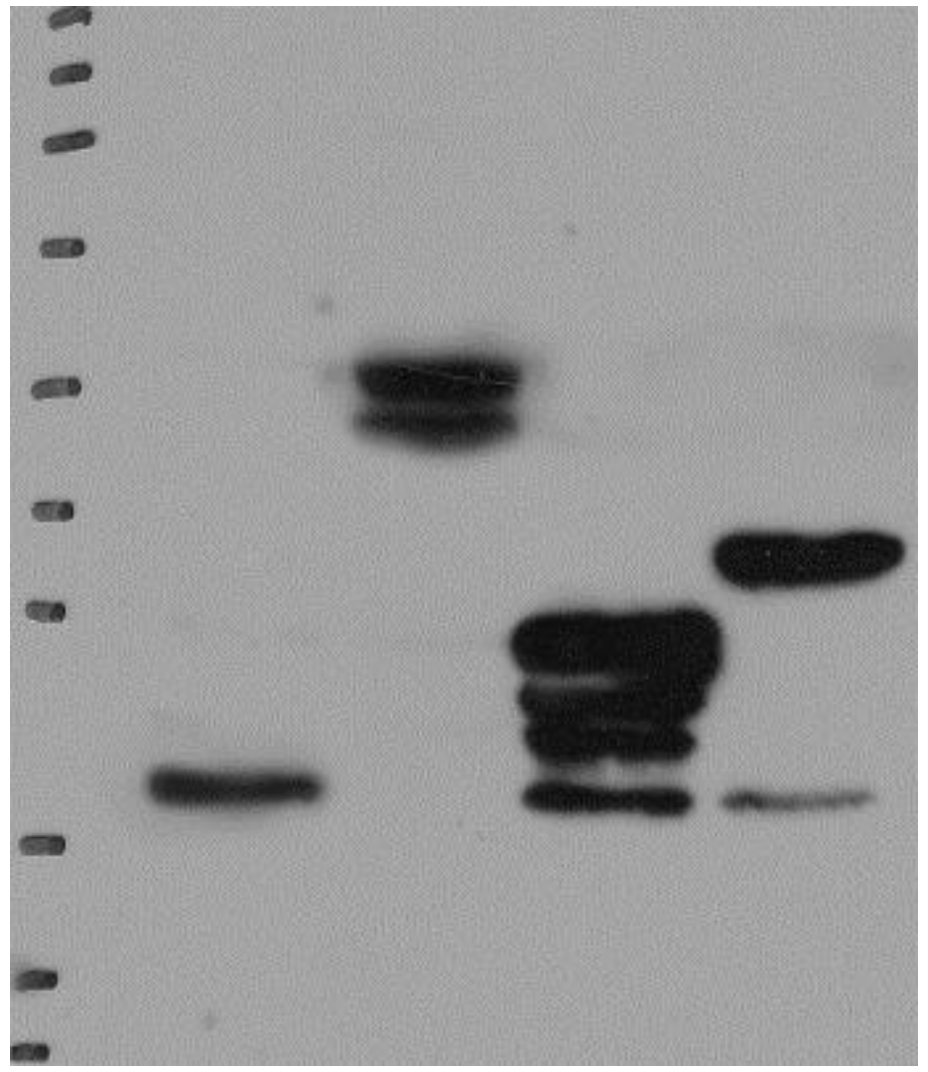

GFP

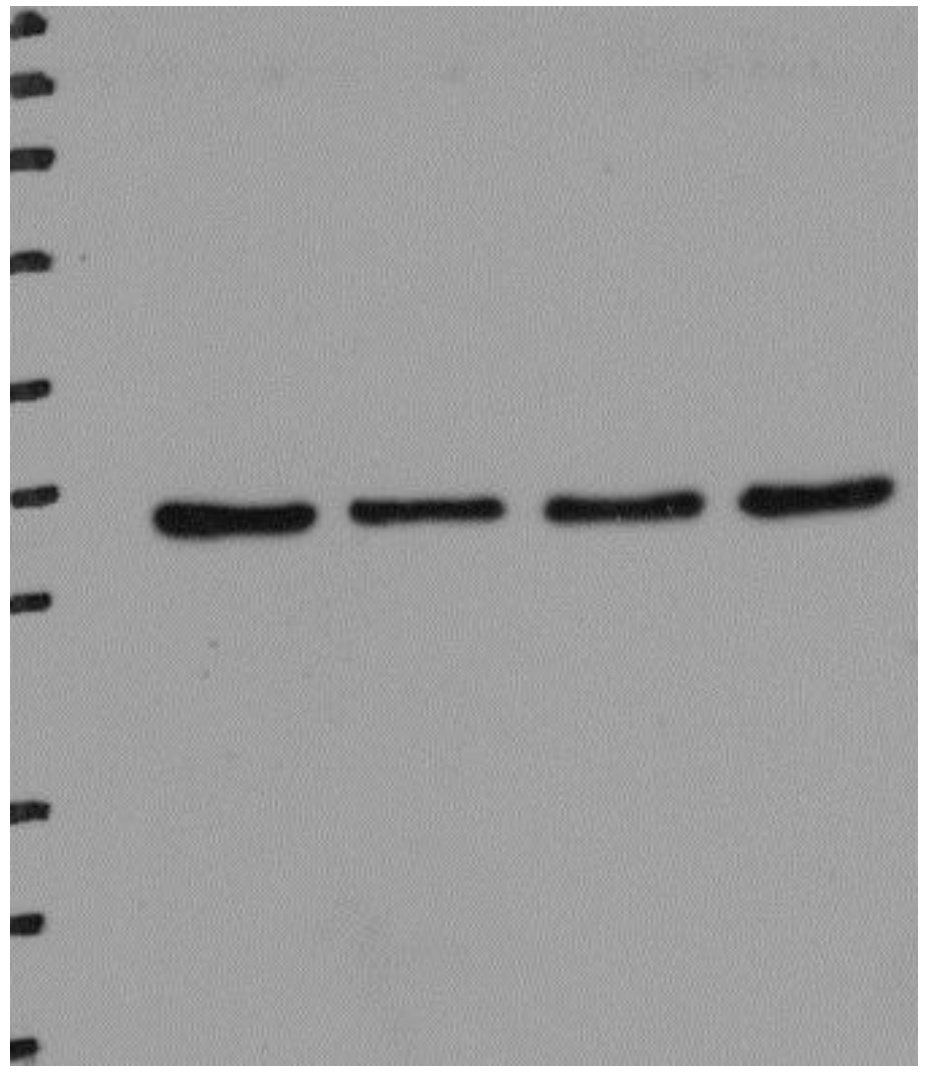

GAPDH

Figure 3J

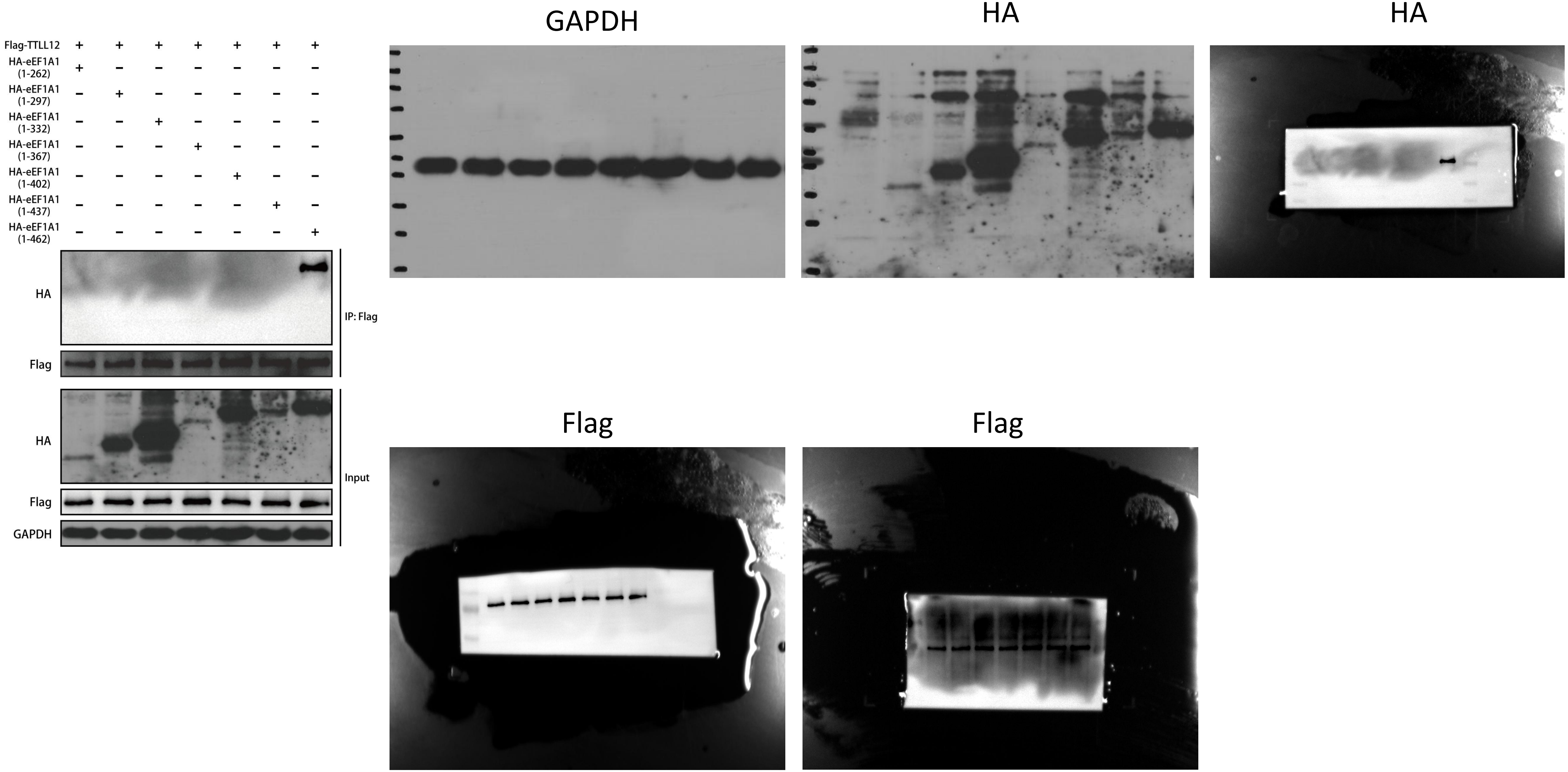

Flag-TTLL12
HA-eEF1A1 (1-262)
HA-eEF1A1 (1-297)
HA-eEF1A1 (1-332)
HA-eEF1A1 (1-367)
HA-eEF1A1 (1-402)
HA-eEF1A1 (1-437)
HA-eEF1A1 (1-462)
HA
Flag
IP: Flag
HA
Flag
GAPDH
Input
GAPDH
HA
HA
Flag
Flag

Figure 3K

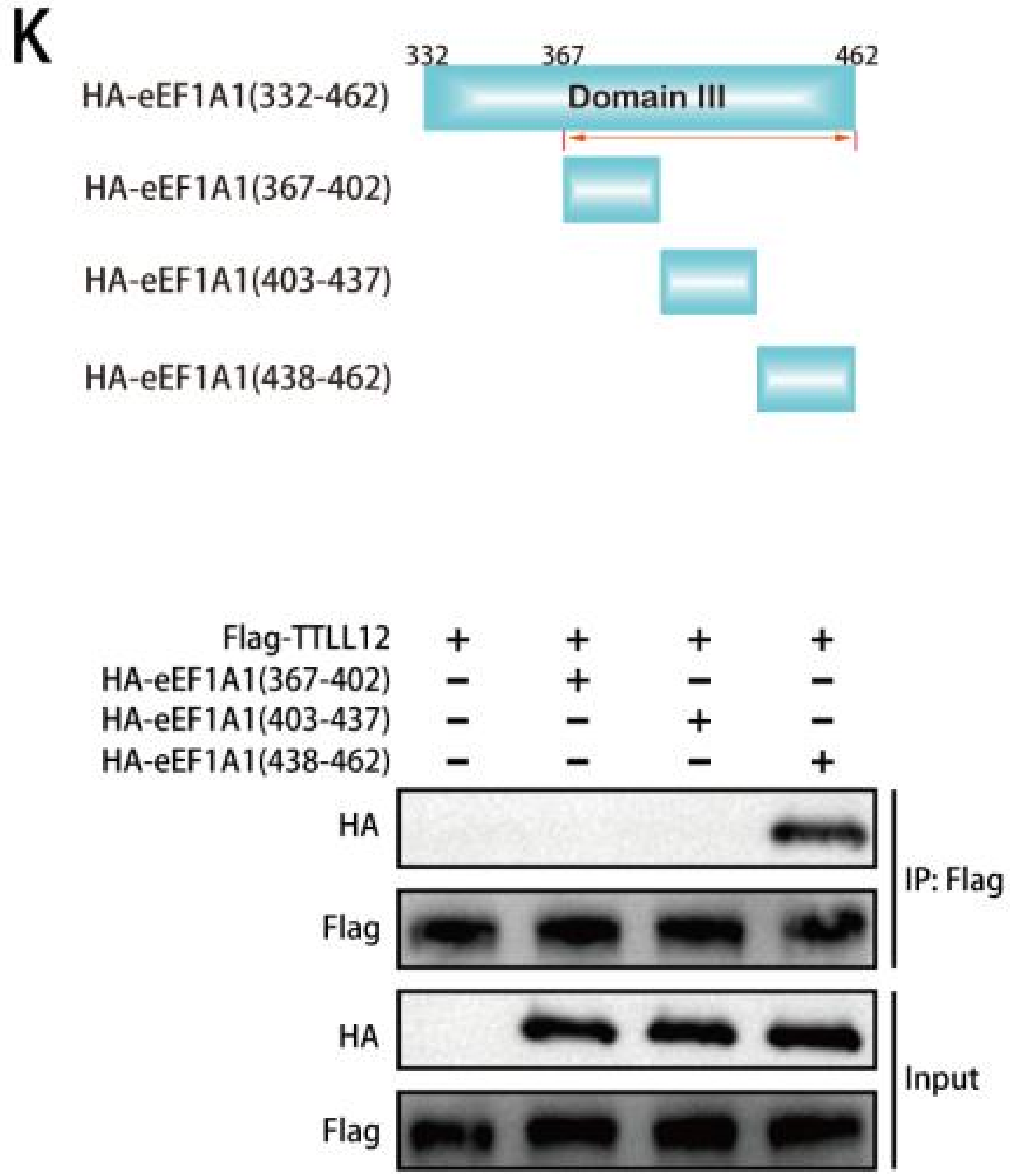

Flag

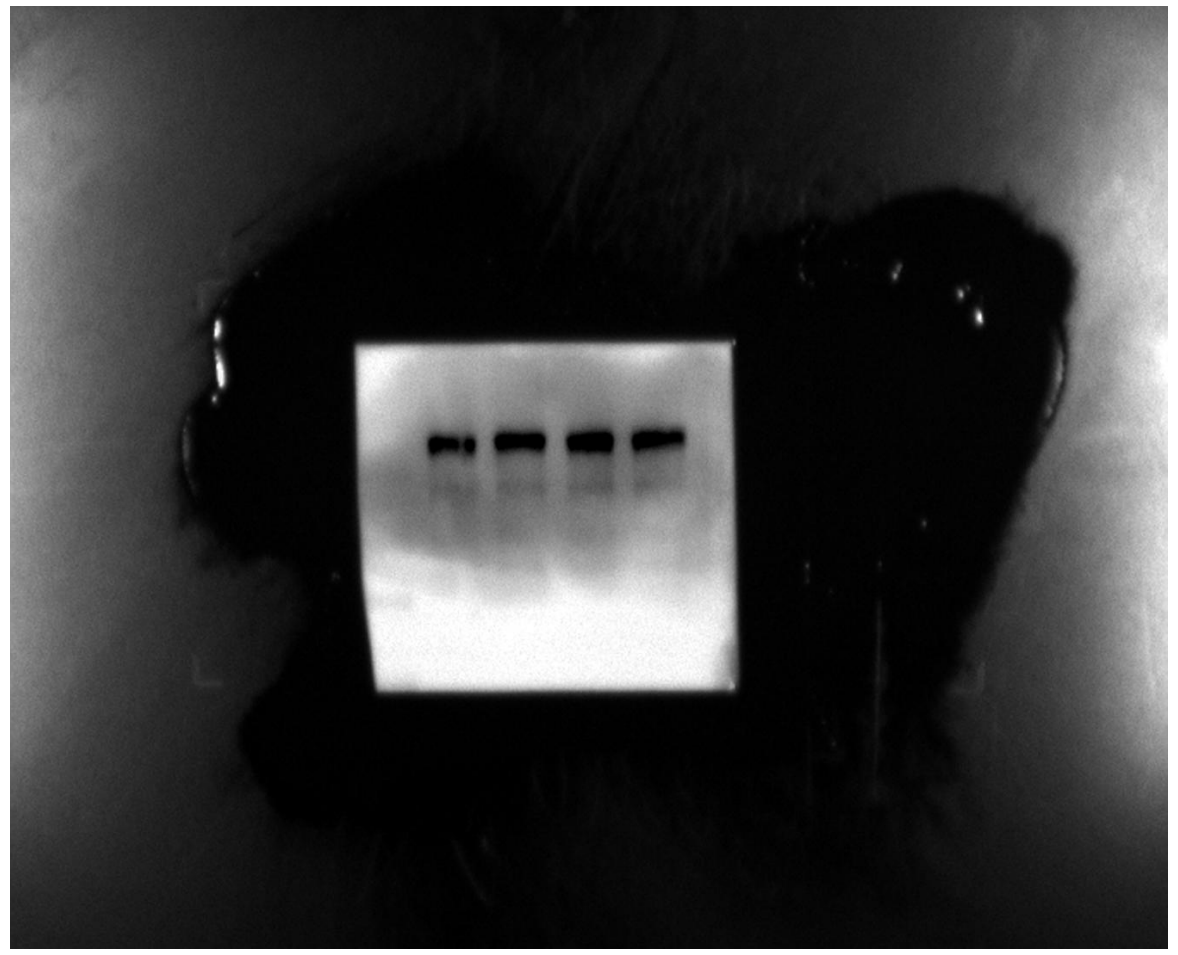

Flag

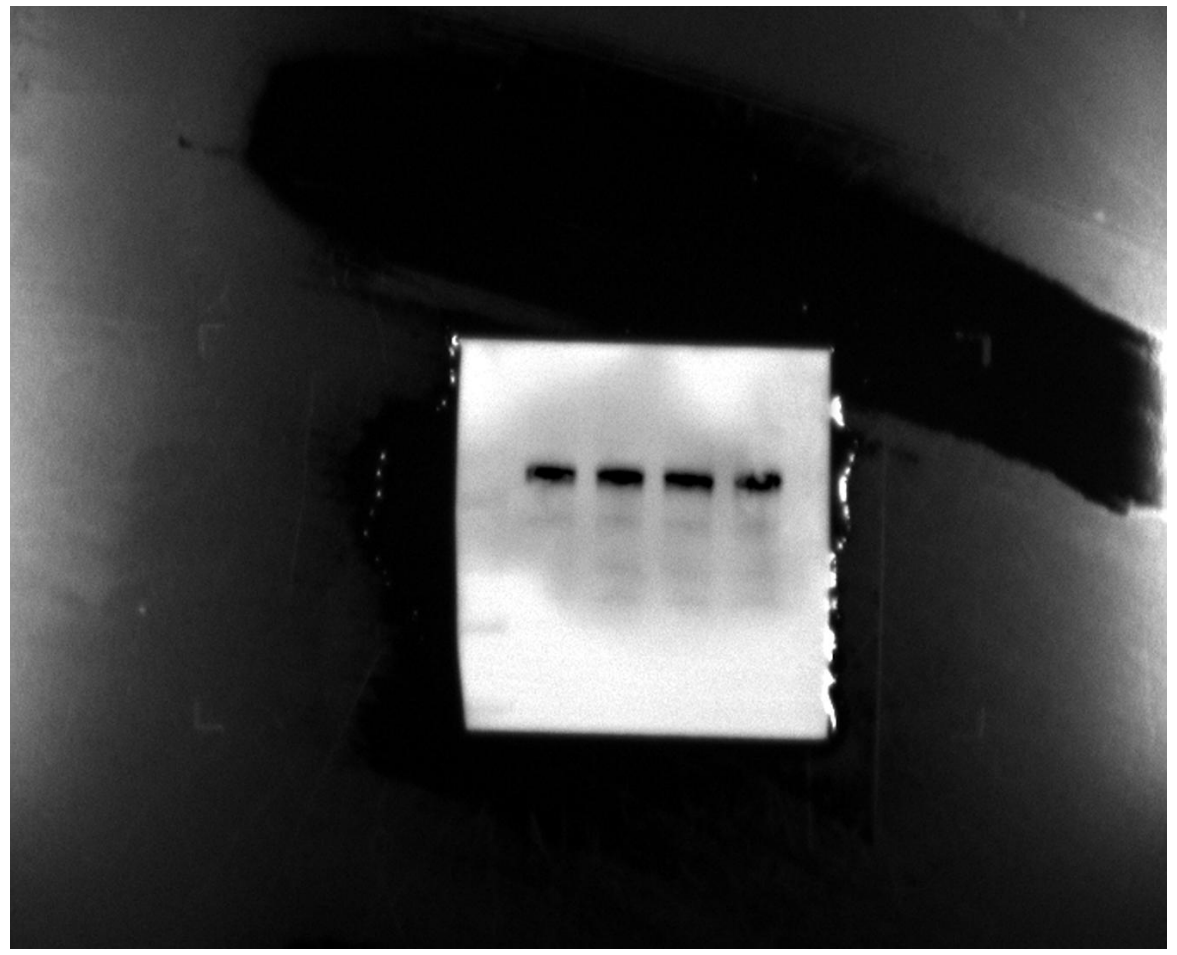

HA

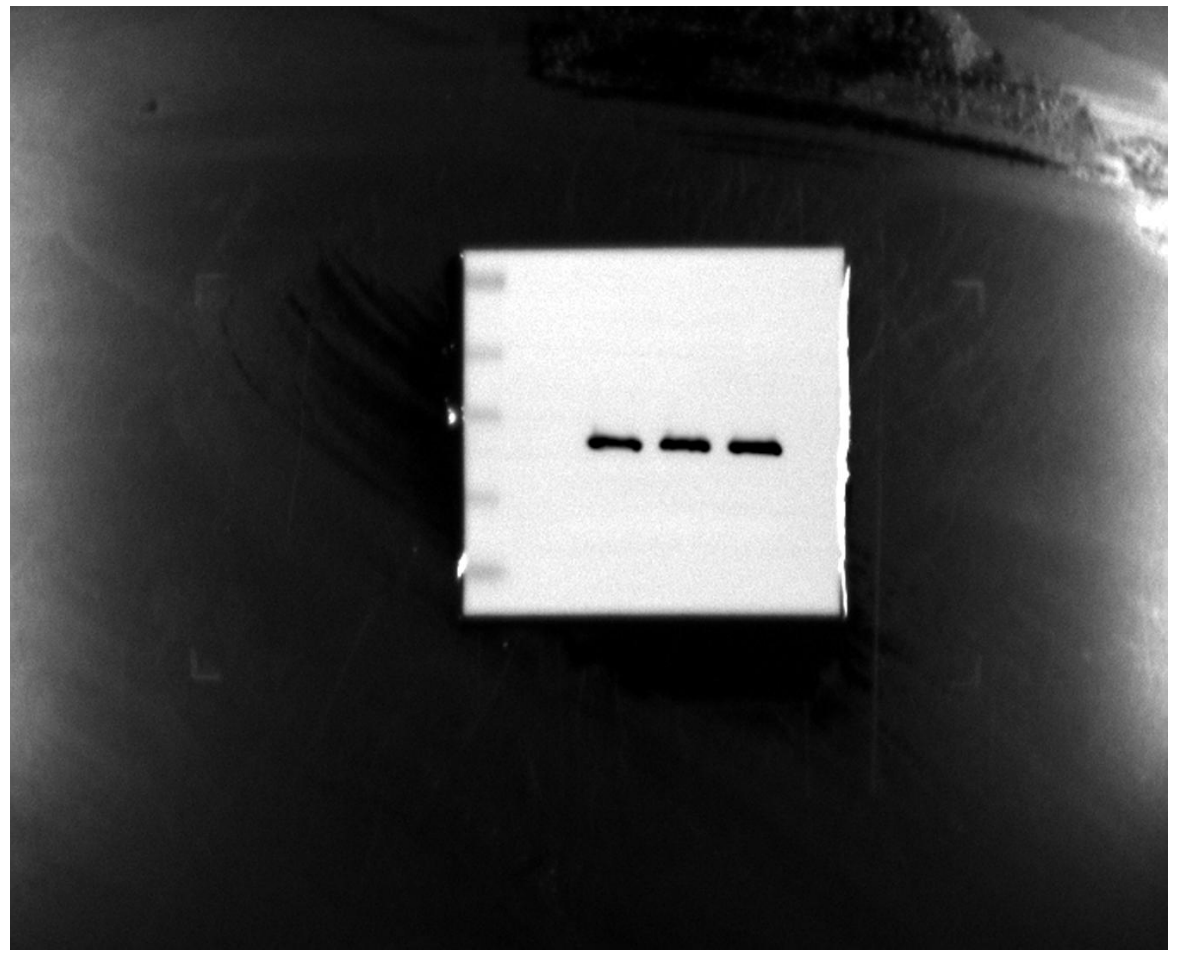

HA

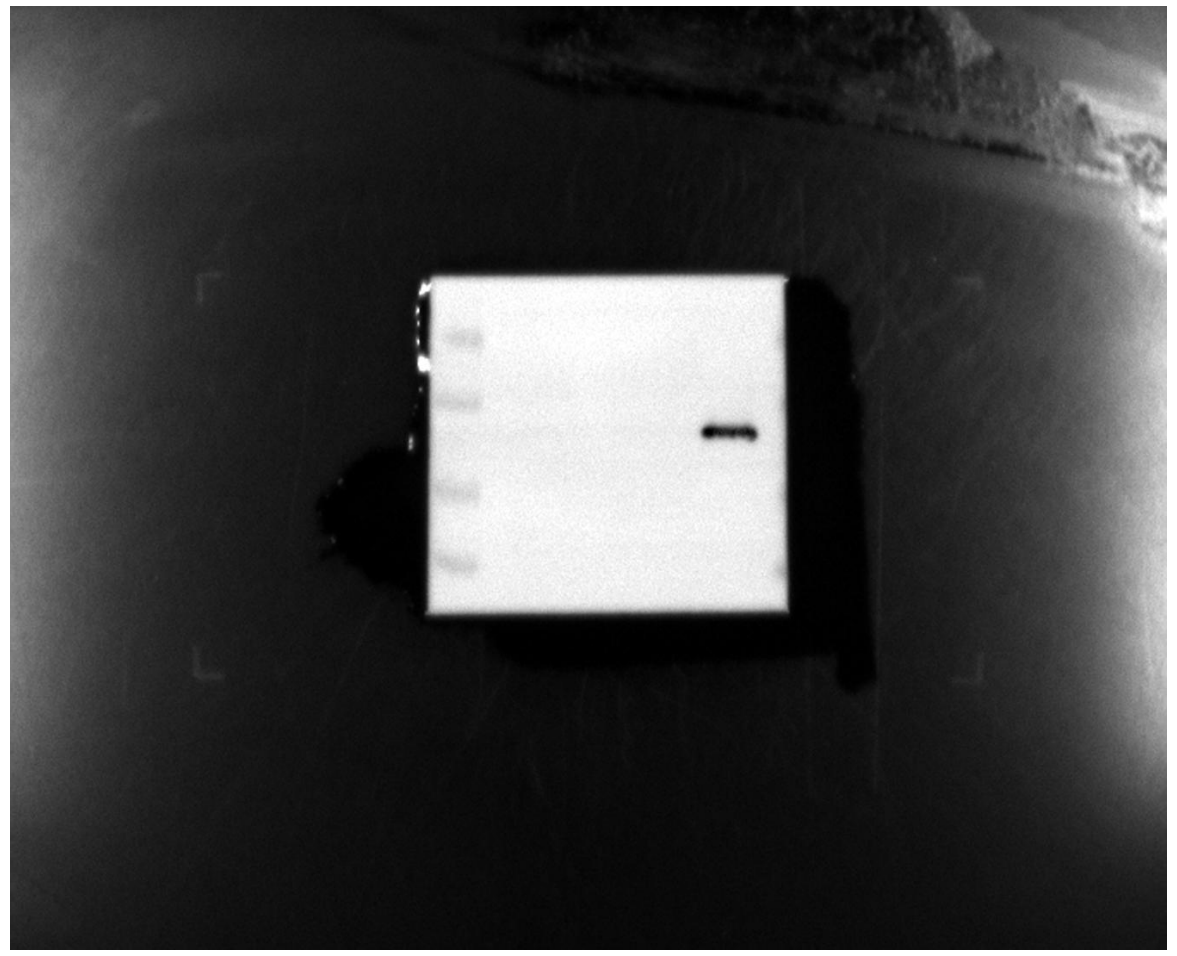

Figure 4A

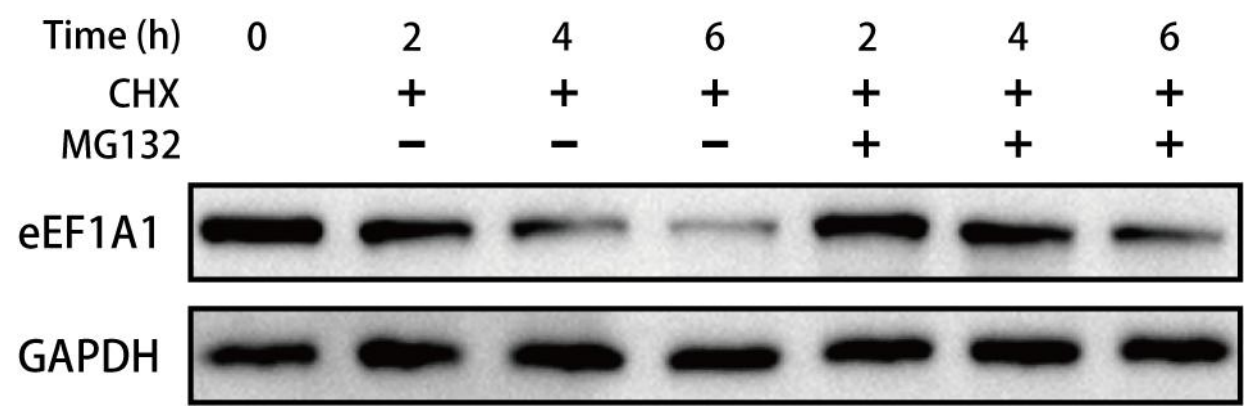

eEF1A1

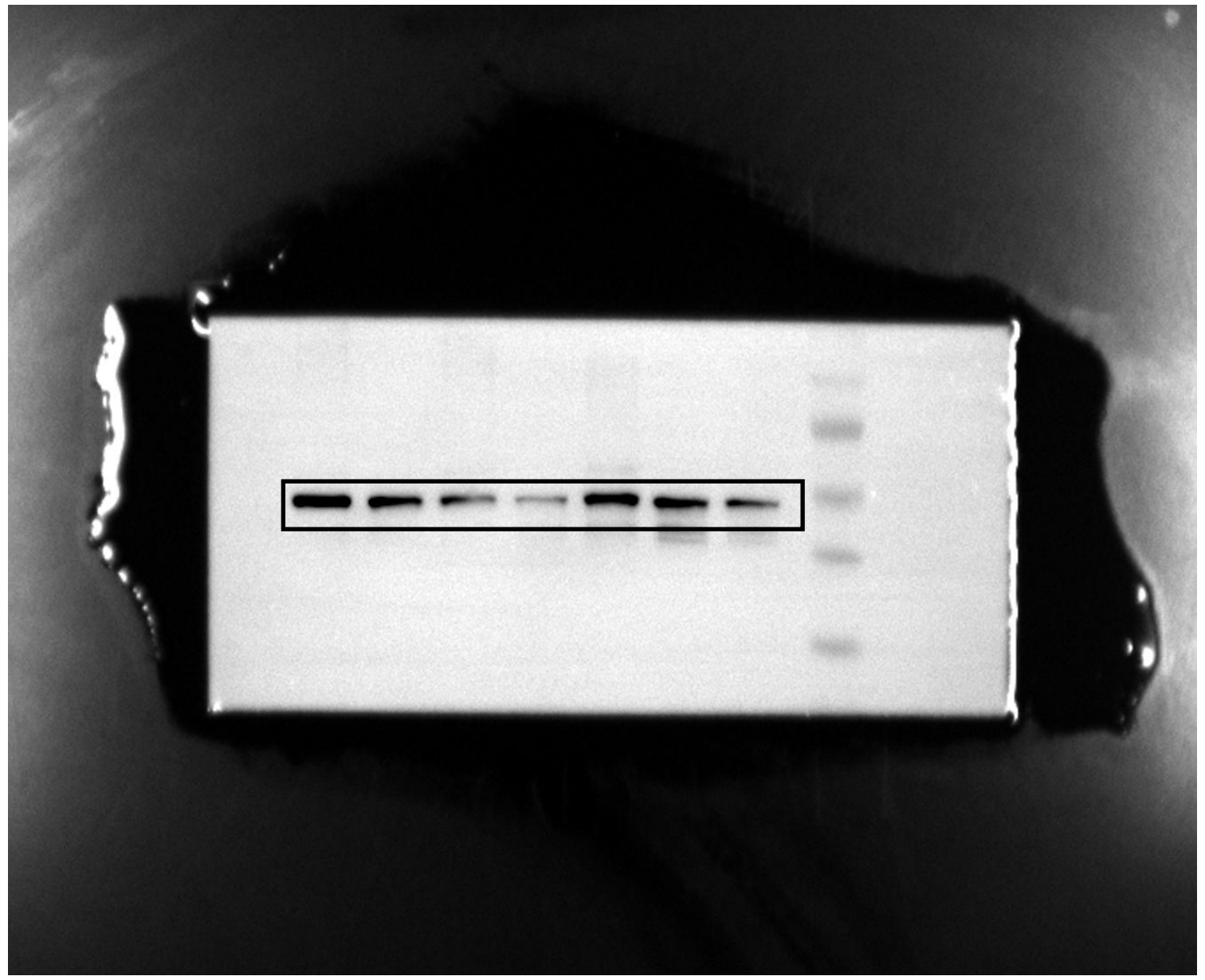

GAPDH

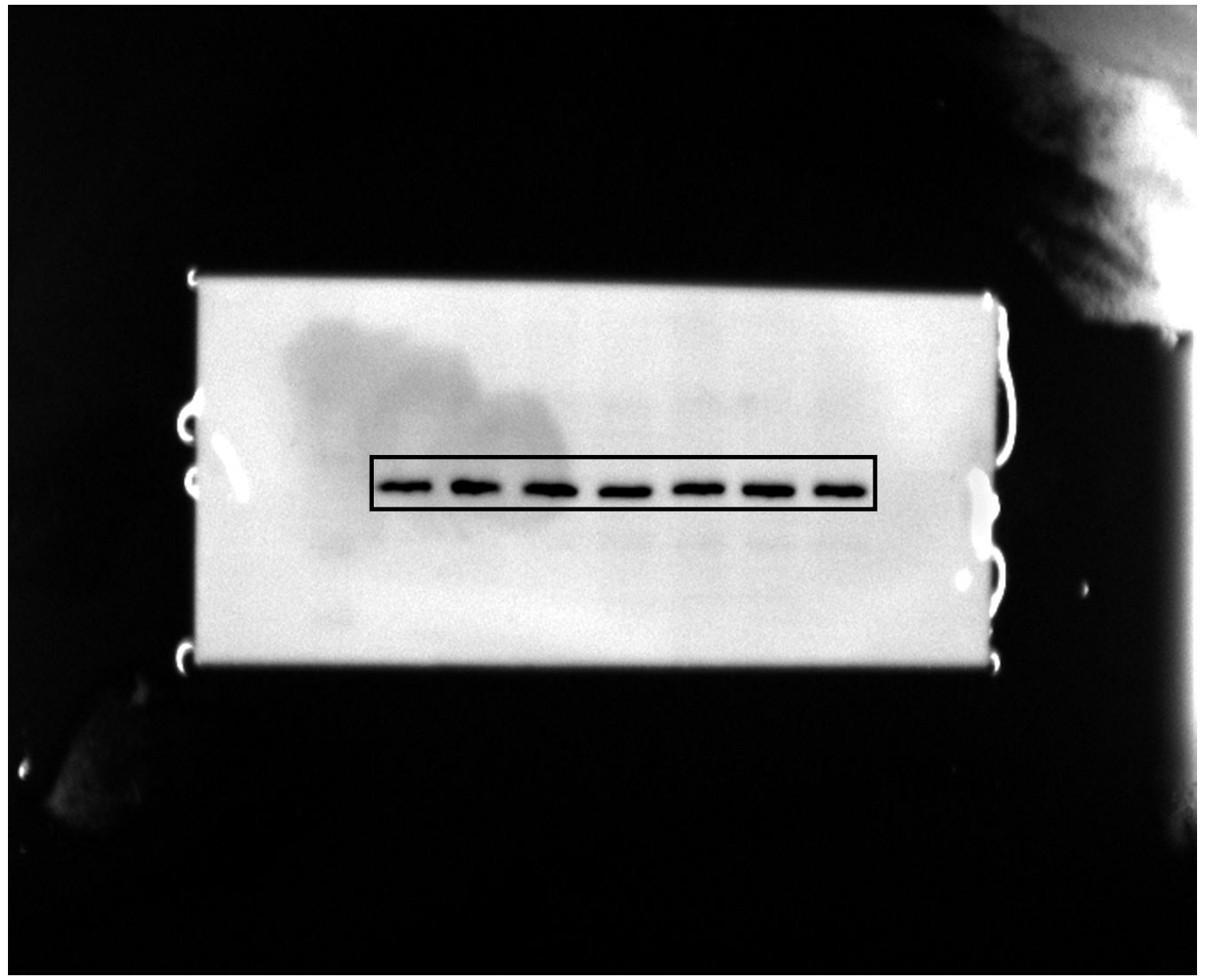

Figure 4B、 C

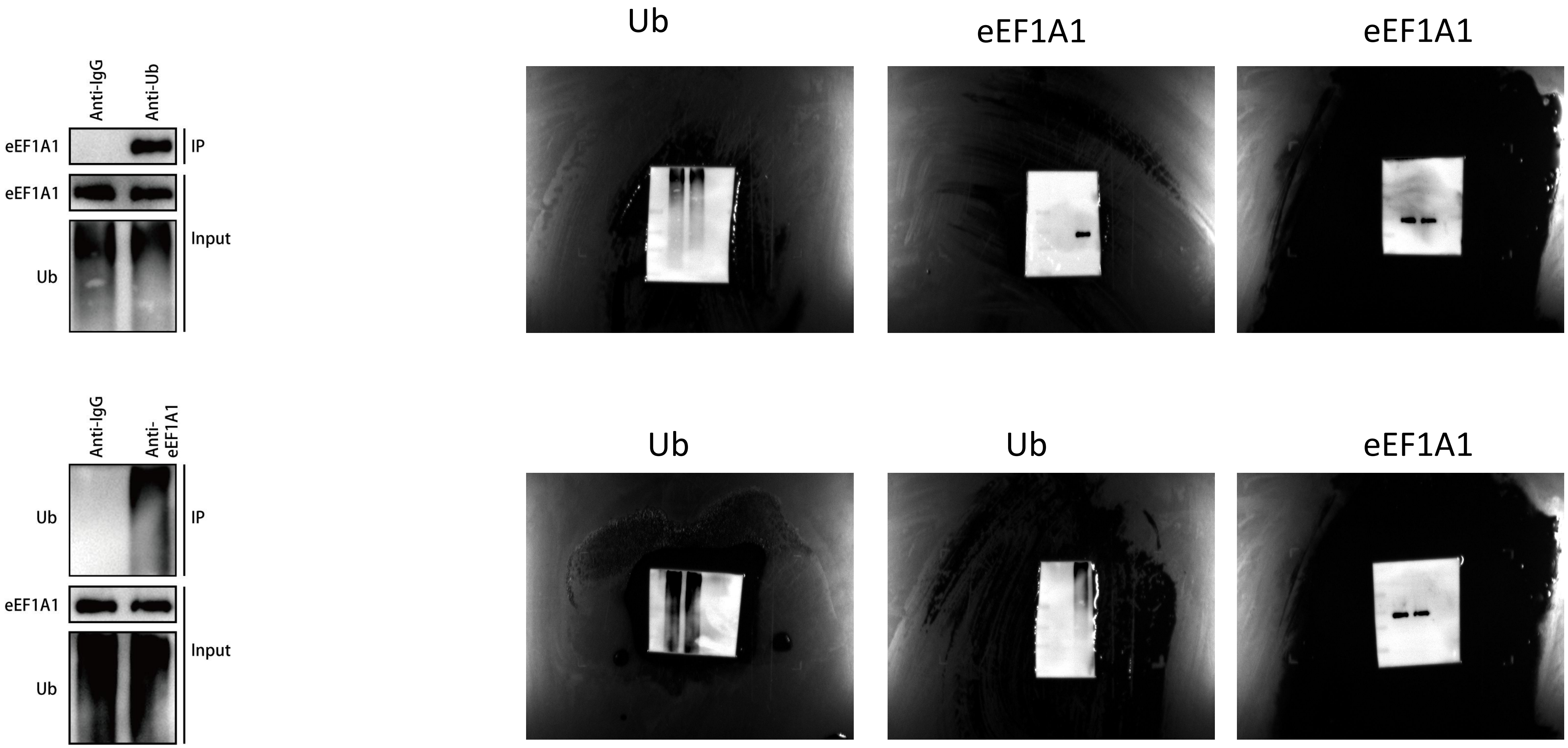

Anti-IgG
Anti-Ub
eEF1A1
IP
eEF1A1
Input
Ub
Anti-IgG
Anti-eEF1A1
Ub
IP
eEF1A1
Input
Ub
Ub
eEF1A1
eEF1A1
Ub
Ub
eEF1A1

Figure 4G

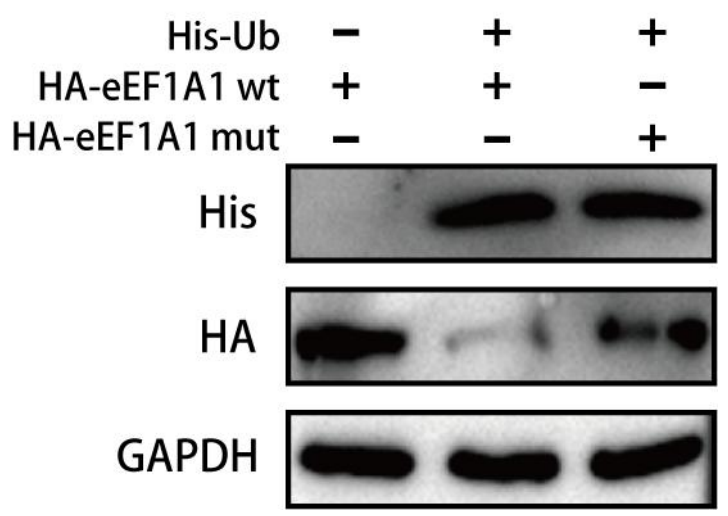

His

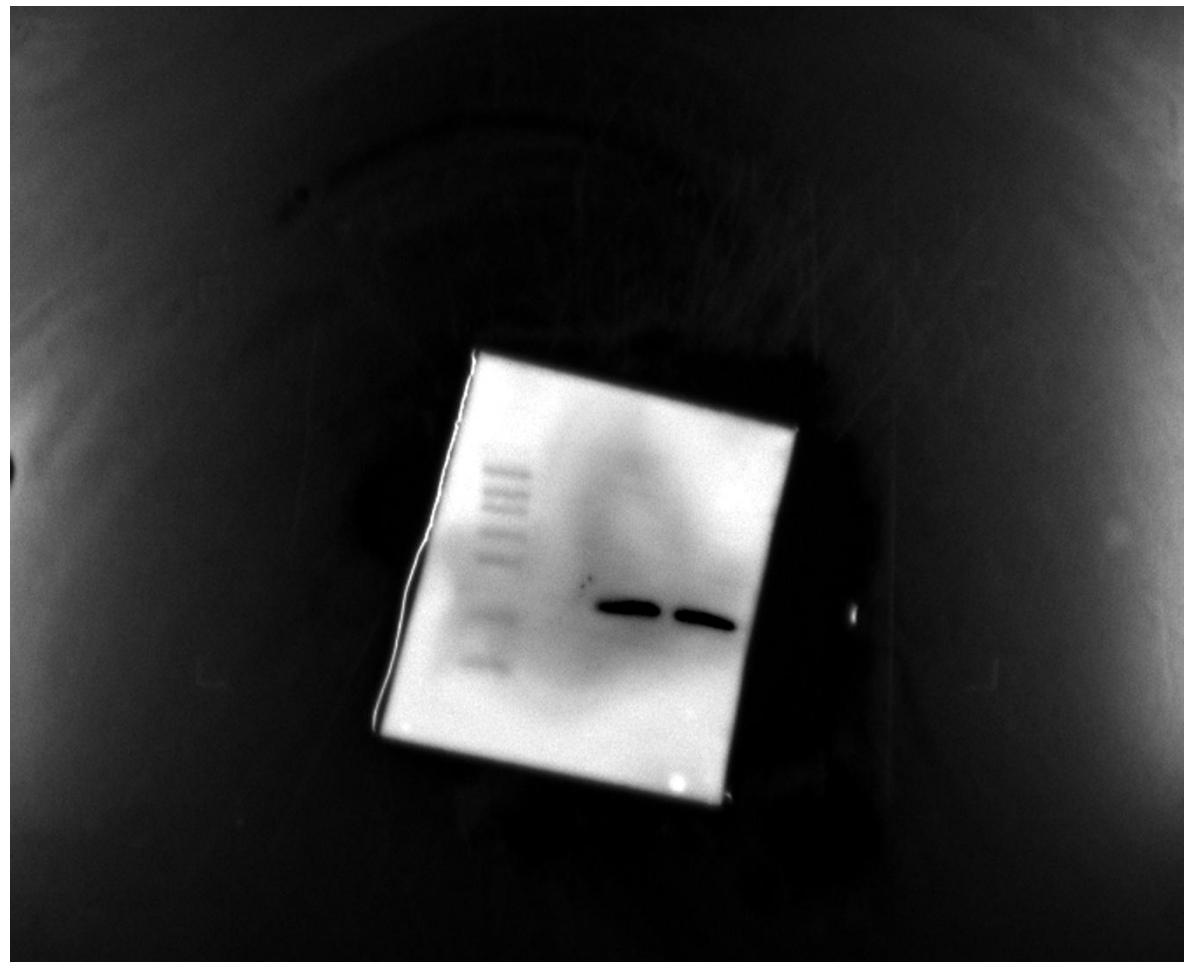

HA

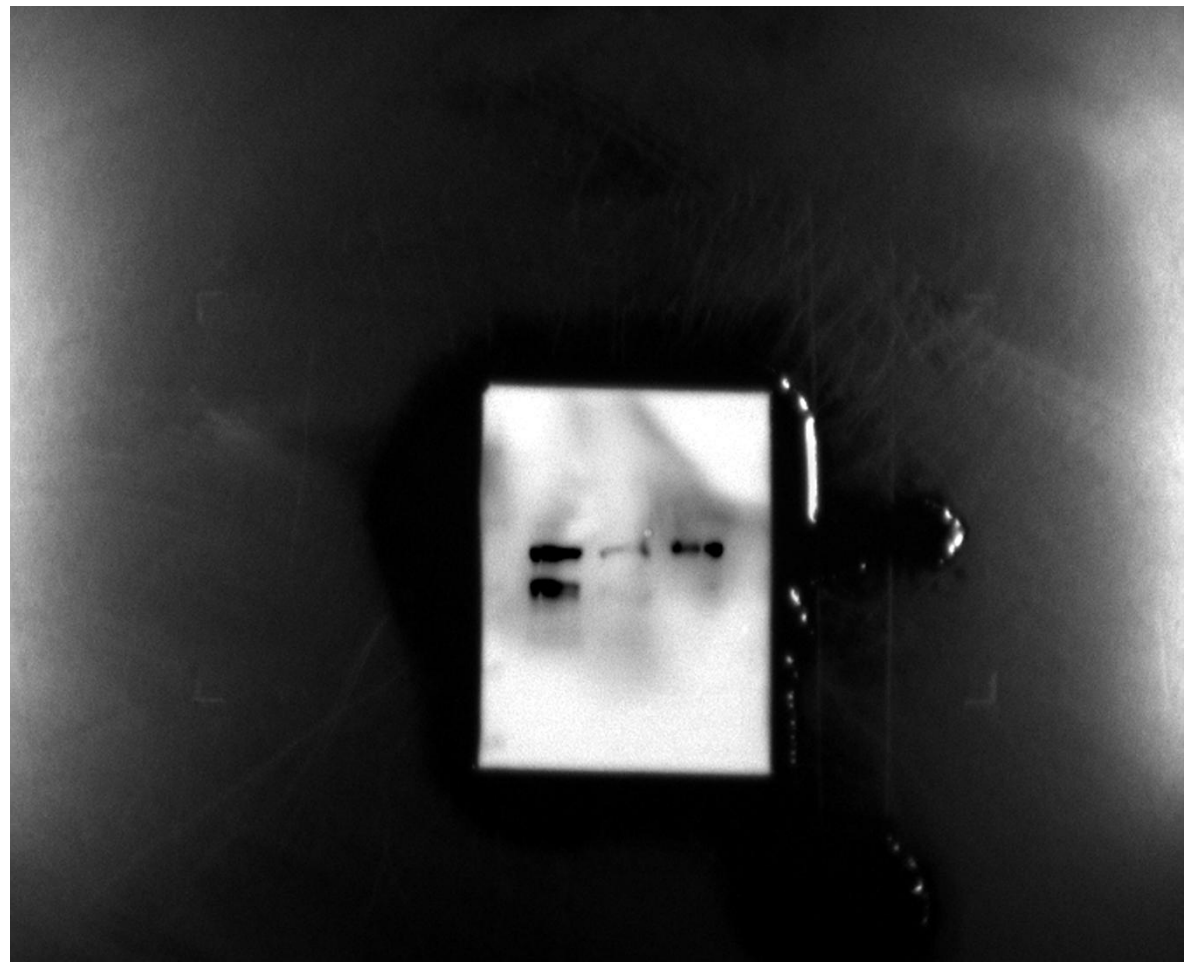

GAPDH

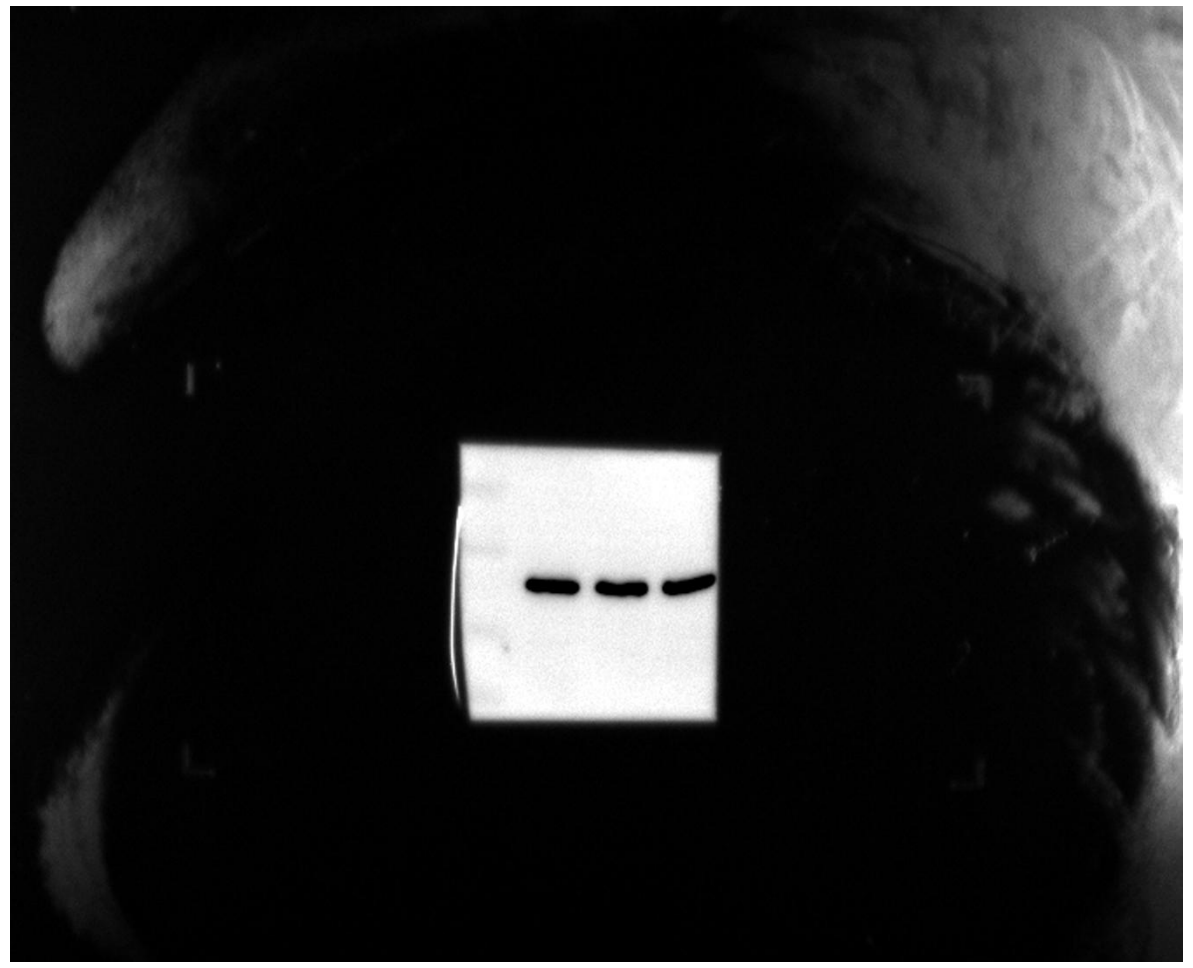

Figure 4I

I

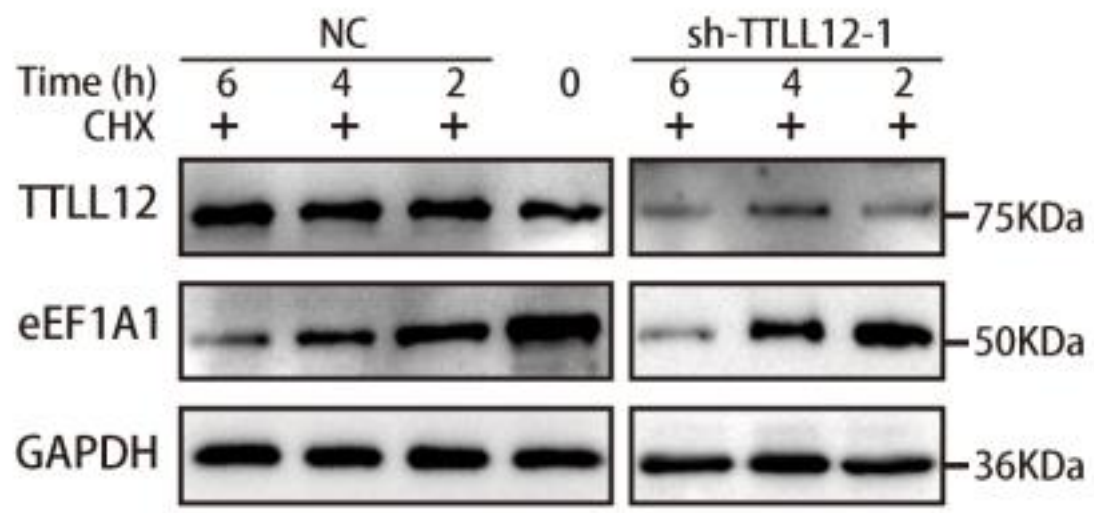

GAPDH

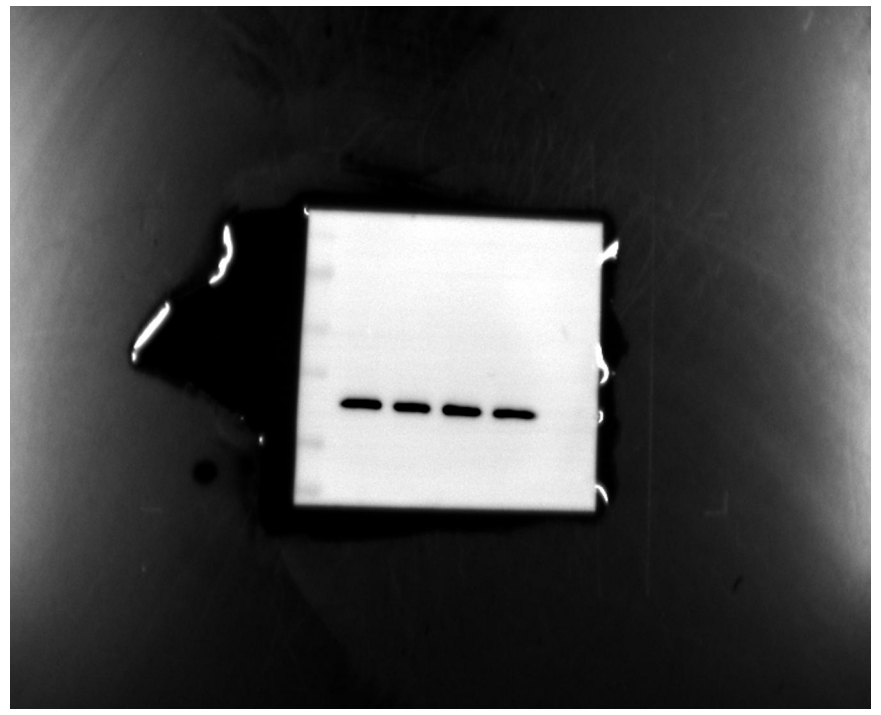

GAPDH

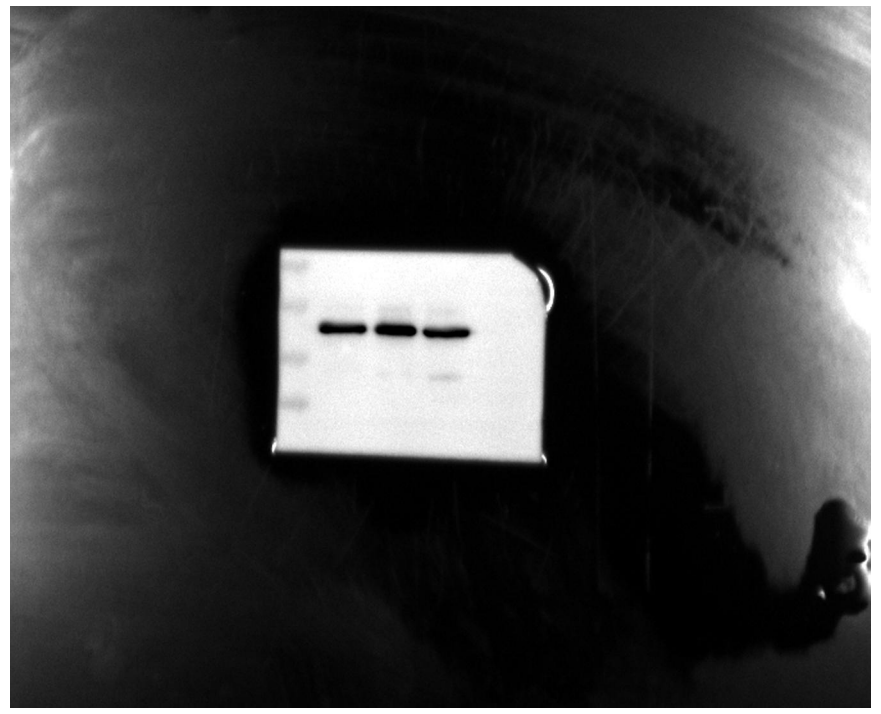

eEF1A1

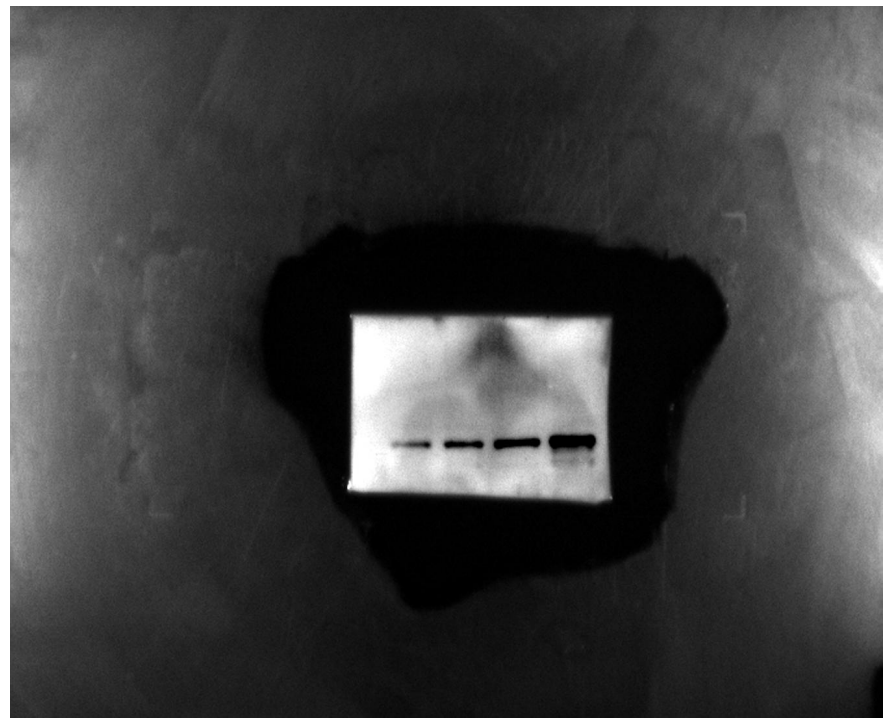

TTLL12

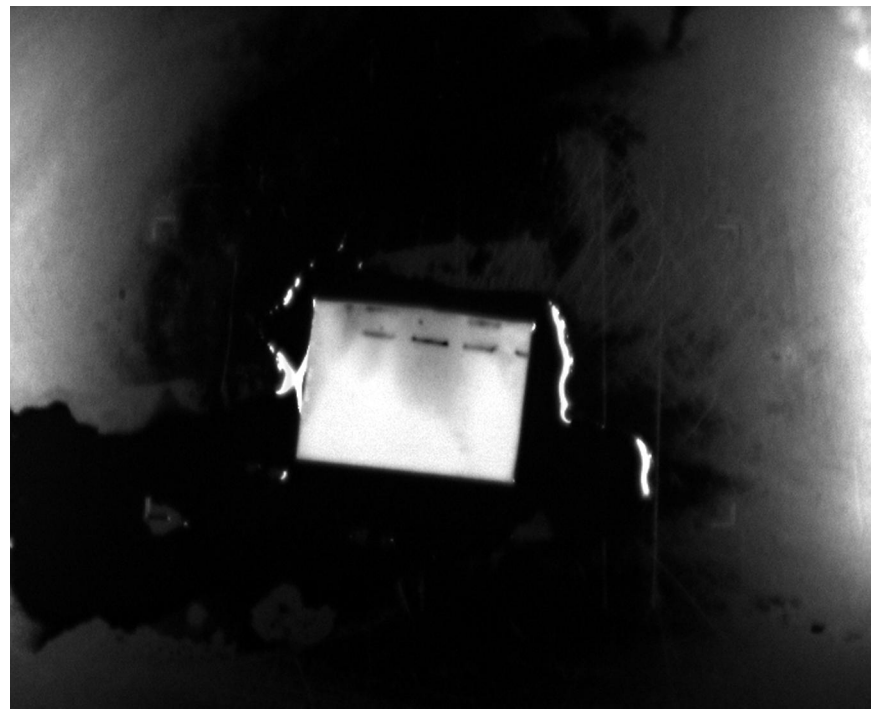

TTLL12

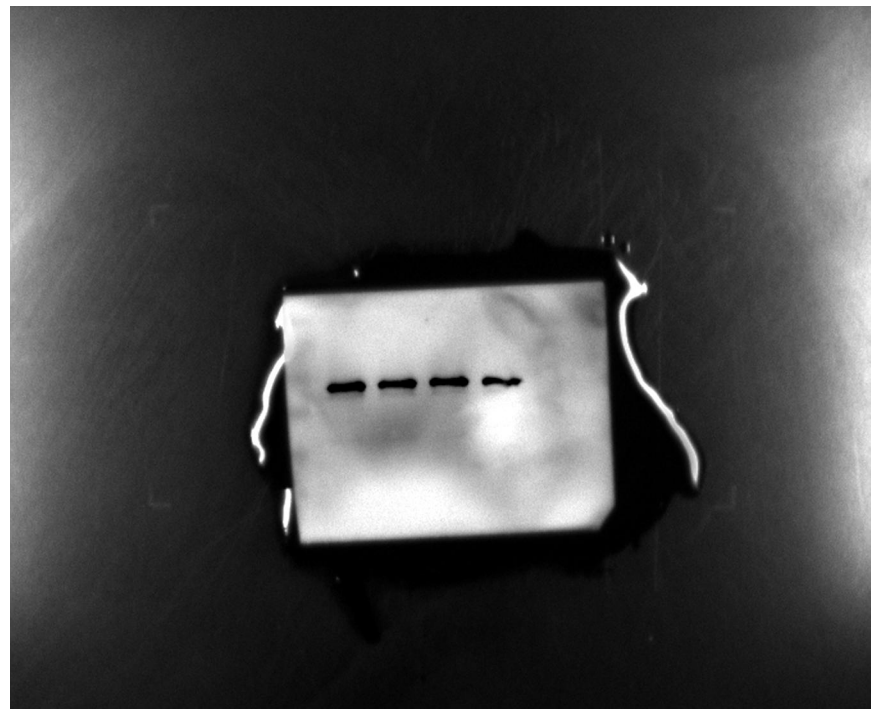

eEF1A1

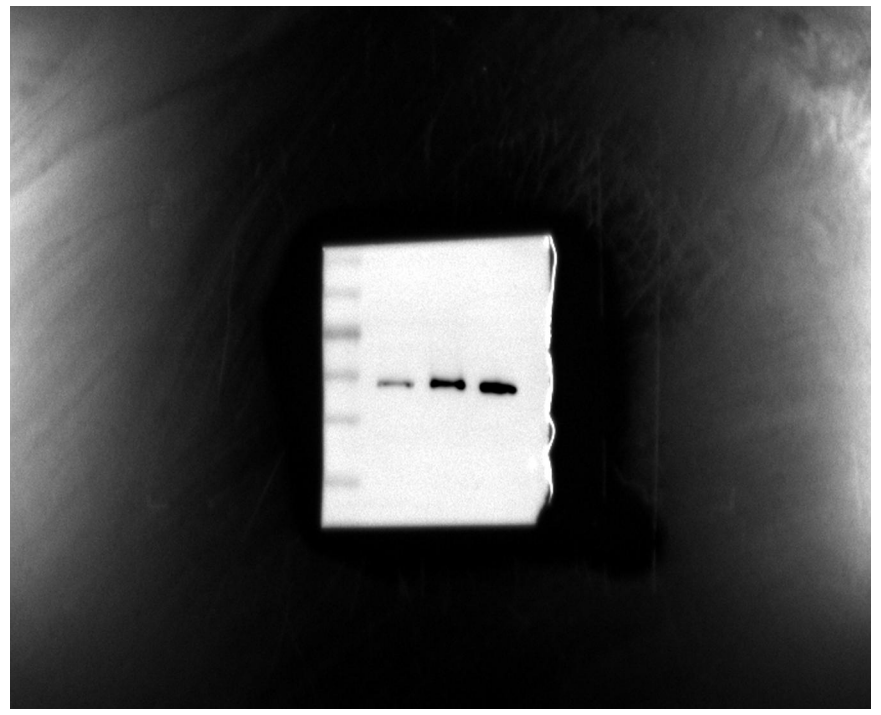

Figure 4J

J

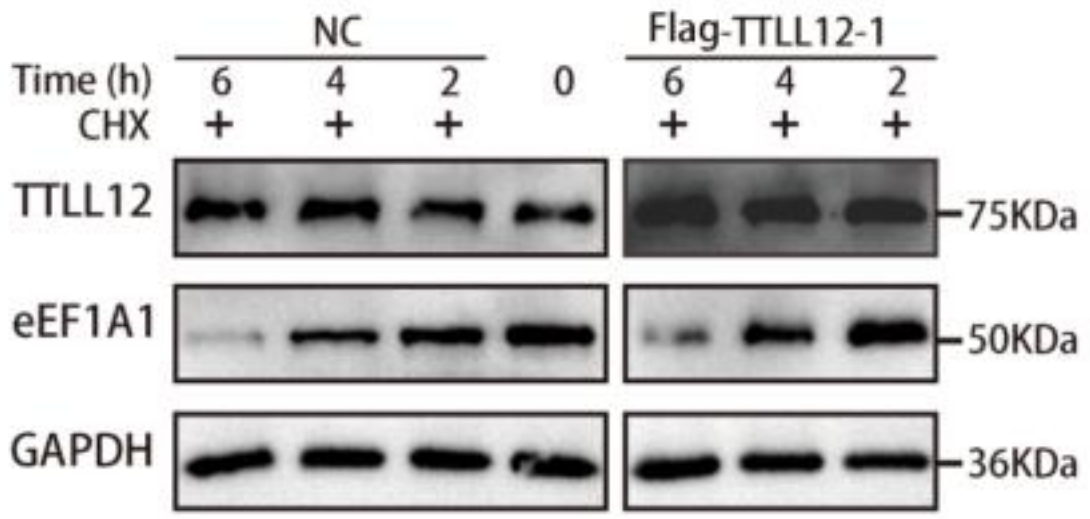

GAPDH

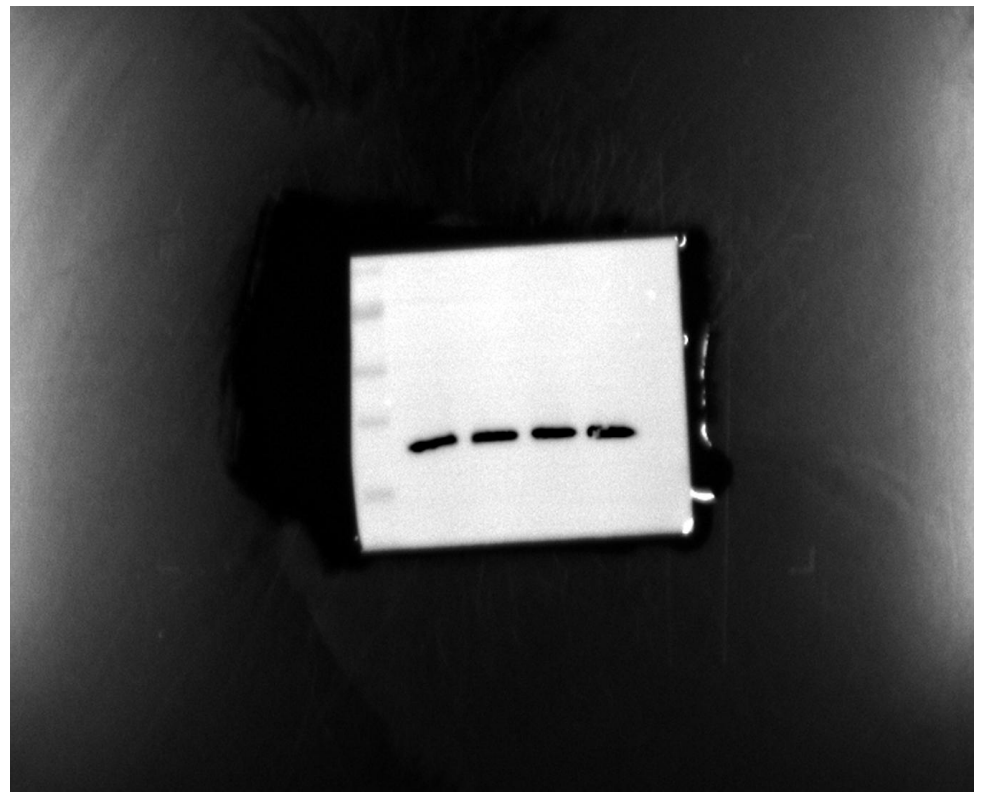

TTLL12

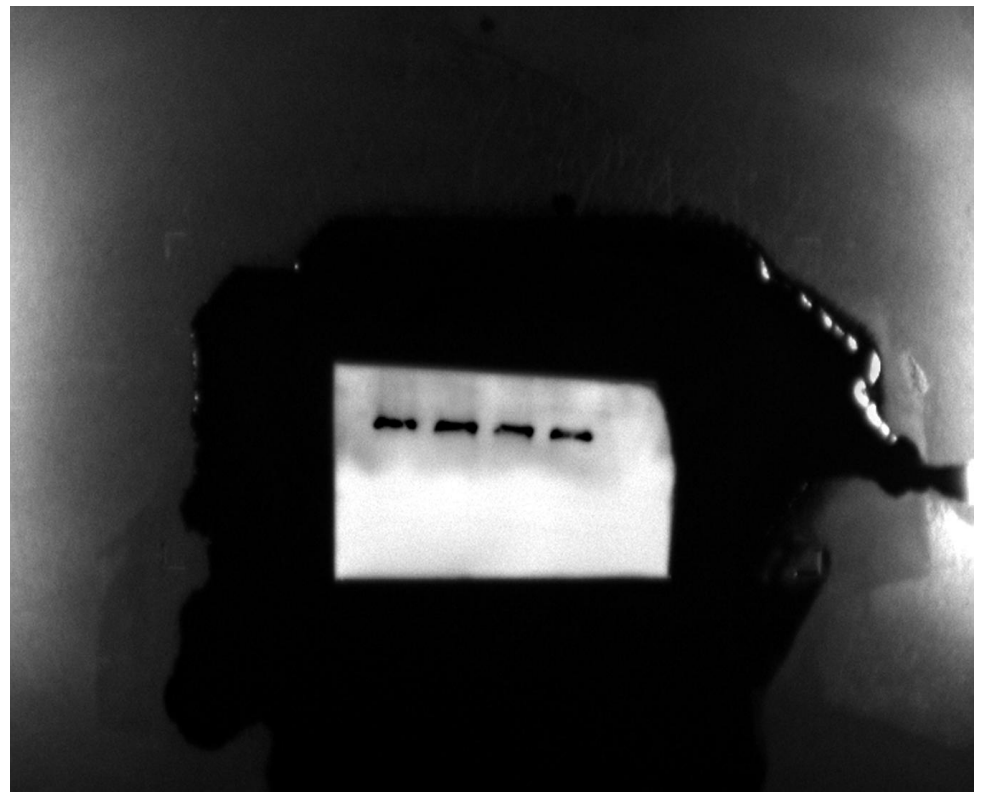

TTLL12

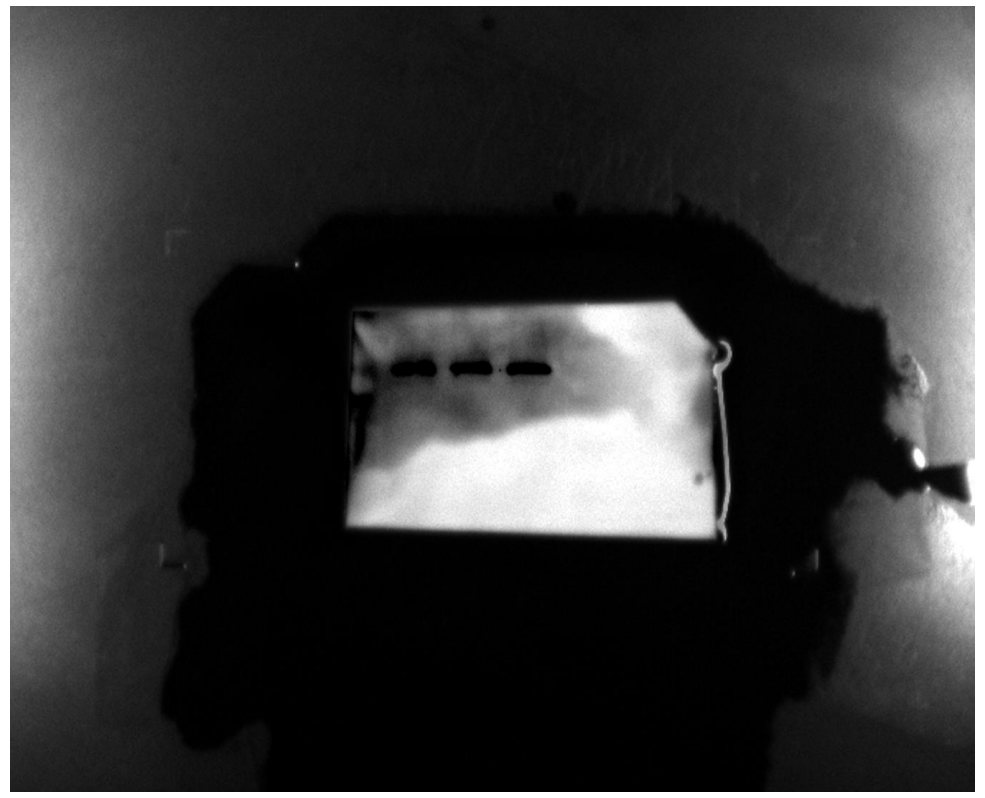

eEF1A1

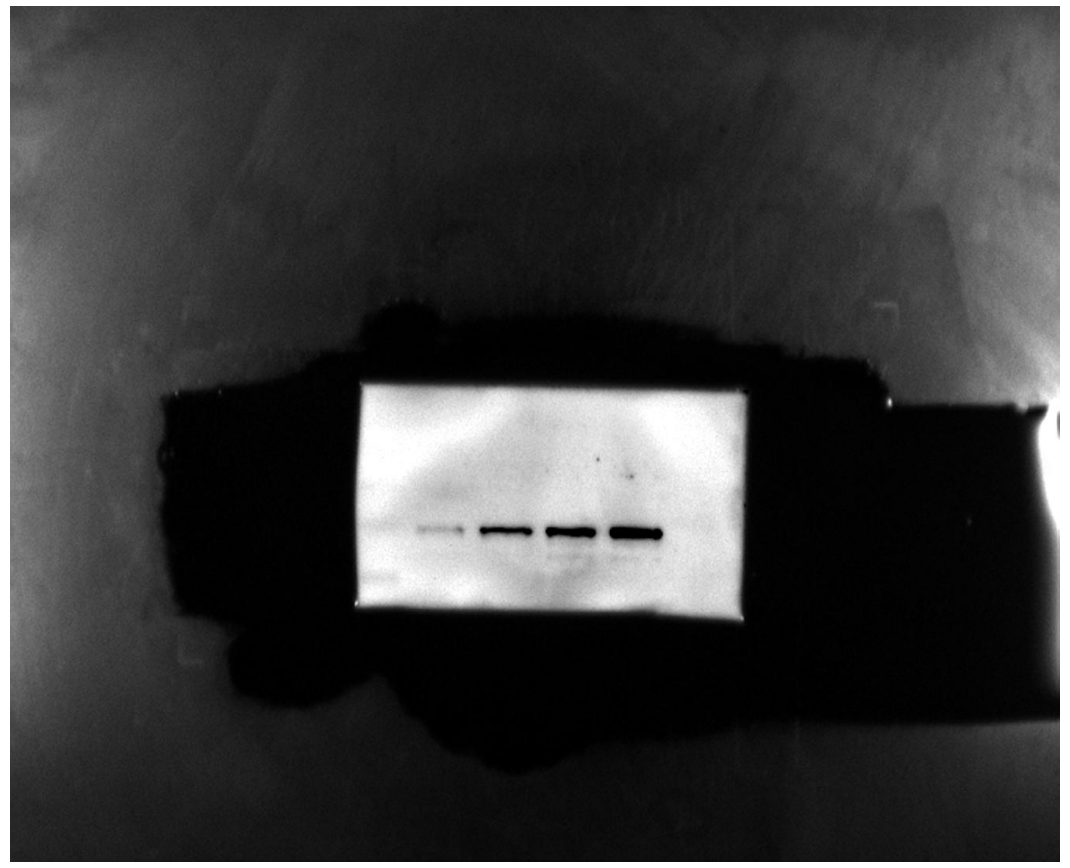

eEF1A1

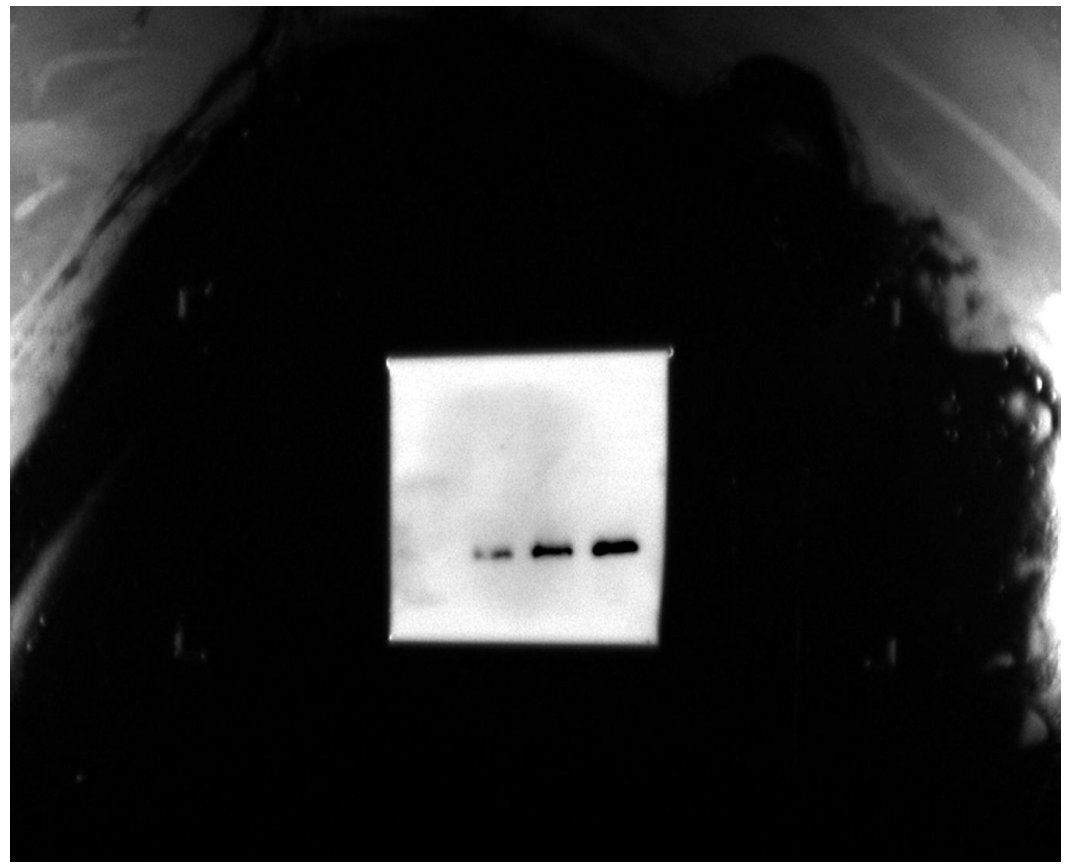

GAPDH

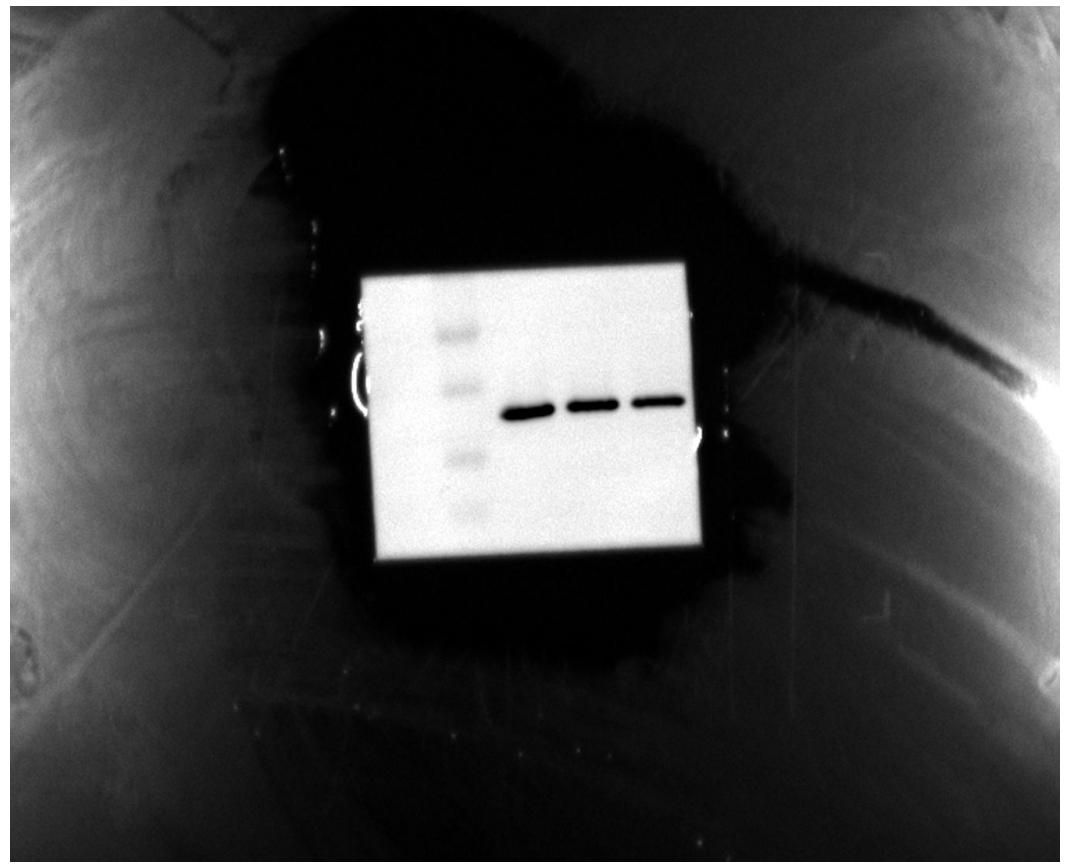

Figure 4K、4L

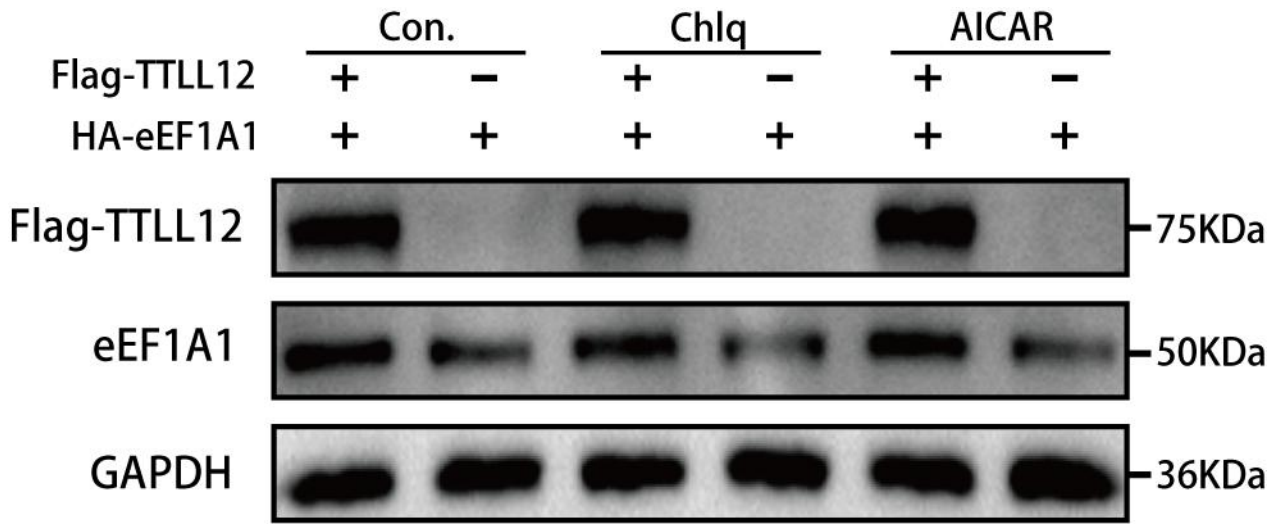

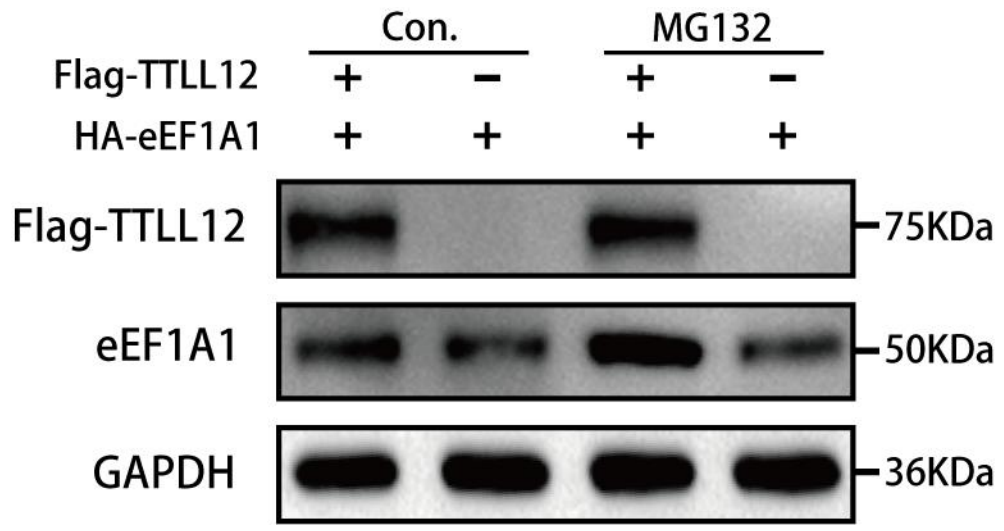

TTLL12

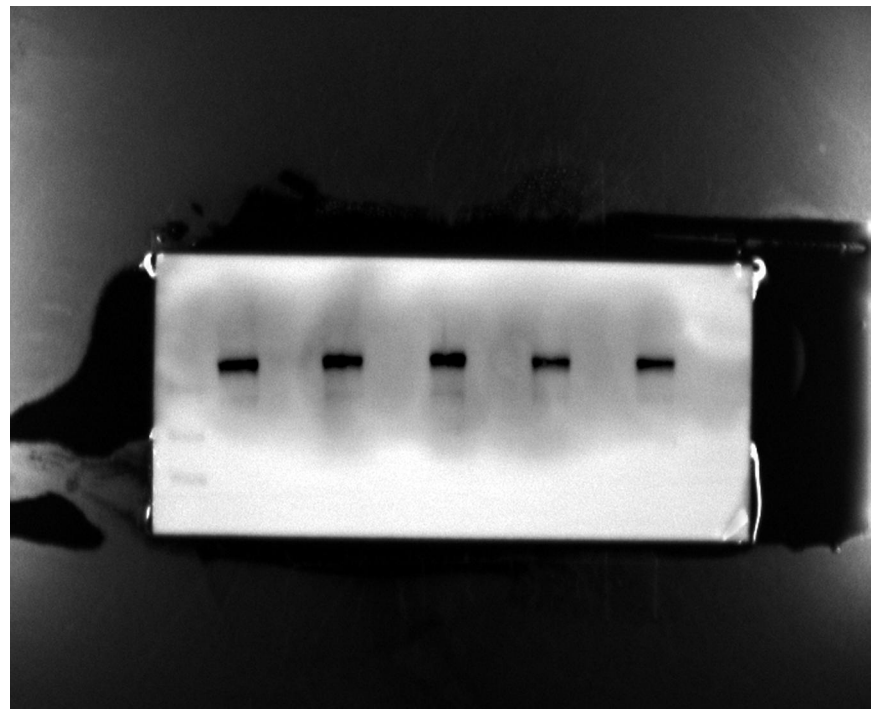

eEF1A1

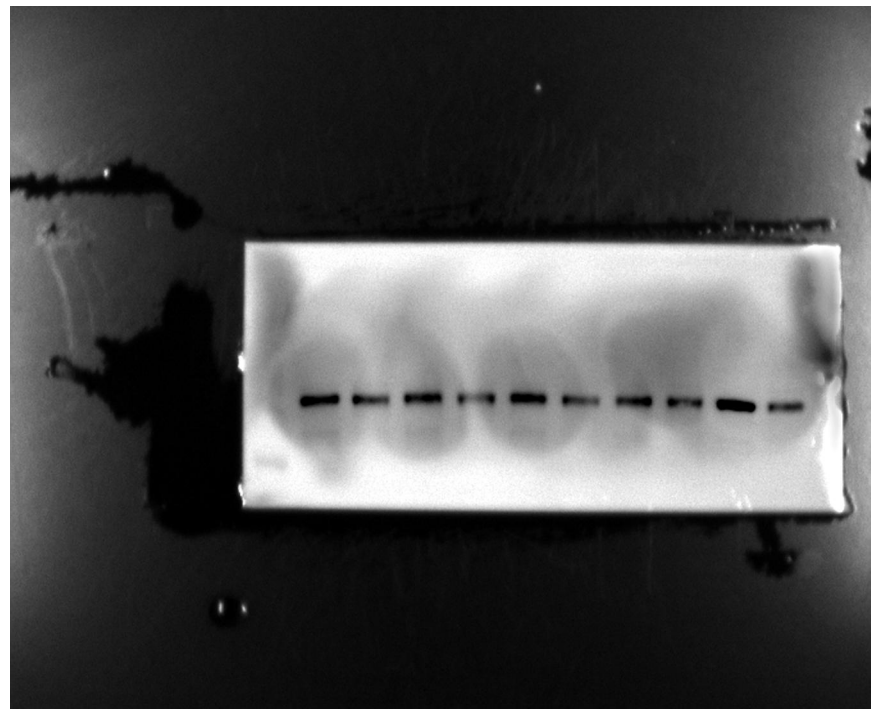

GAPDH

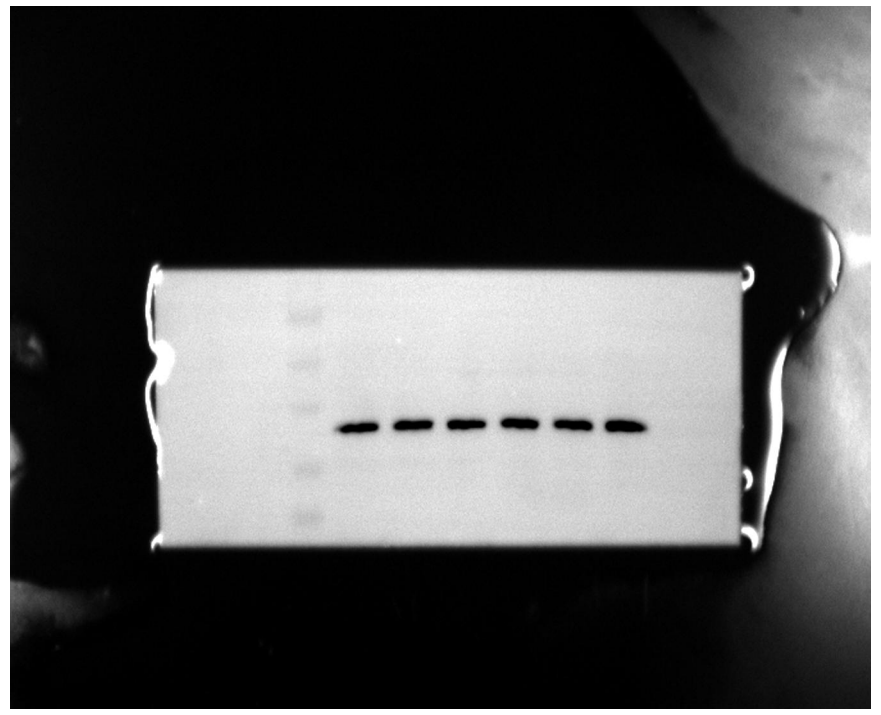

GAPDH

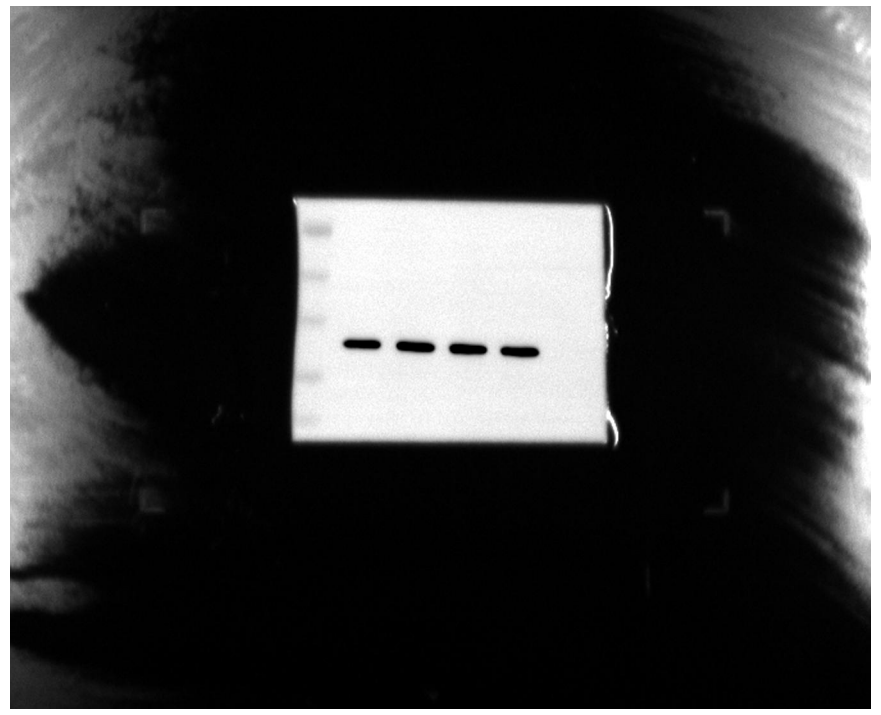

Figure 4M

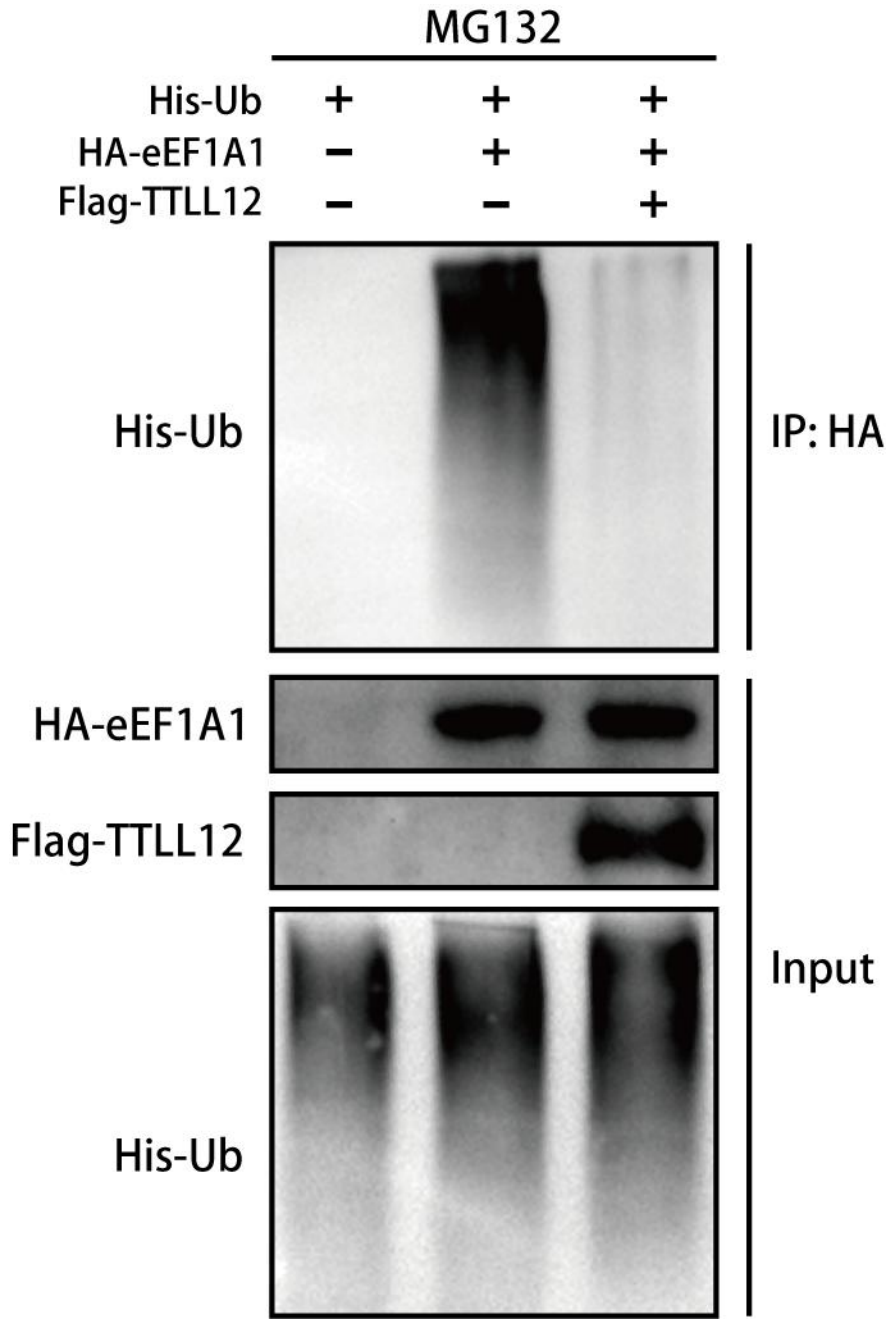

Flag-TTLL12

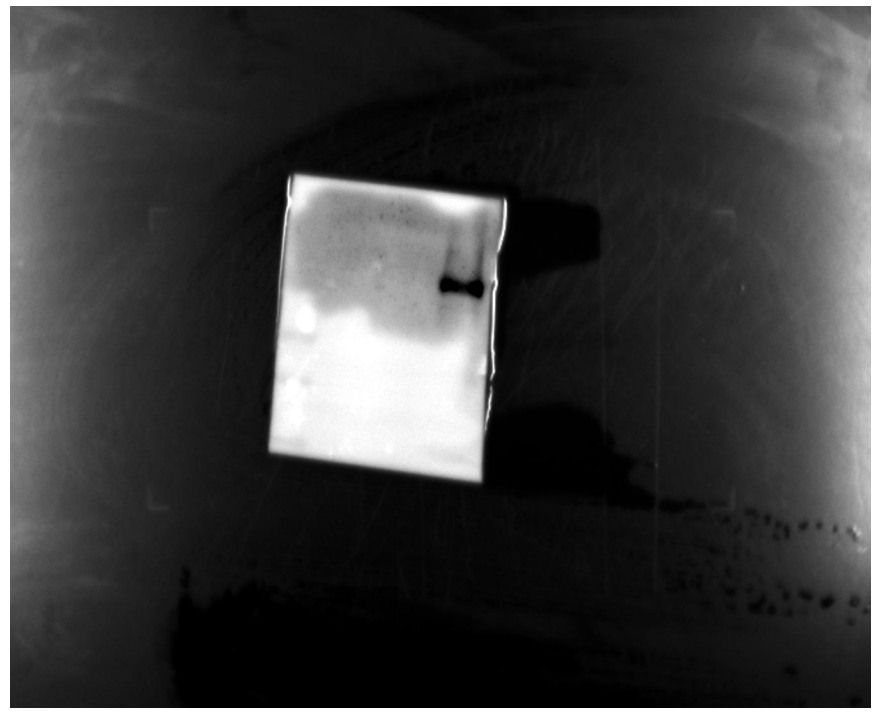

His-Ub

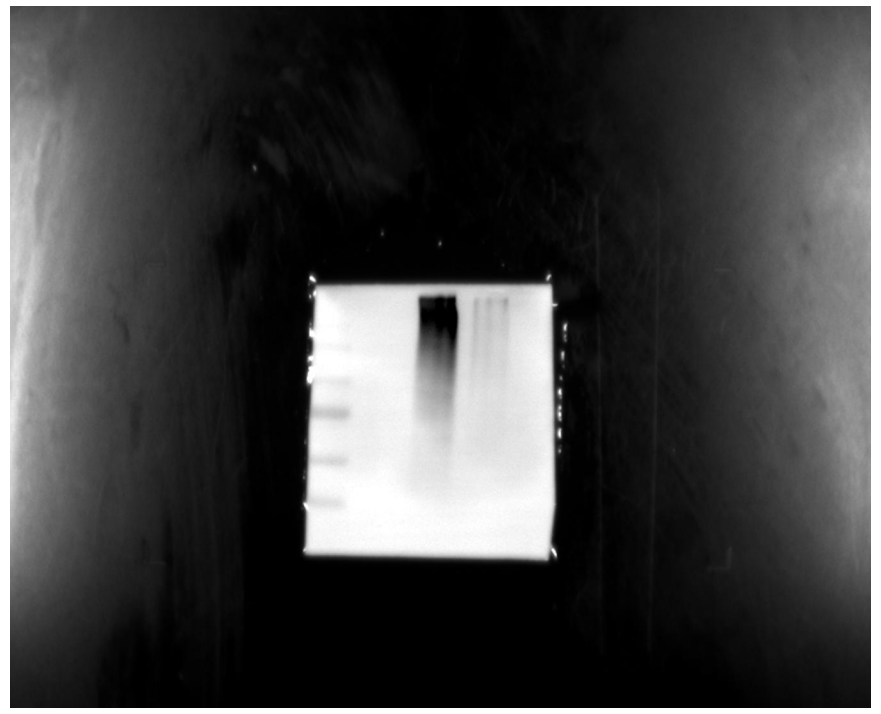

HA-eEF1A1

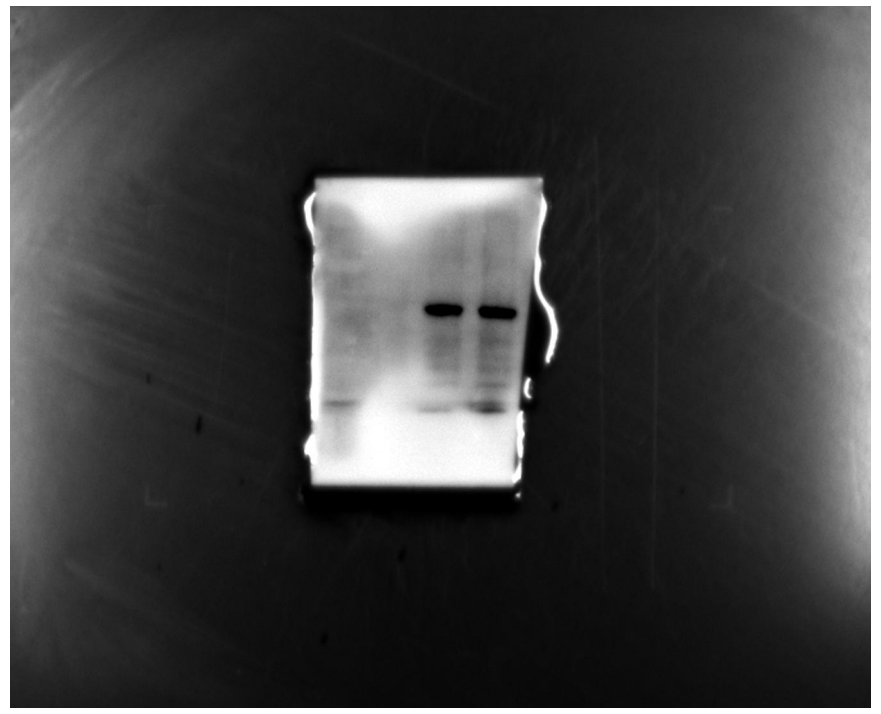

His-Ub

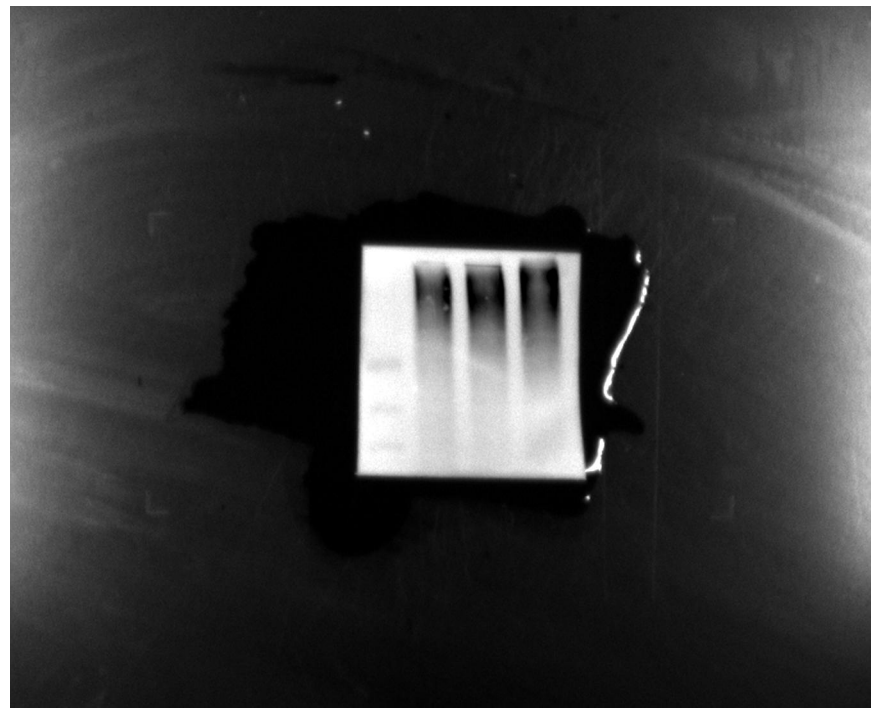

Figure 5A

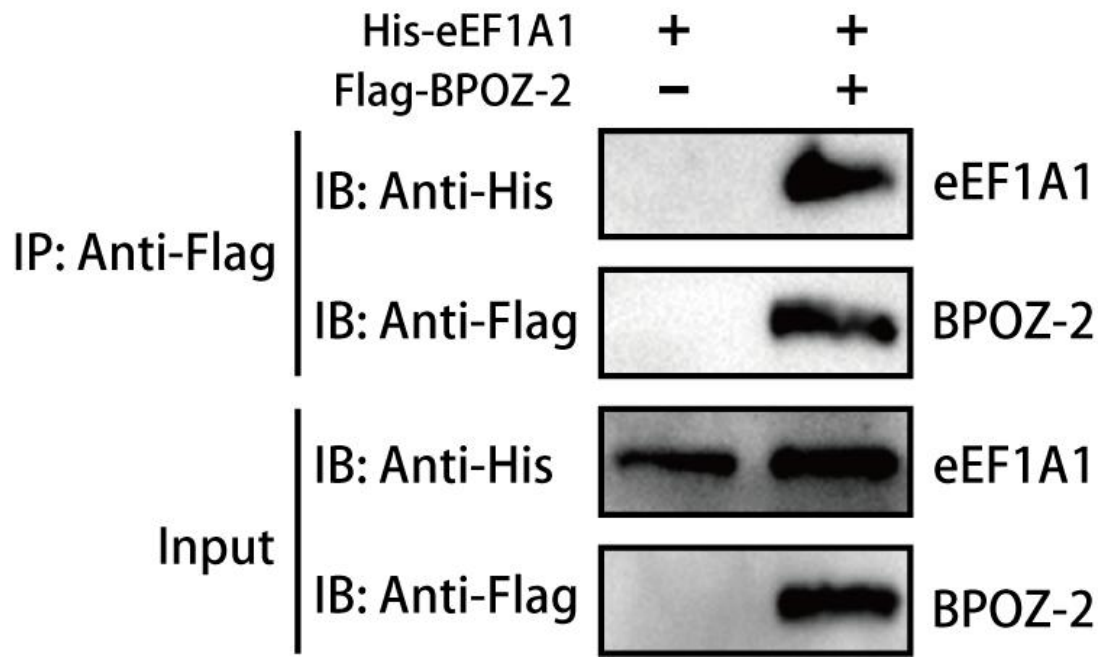

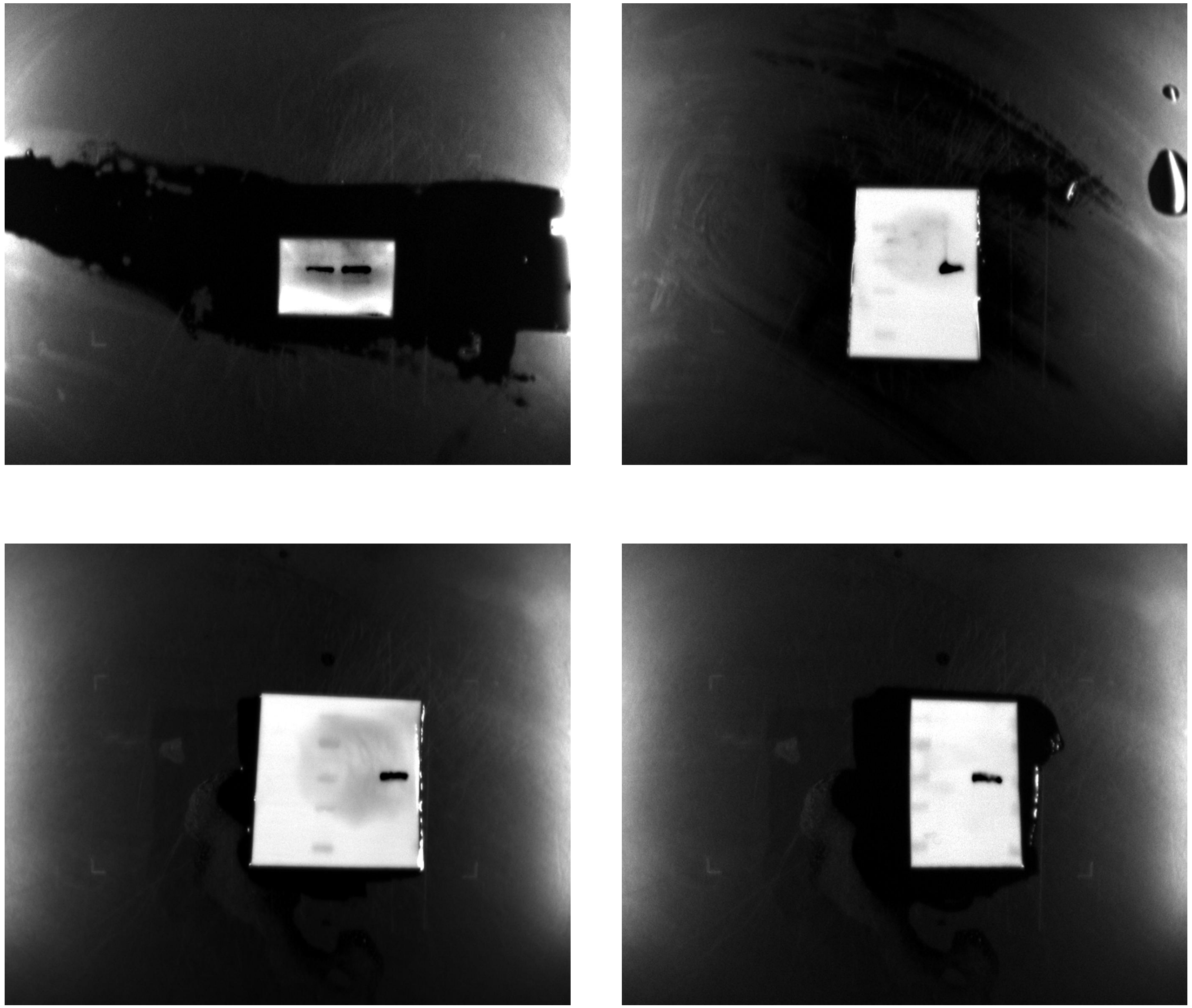

His-eEF1A1

Flag-BPOZ-2

Figure 5B

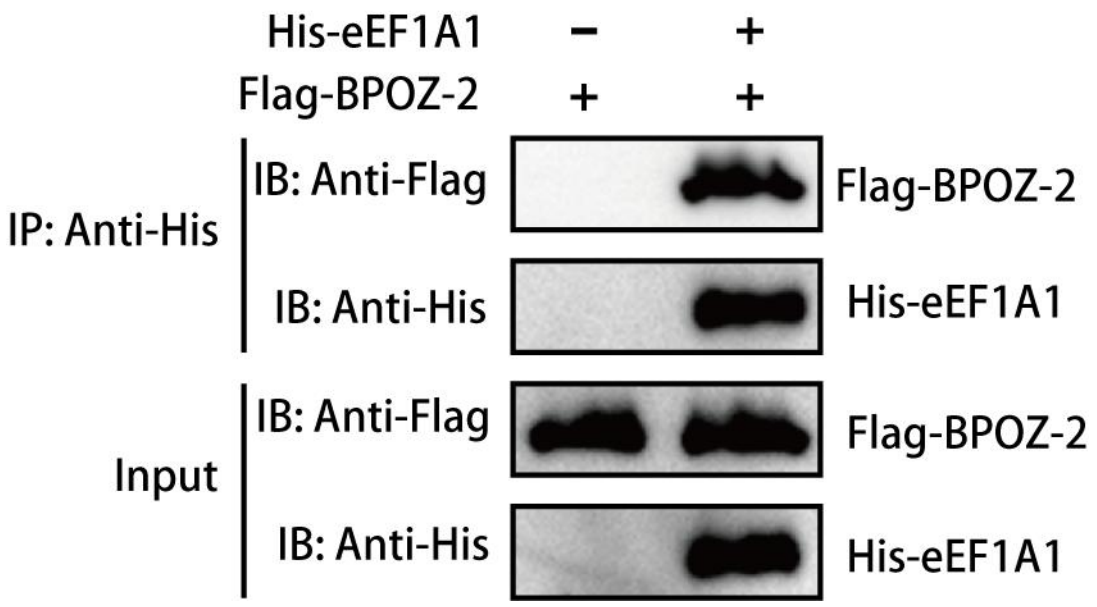

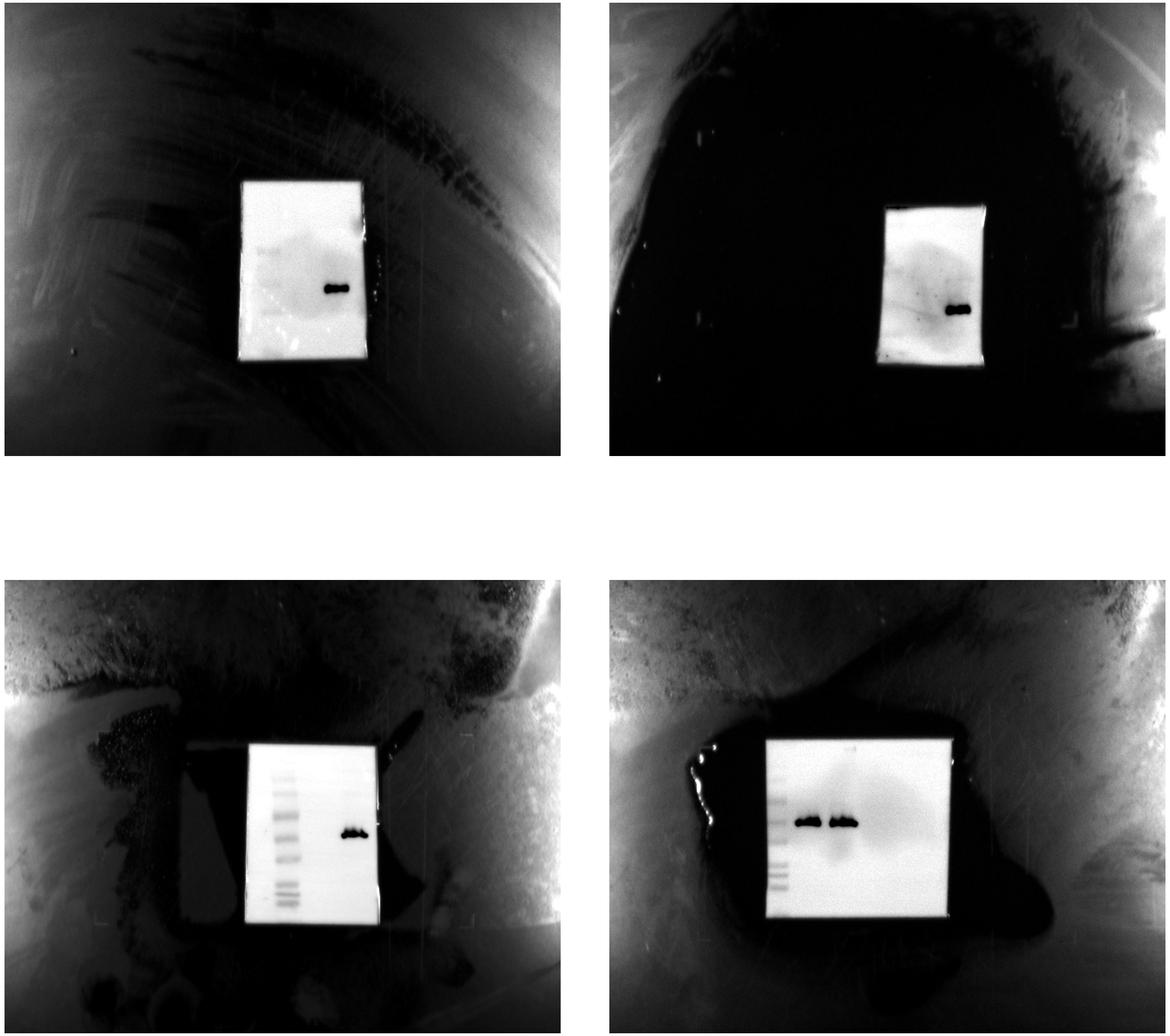

His-eEF1A1

Flag-BPOZ-2

Figure 5D

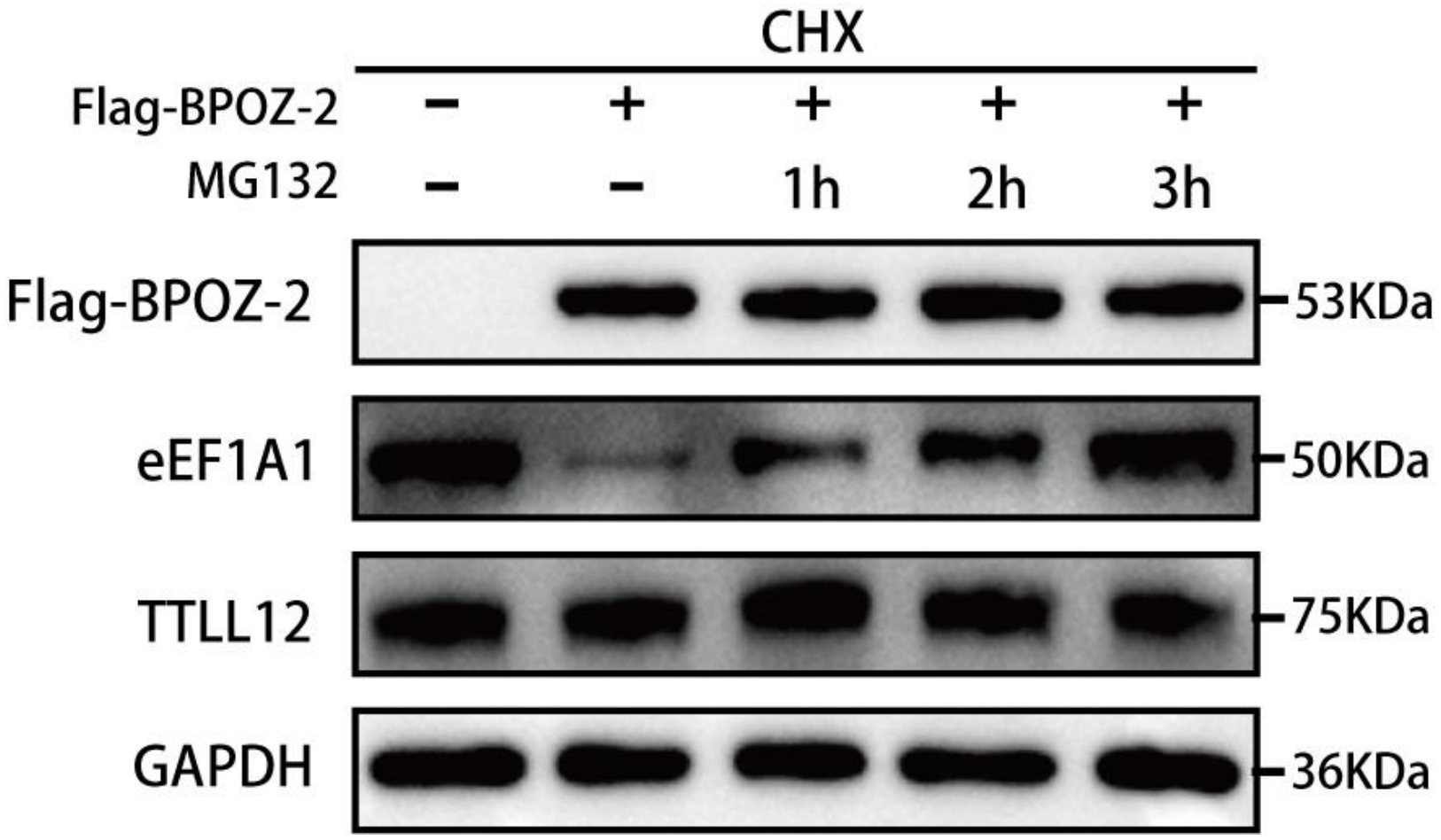

Flag-BPOZ-2

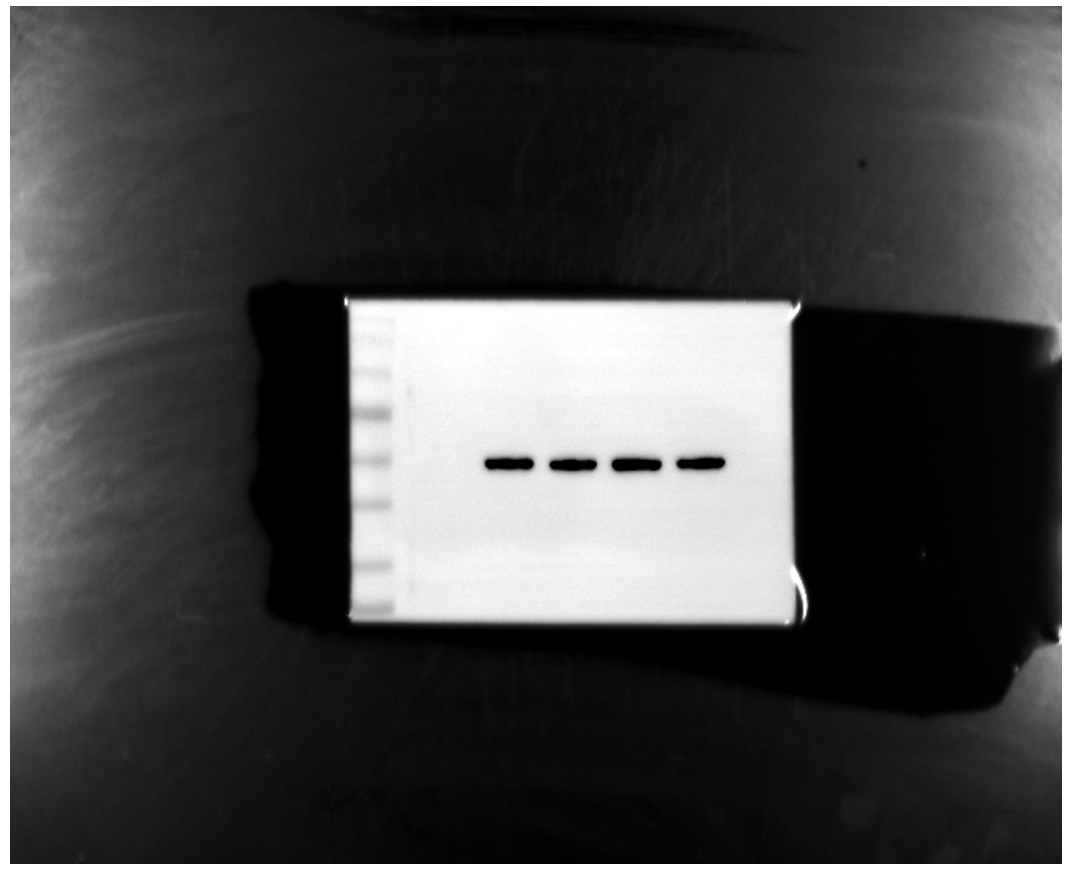

eEF1A1

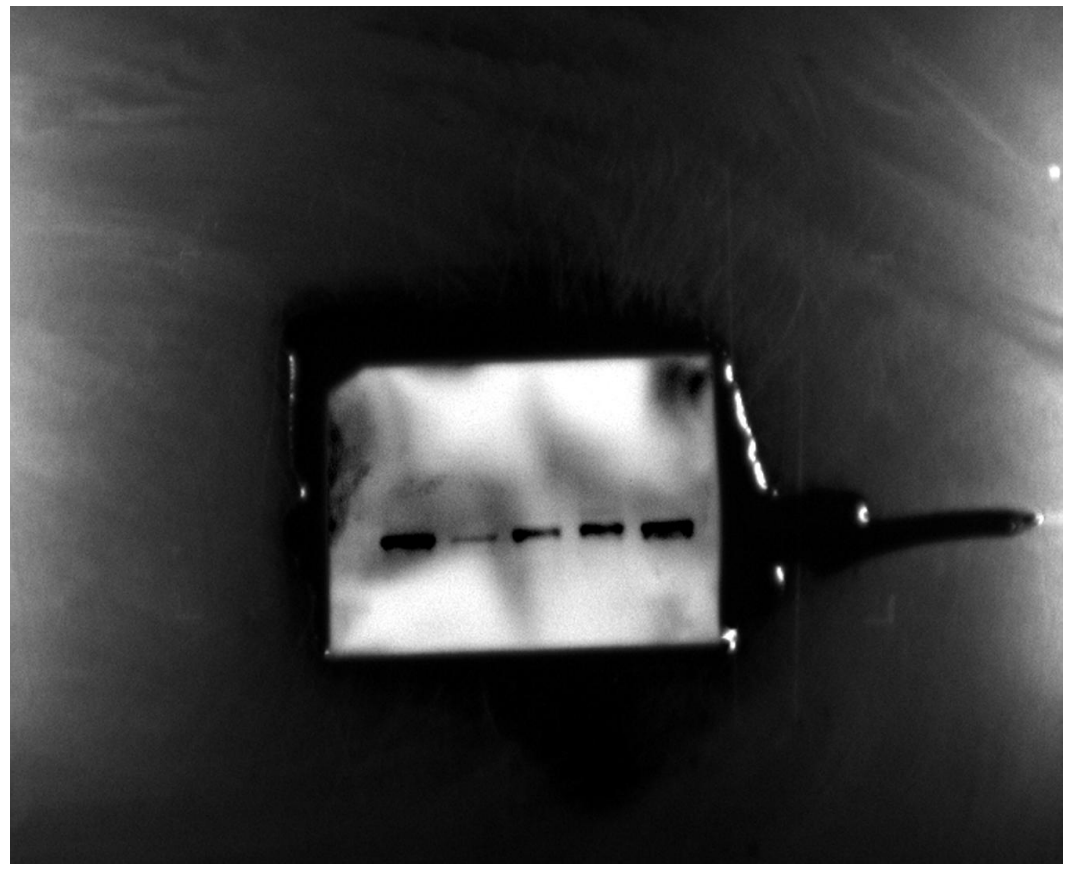

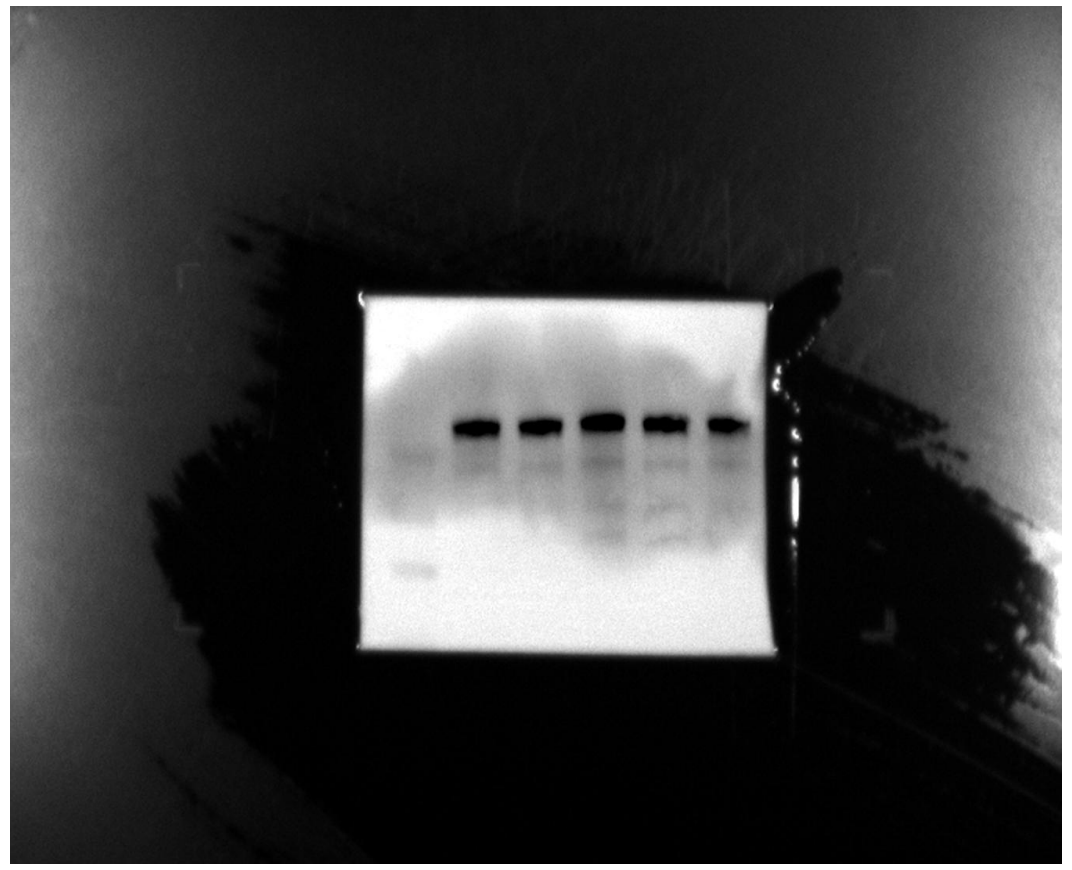

TTLL12

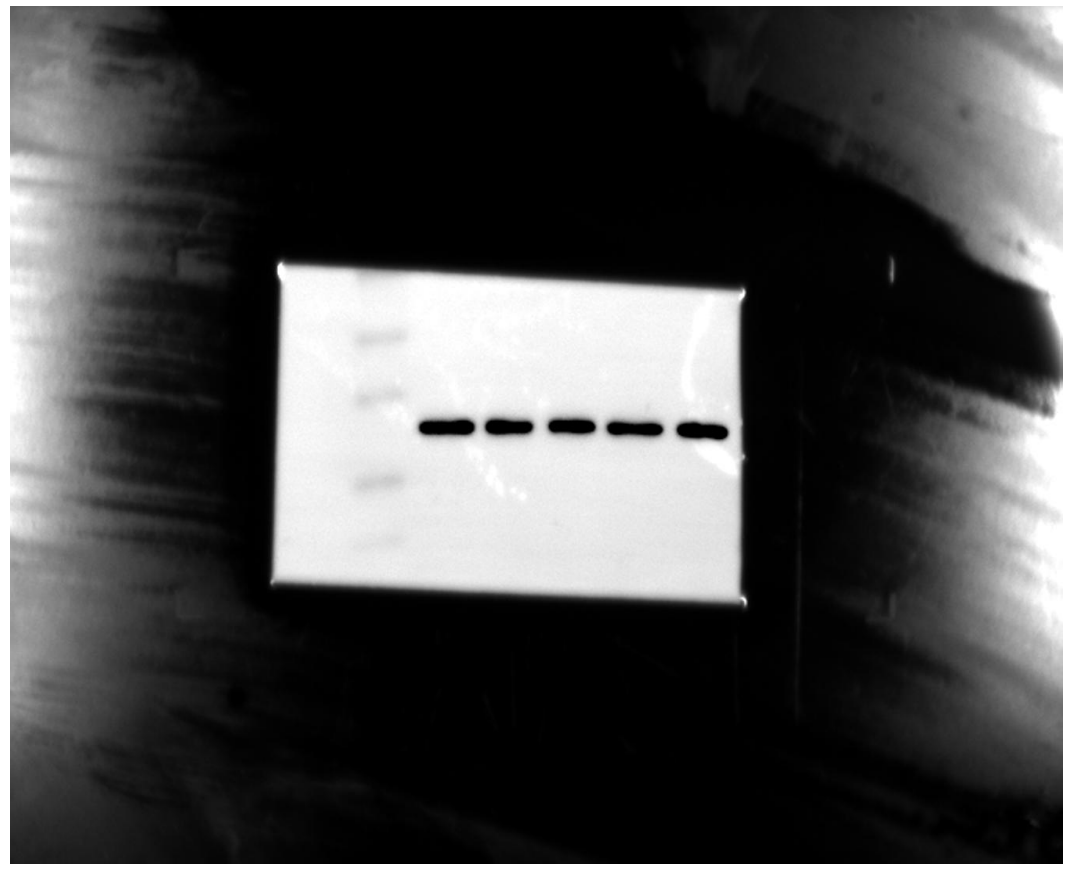

GAPDH

Figure 5E

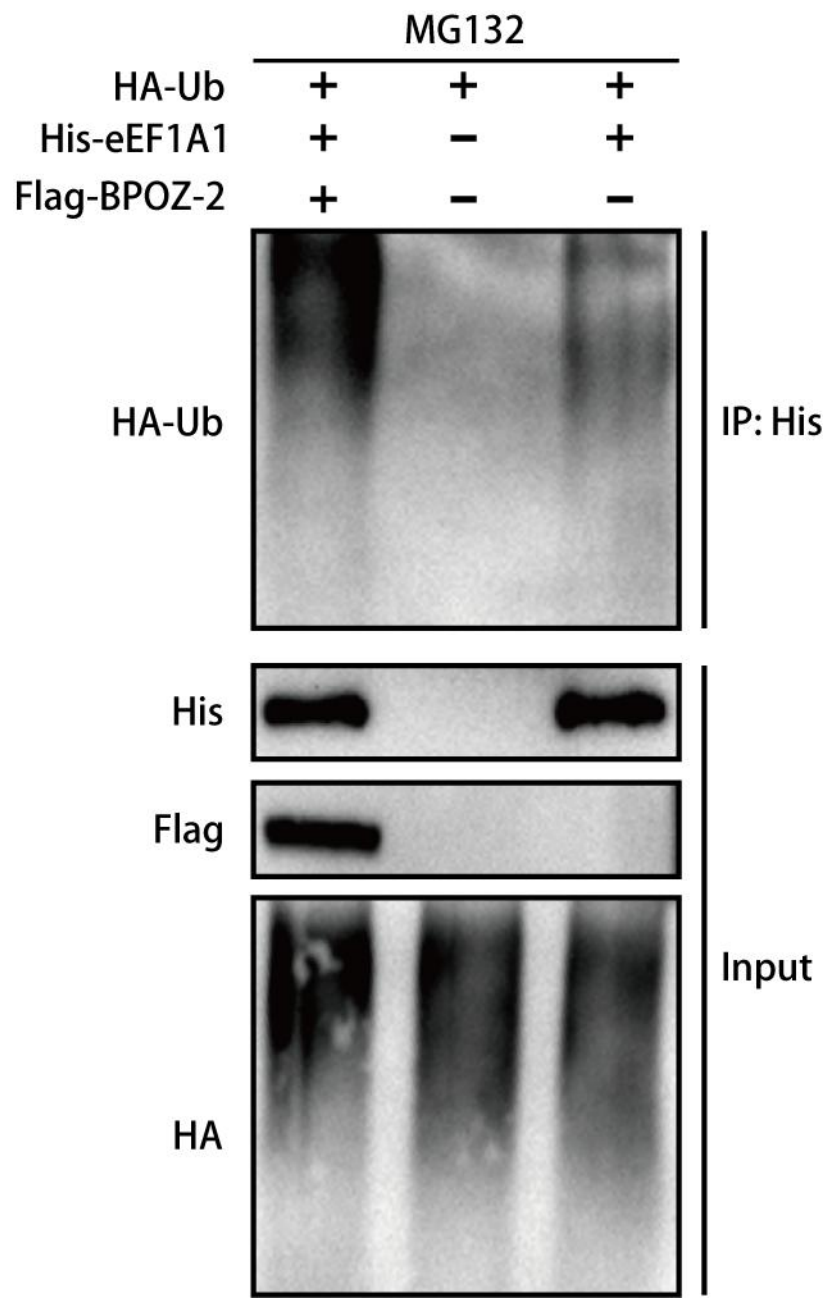

MG132
HA-Ub + + +
His-eEF1A1 + − +
Flag-BPOZ-2 + − −
HA-Ub
IP: His
His
Flag
HA
Input

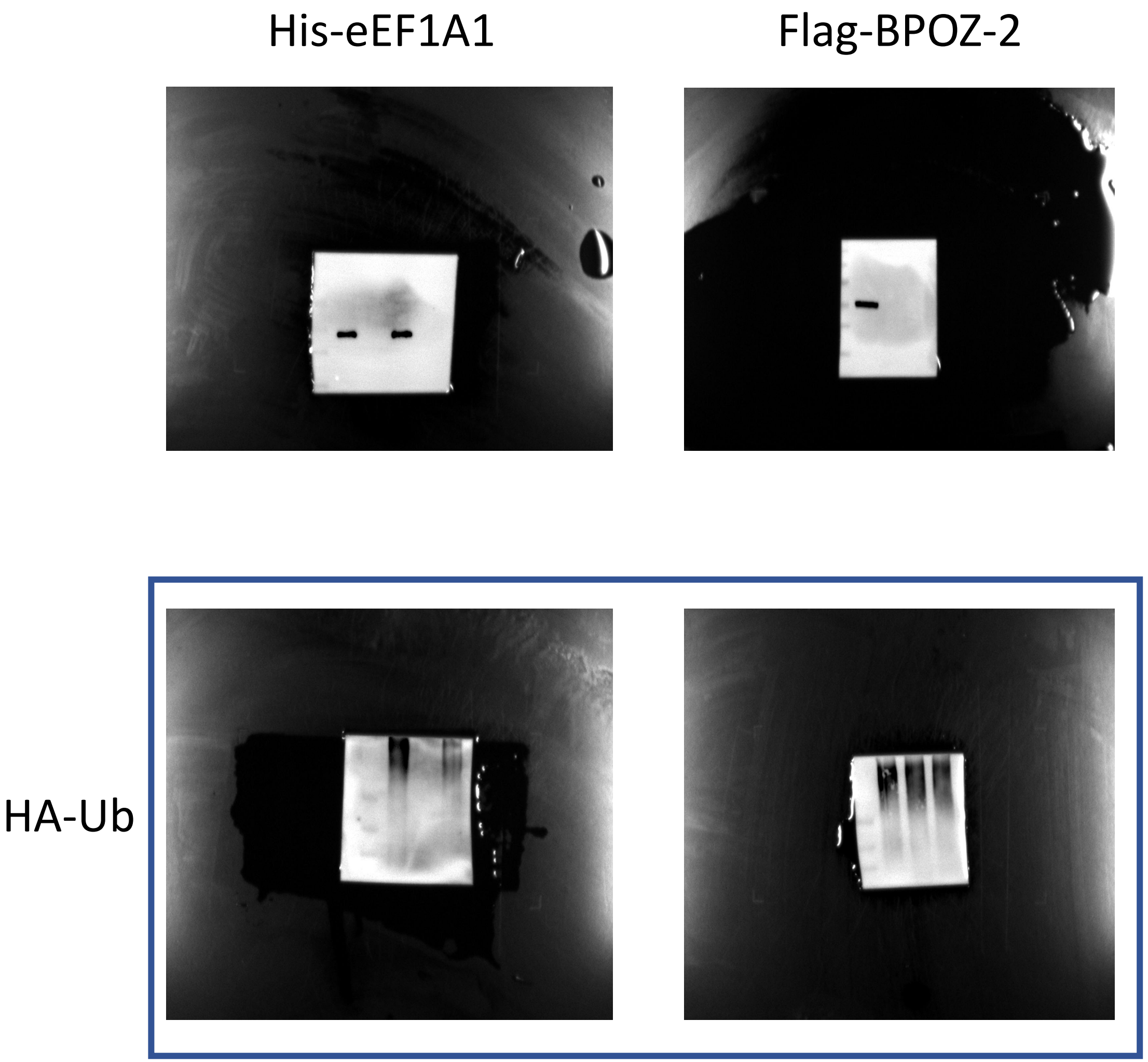

His-eEF1A1
Flag-BPOZ-2
HA-Ub

Figure 5G

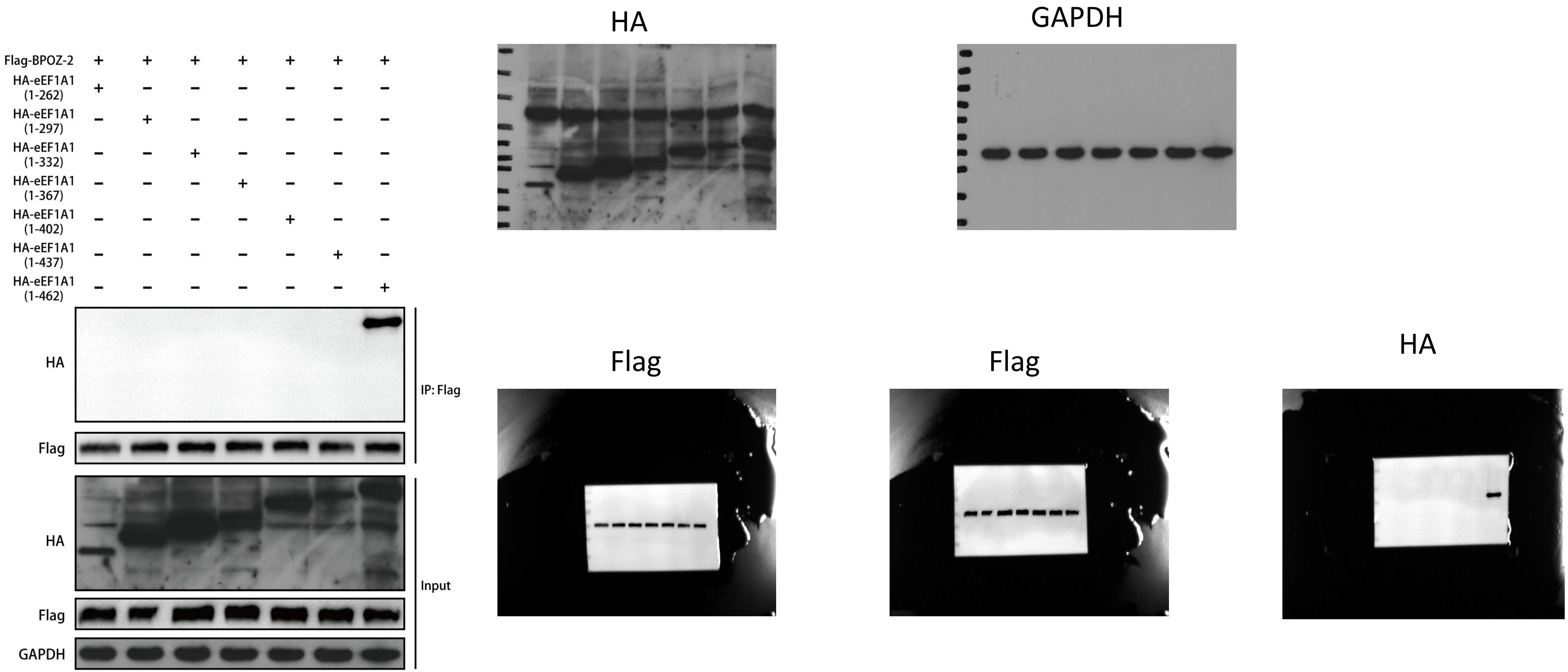
Flag-BPOZ-2
HA-eEF1A1 (1-262)
HA-eEF1A1 (1-297)
HA-eEF1A1 (1-332)
HA-eEF1A1 (1-367)
HA-eEF1A1 (1-402)
HA-eEF1A1 (1-437)
HA-eEF1A1 (1-462)
HA
Flag
IP: Flag
HA
Flag
GAPDH
Input
HA
GAPDH
Flag
Flag
HA

Figure 5H

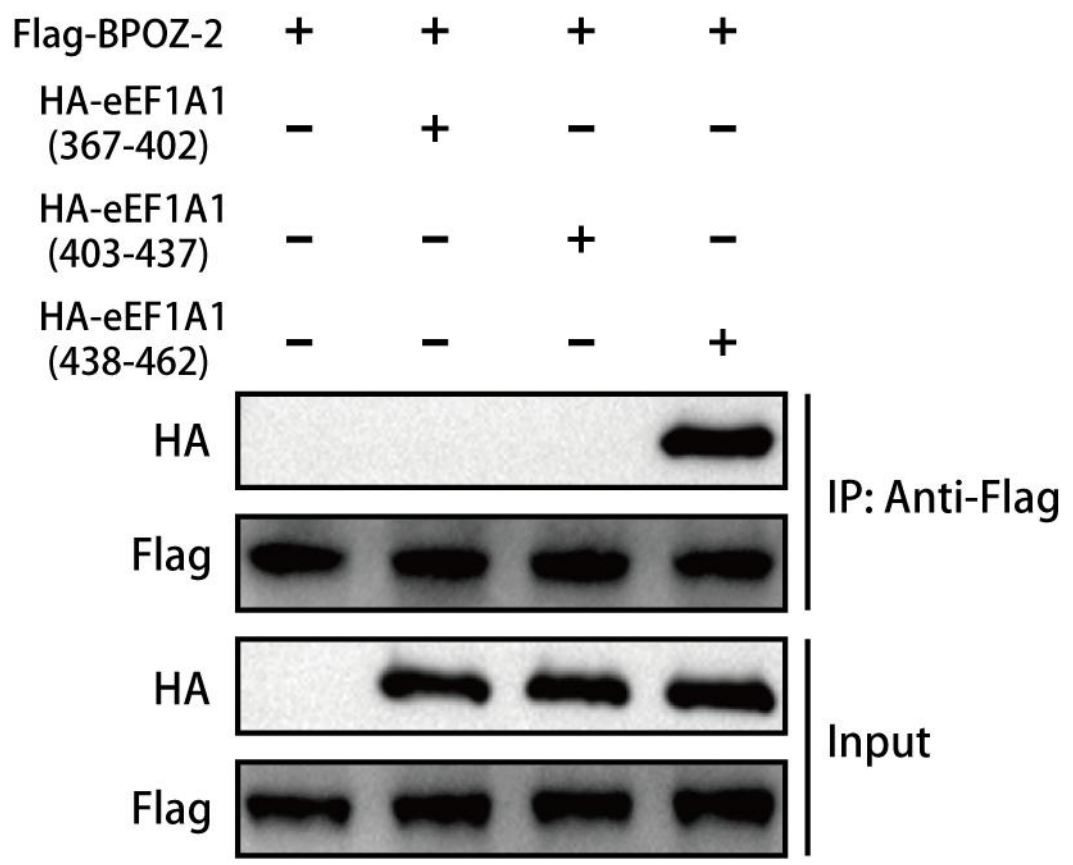

Flag

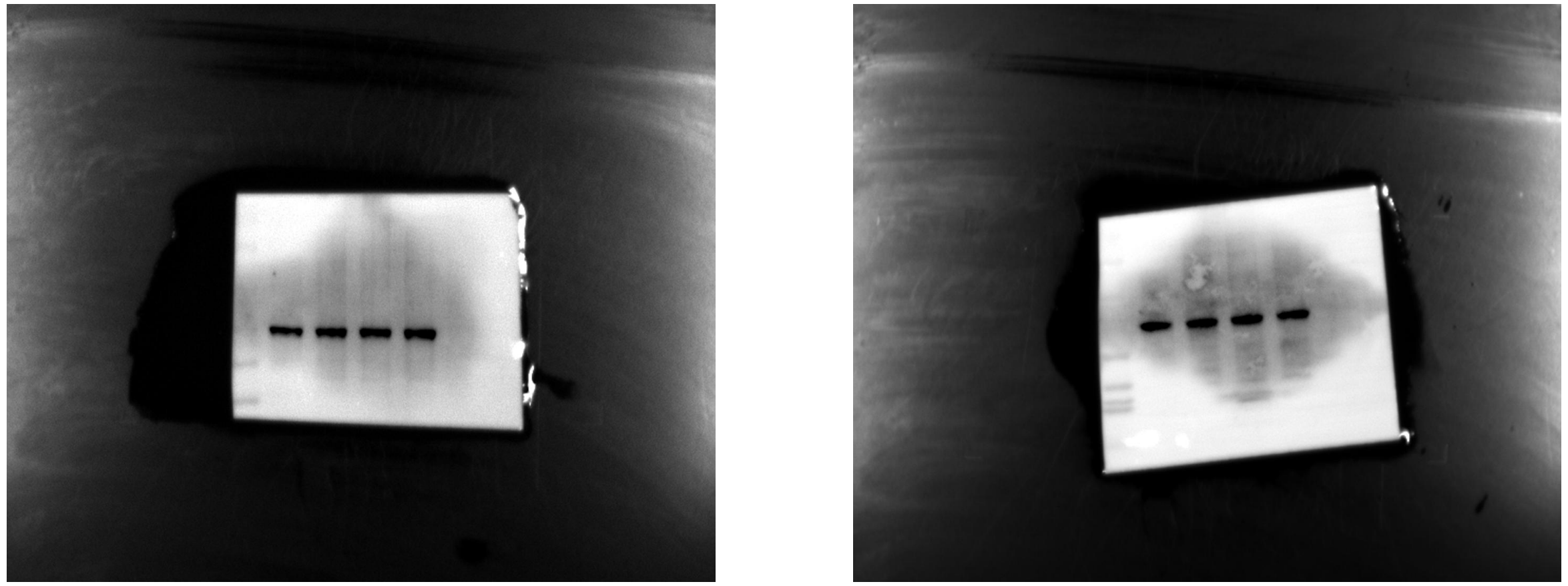

HA

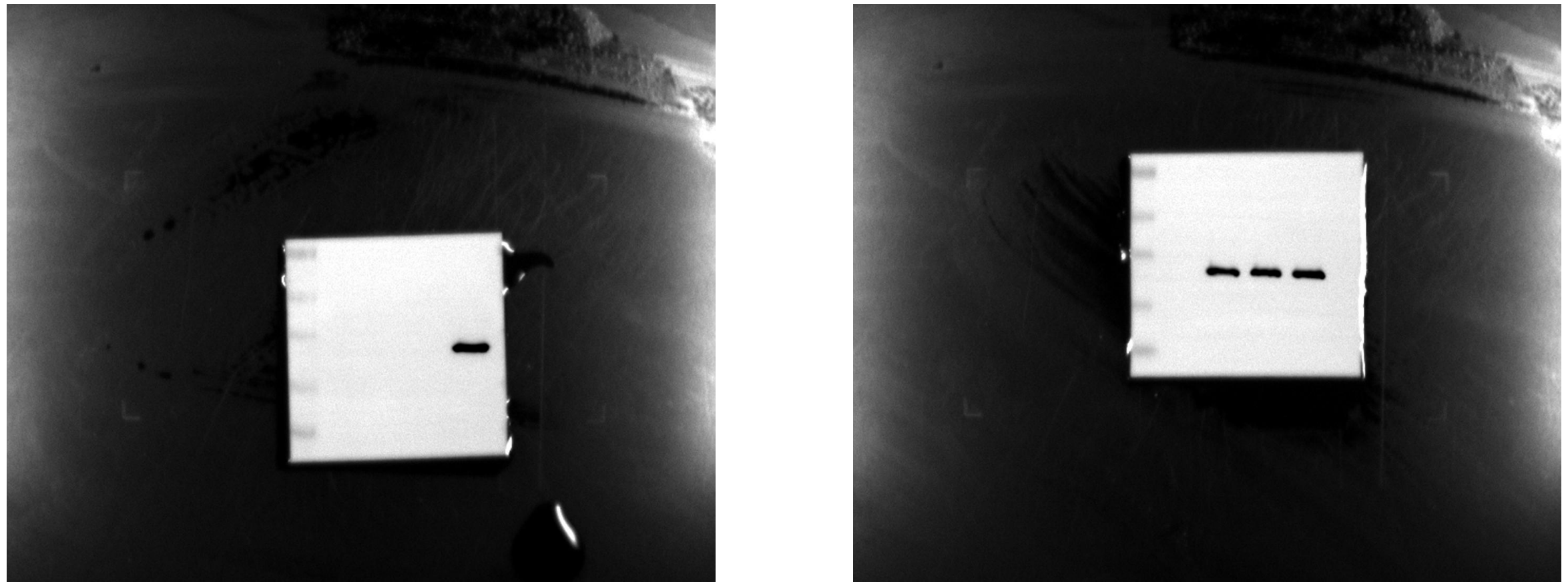

Figure 5I

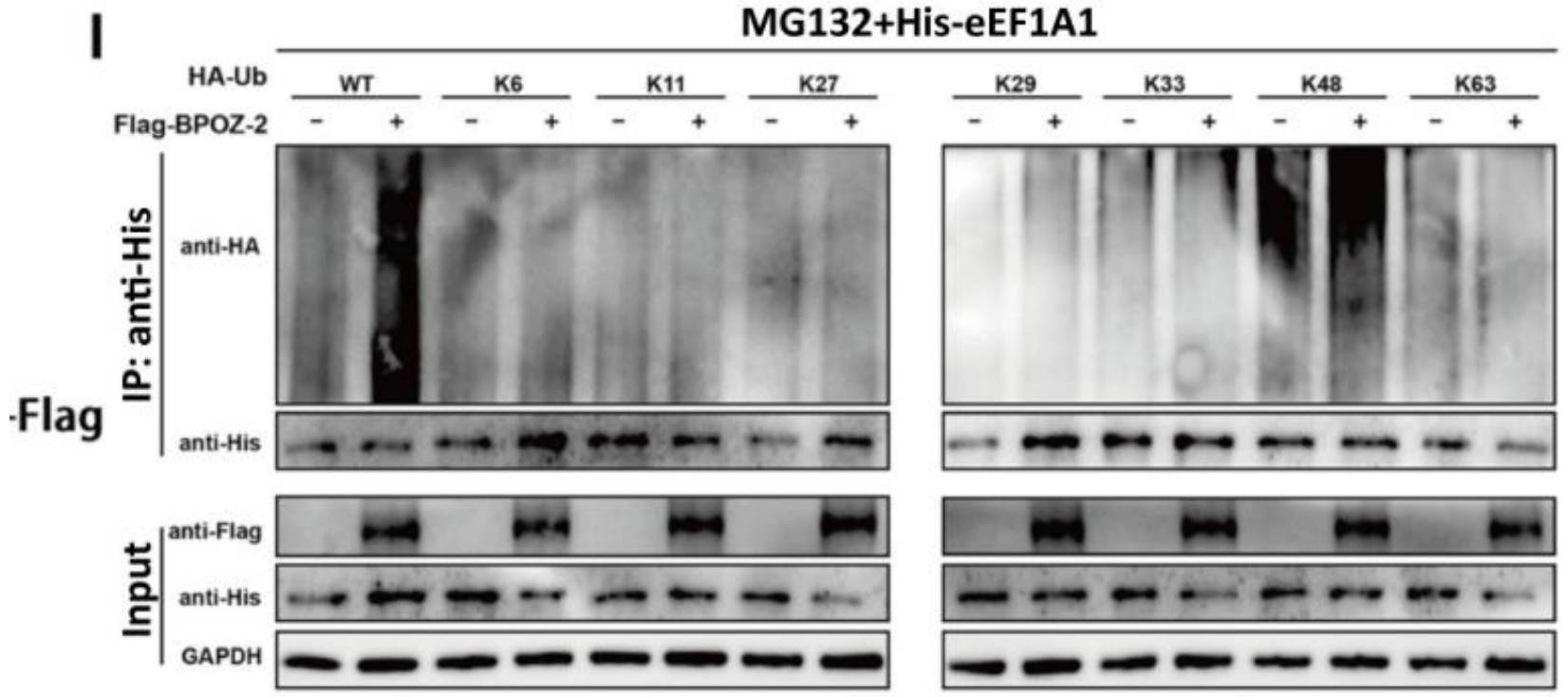

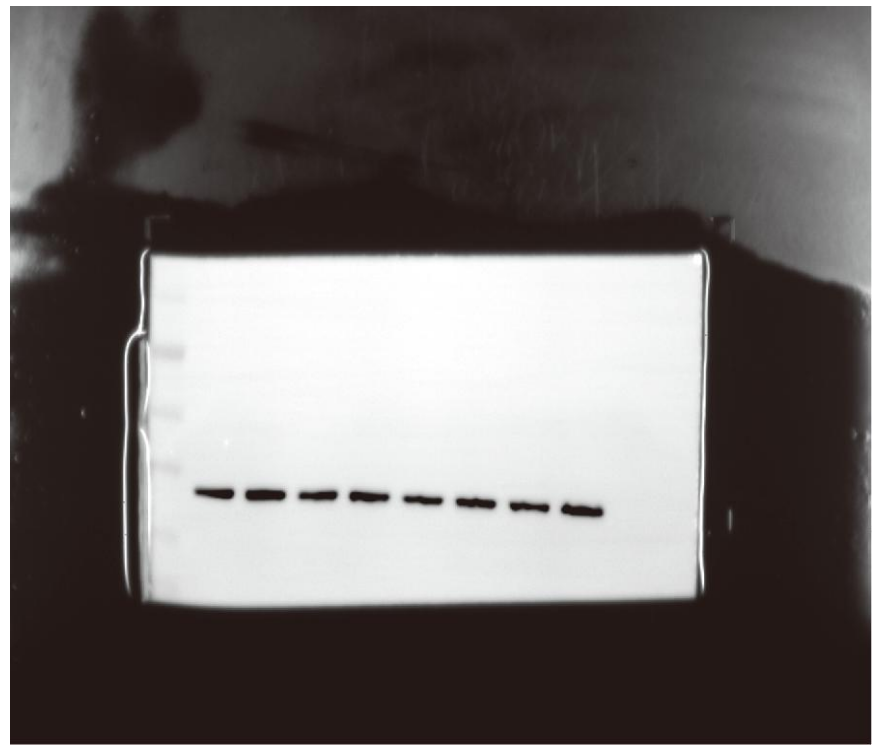

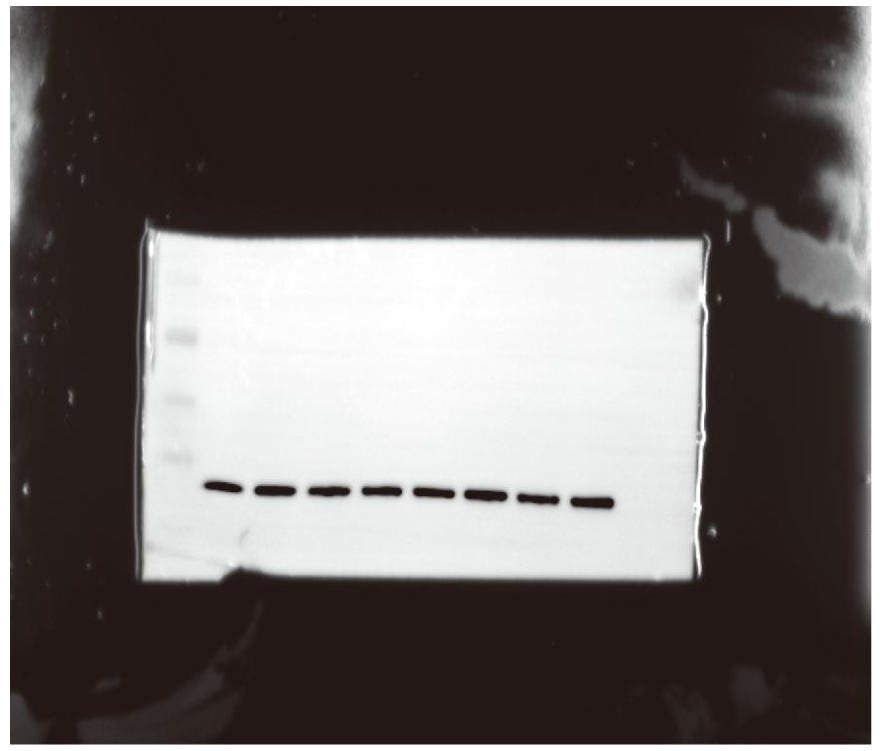

GAPDH

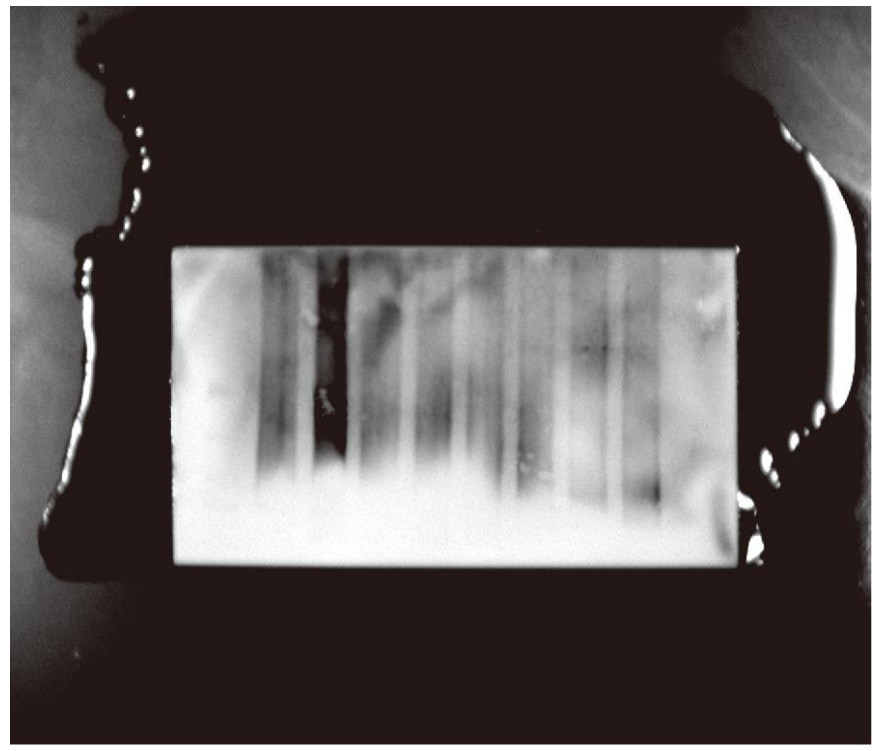

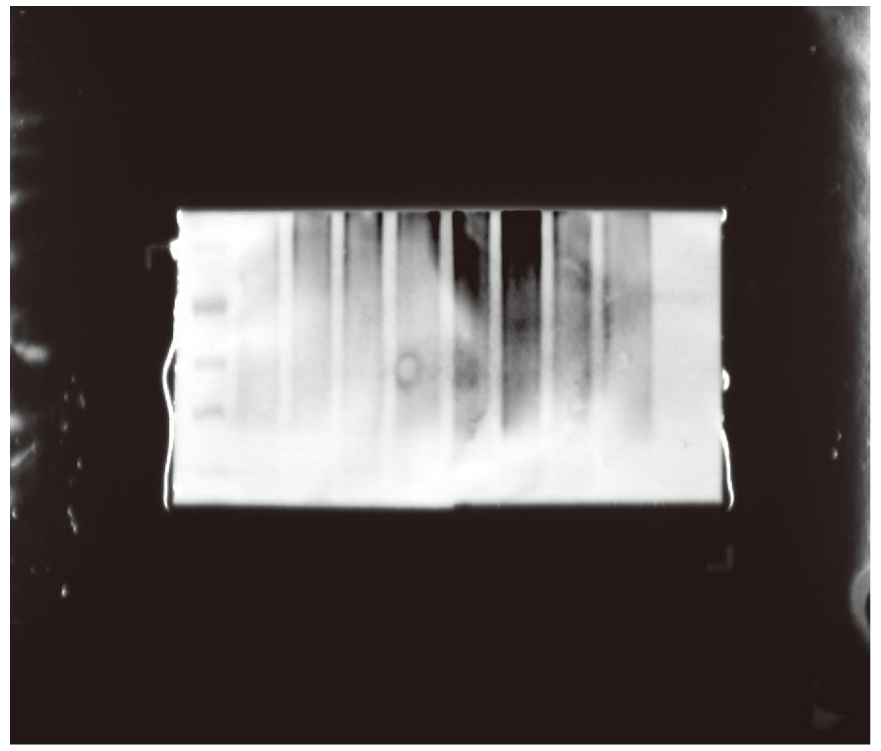

HA

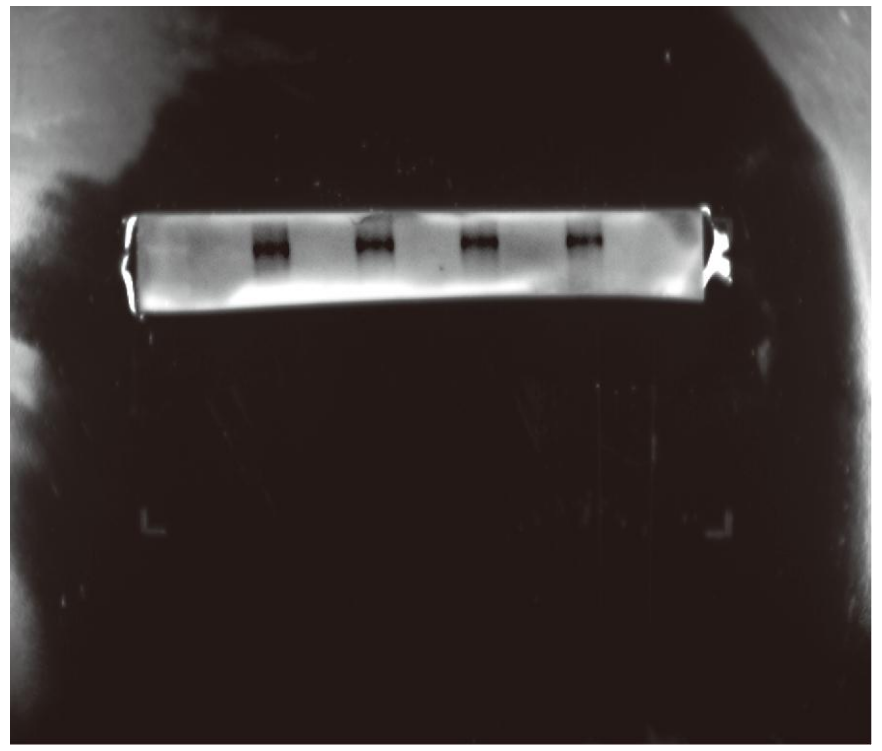

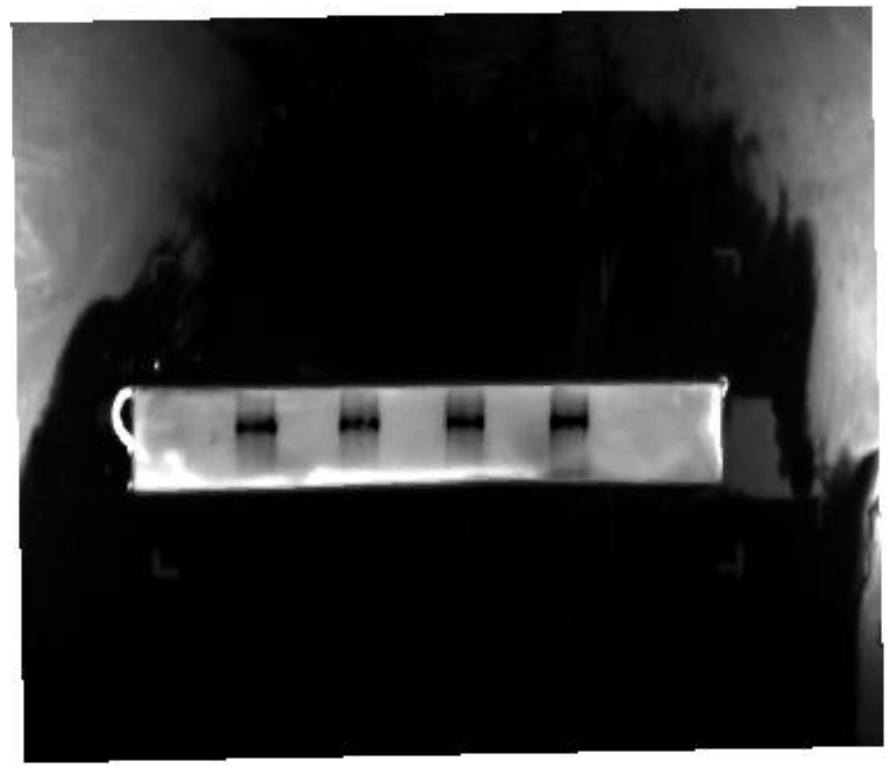

Flag

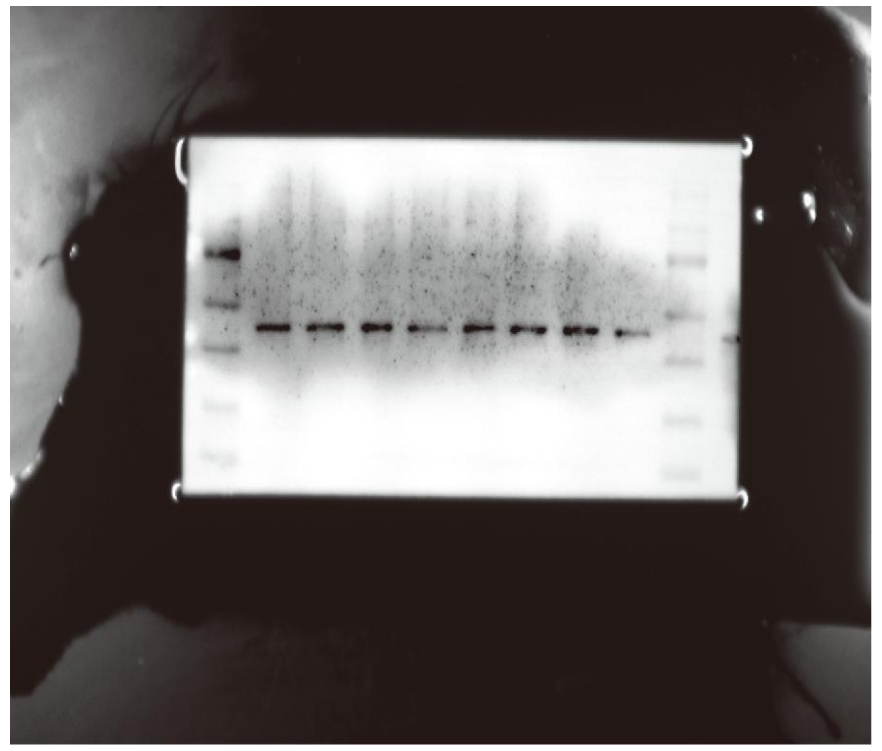

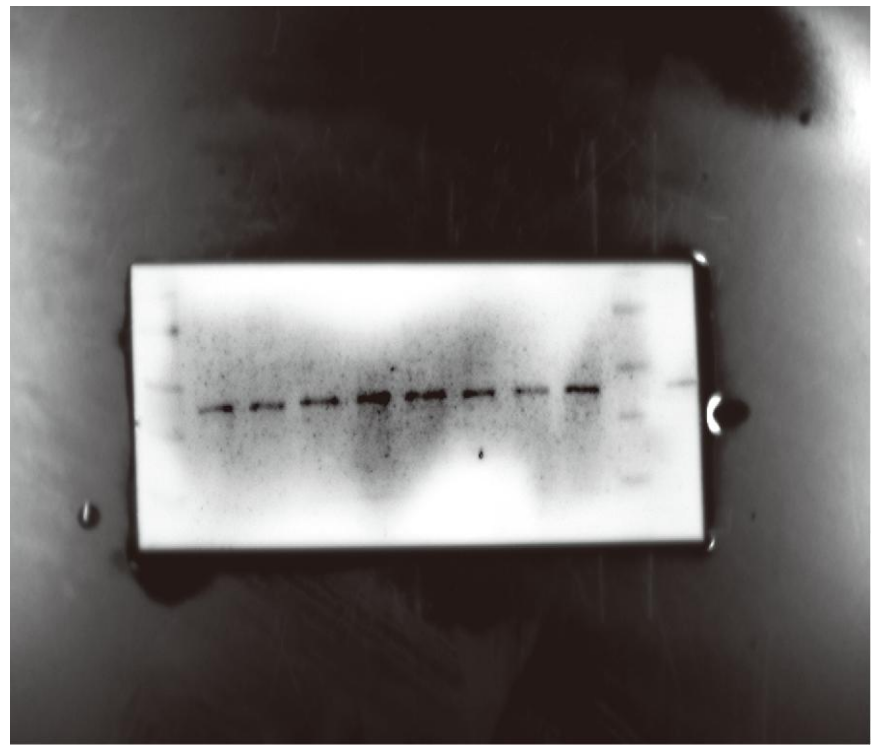

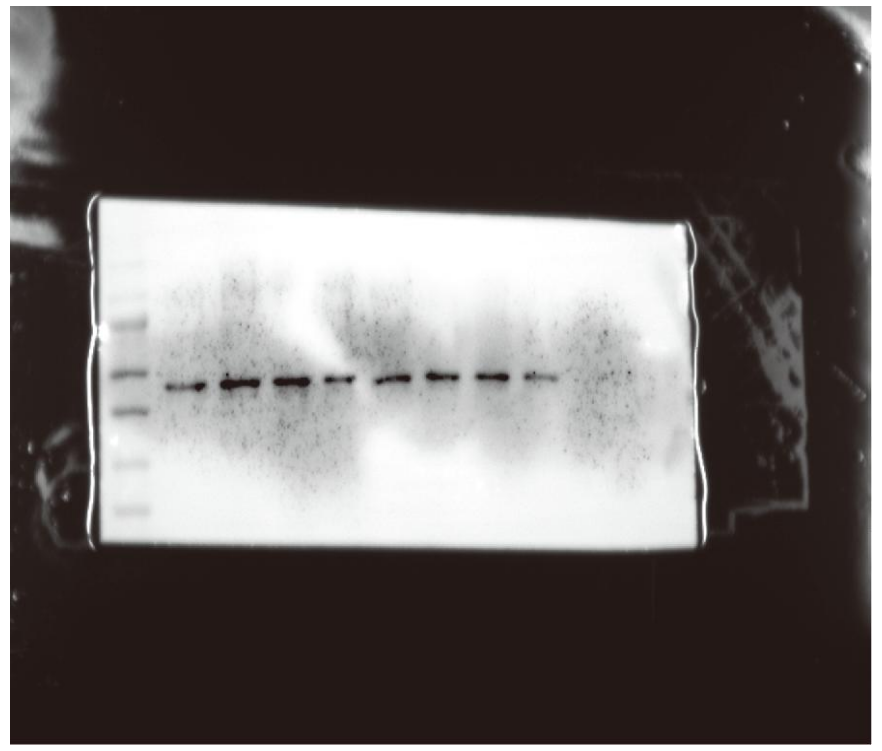

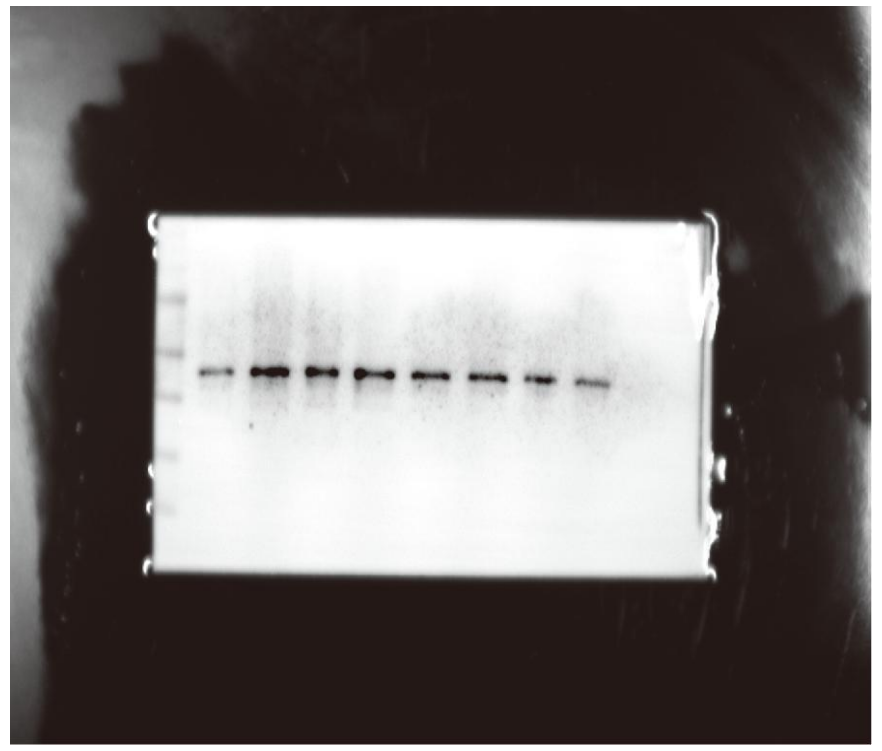

His

Figure 6B

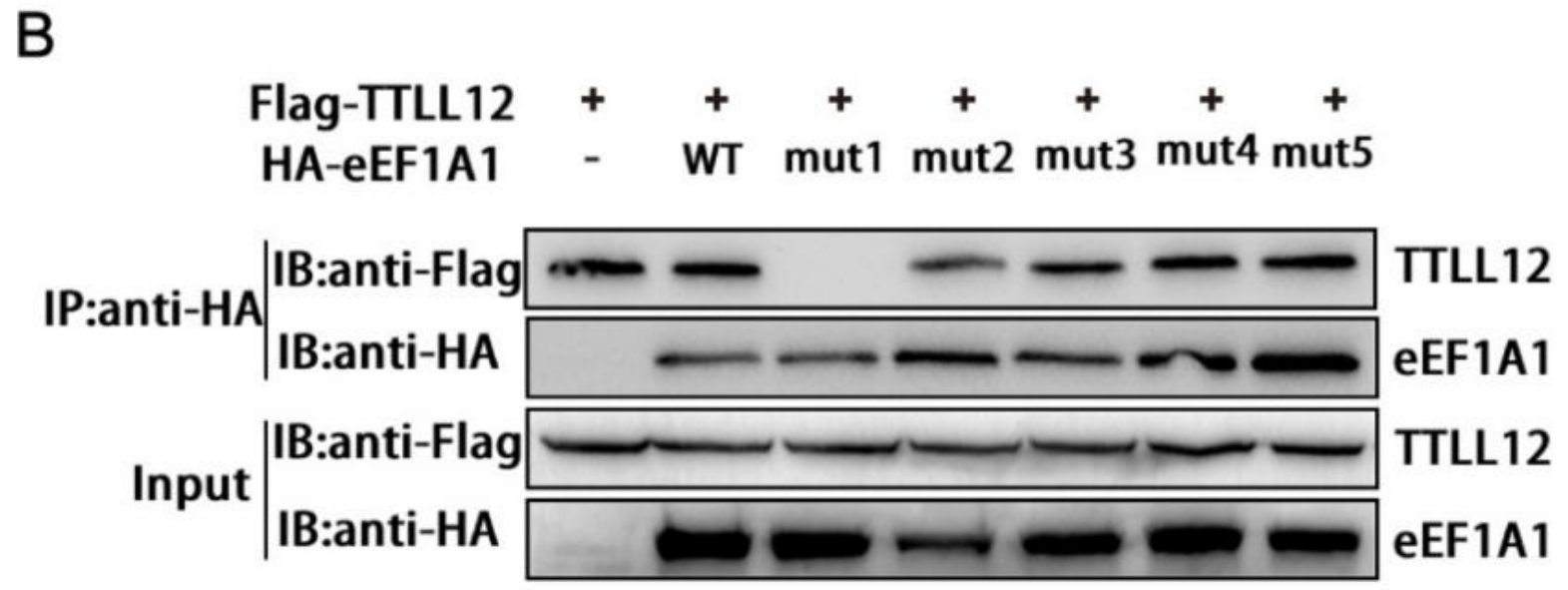

Flag-TTLL12

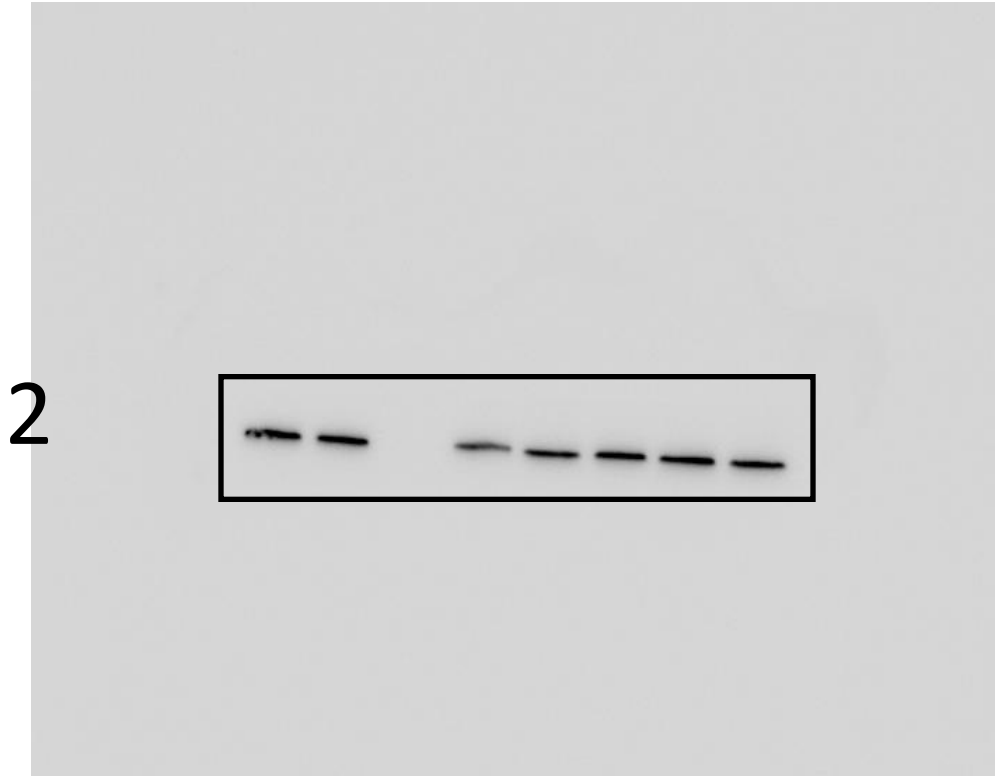

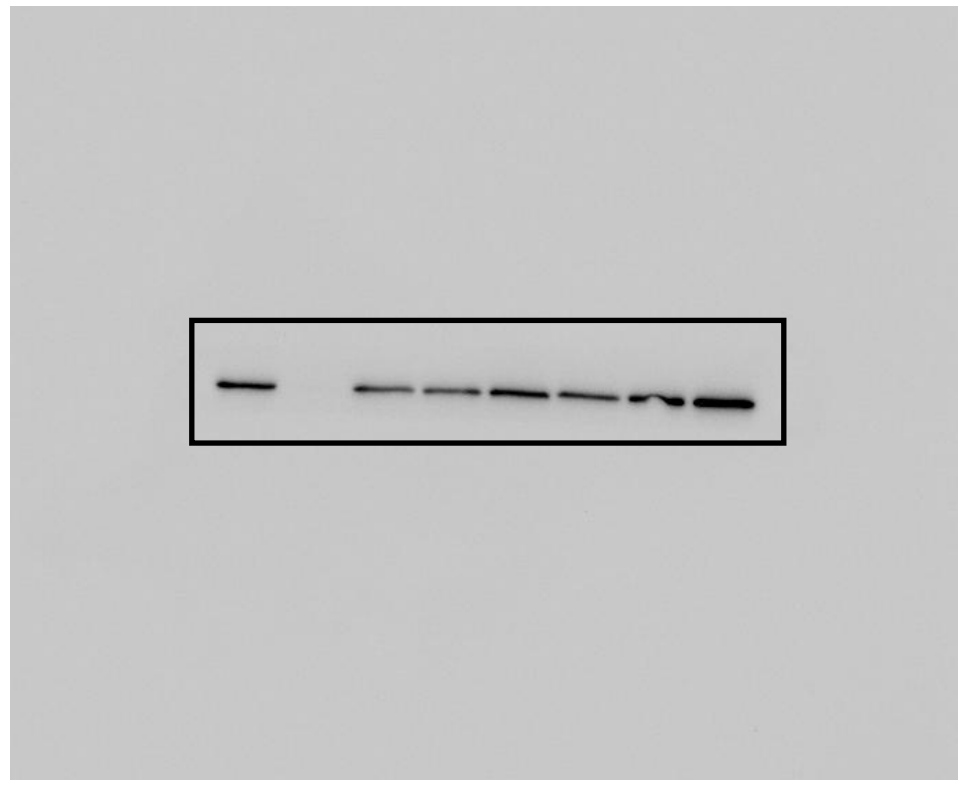

Flag-TTLL12

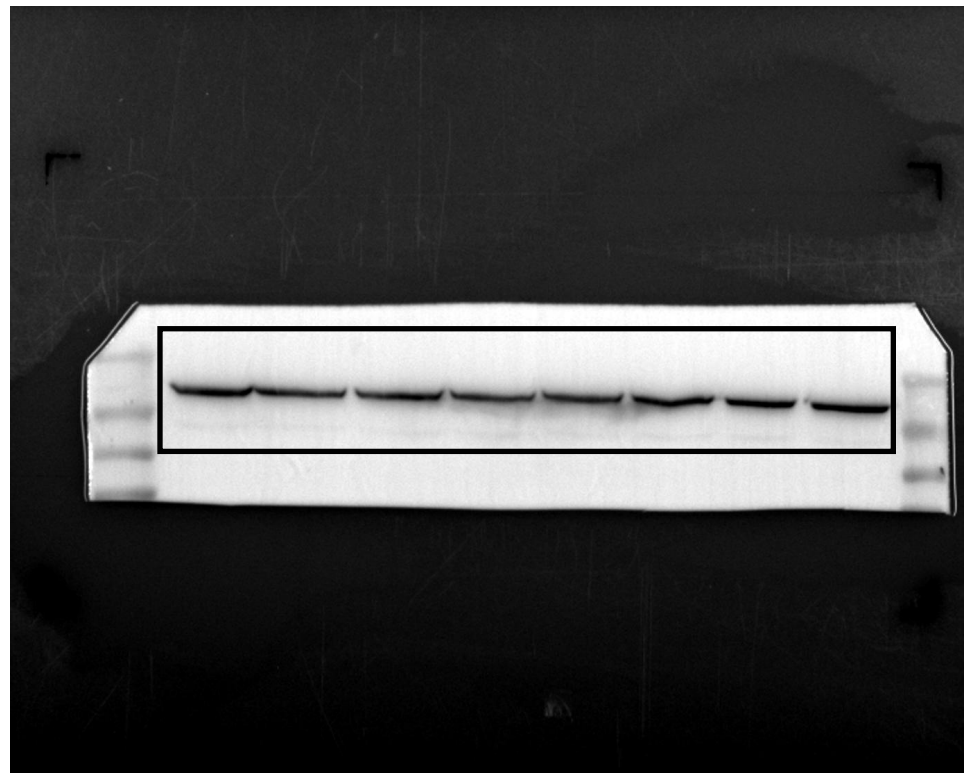

Figure 6C

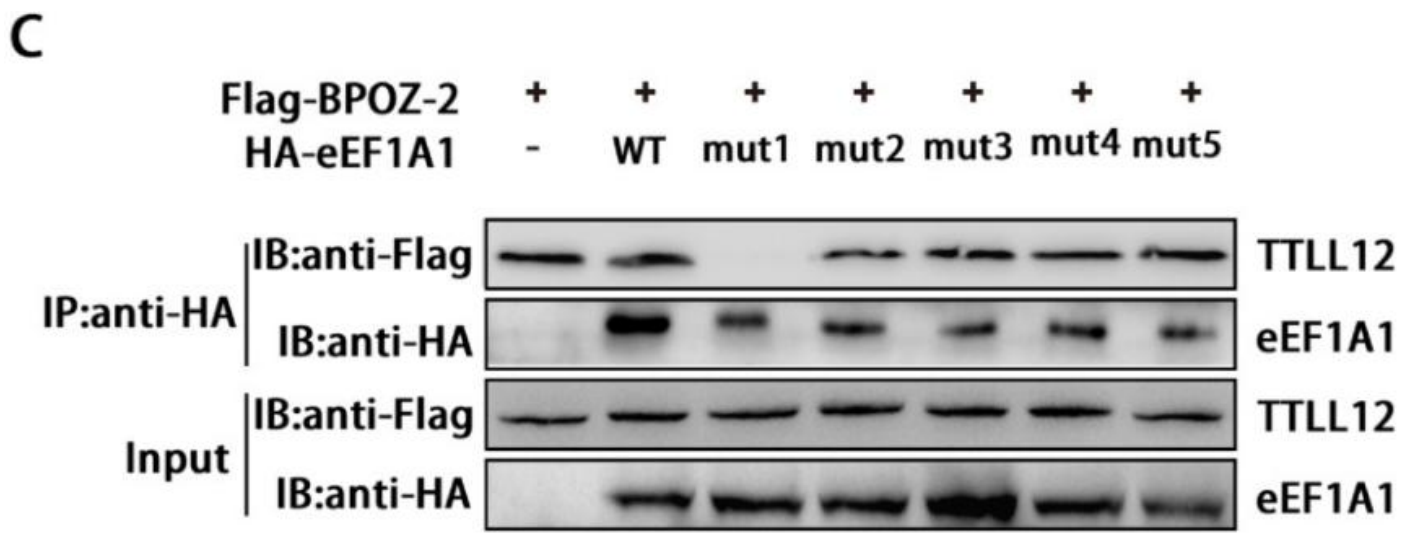

Flag-BPOZ-2

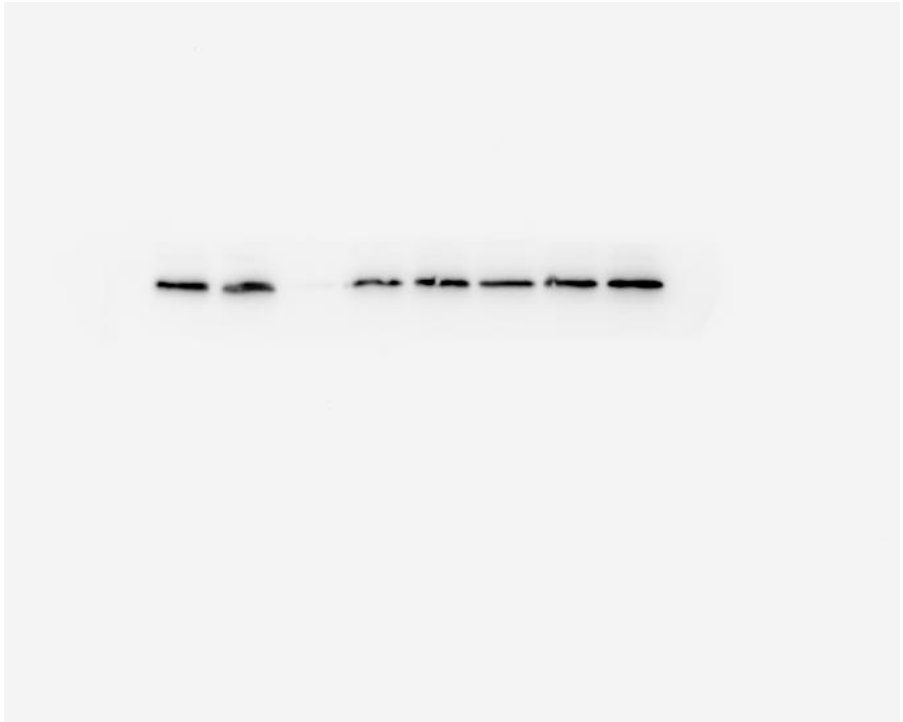

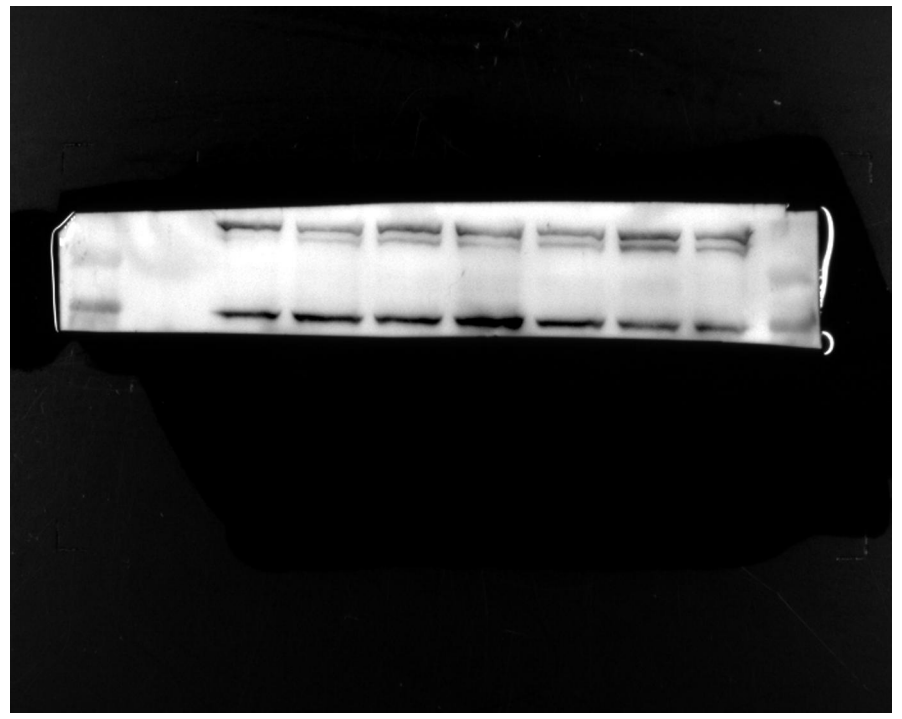

HA-eEF1A1

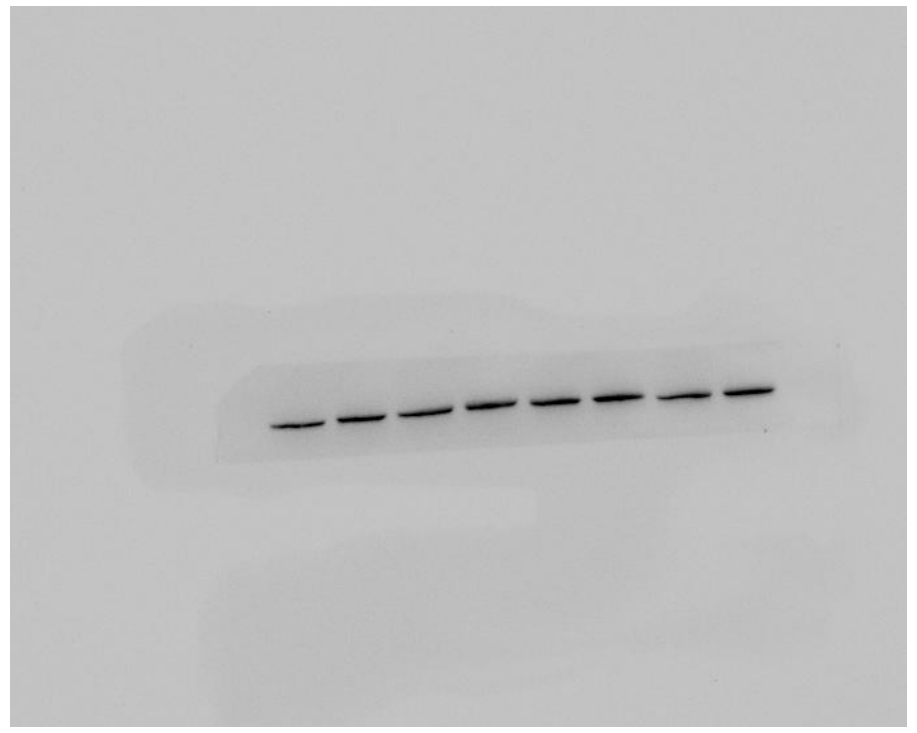

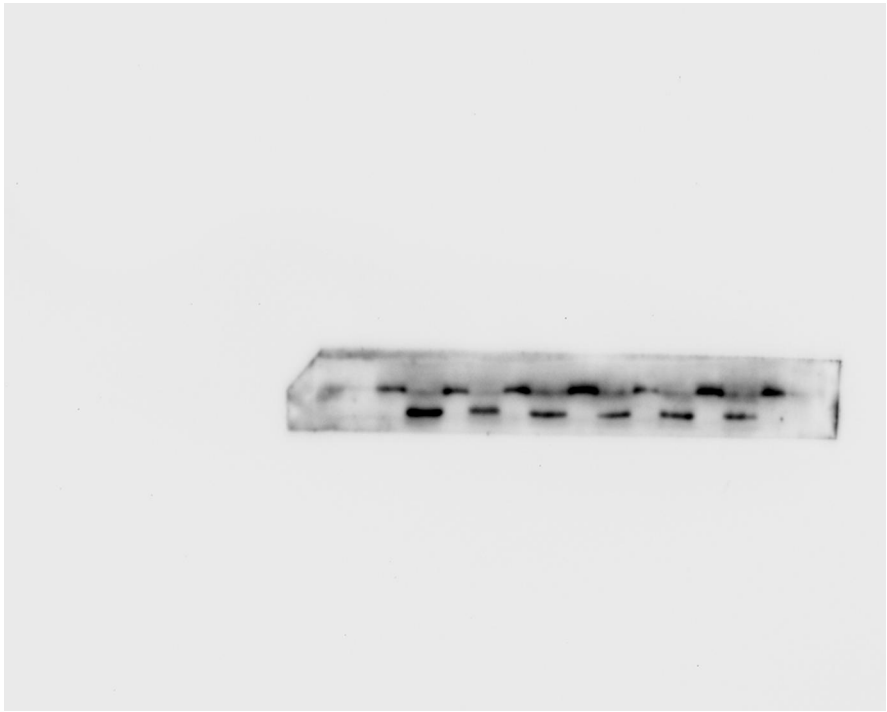

Figure 6D

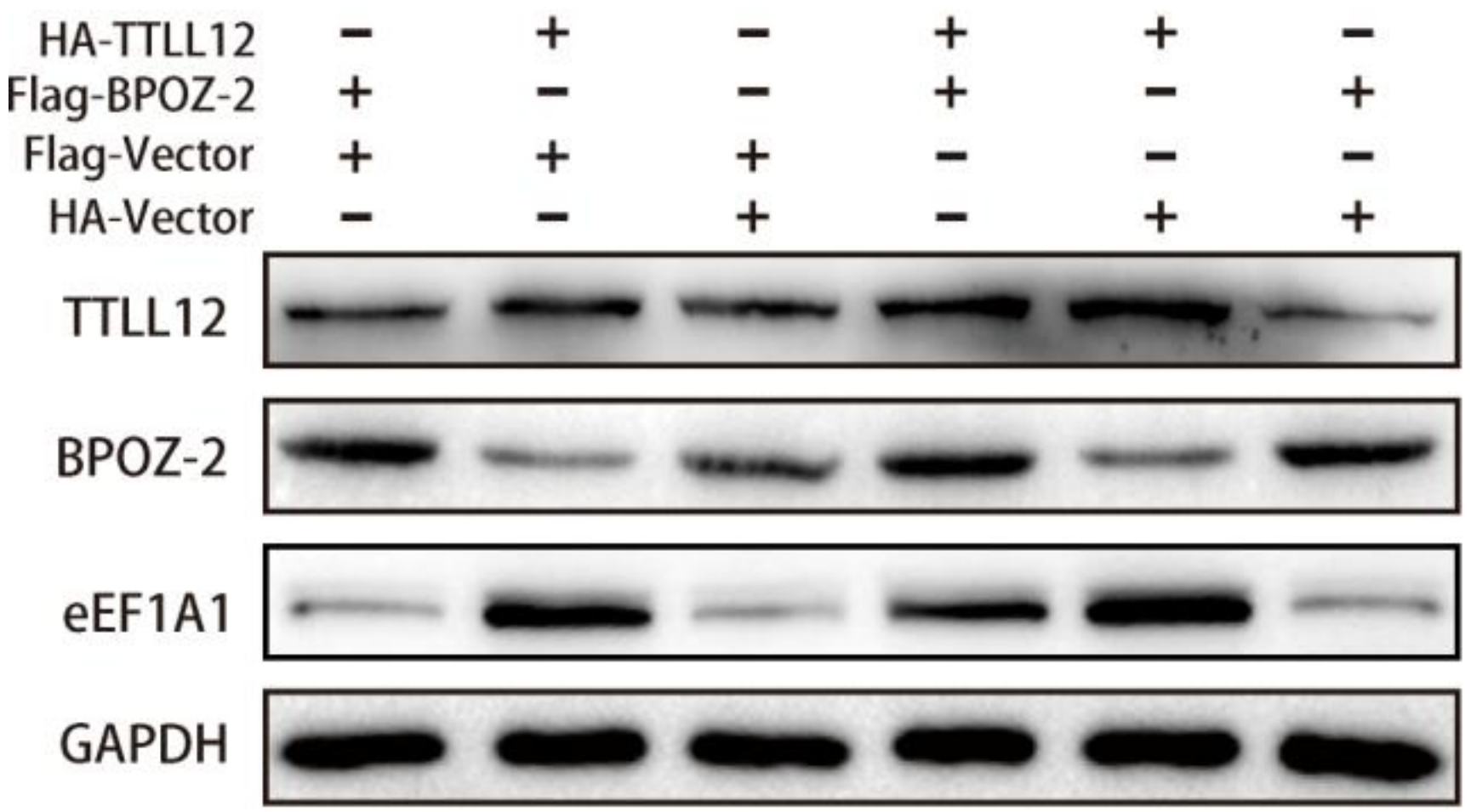

TTLL12

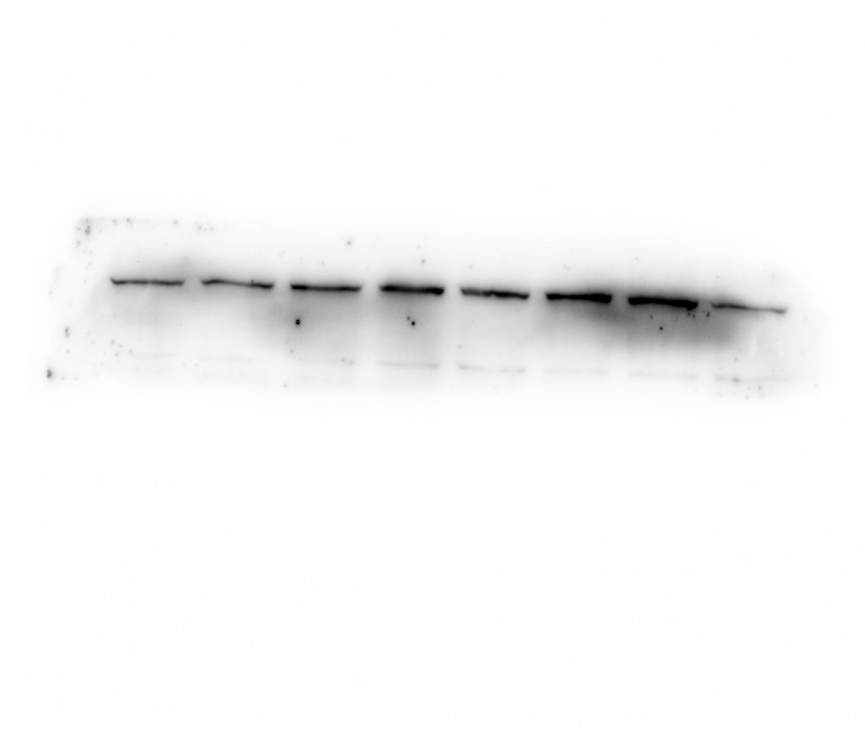

eEF1A1

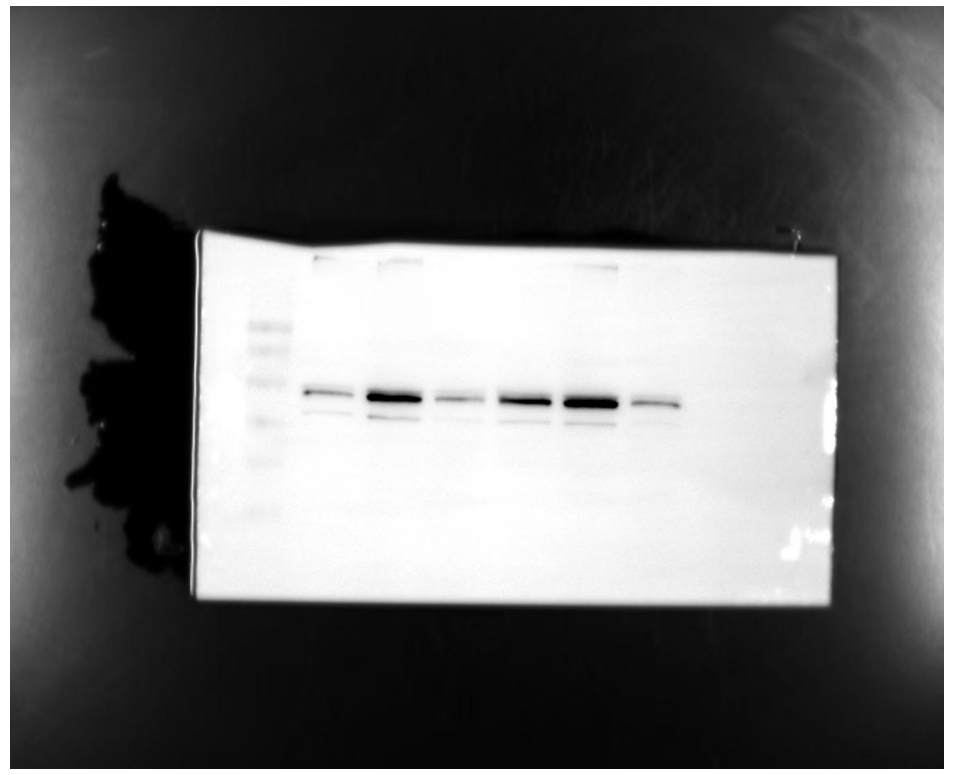

BPOZ-2

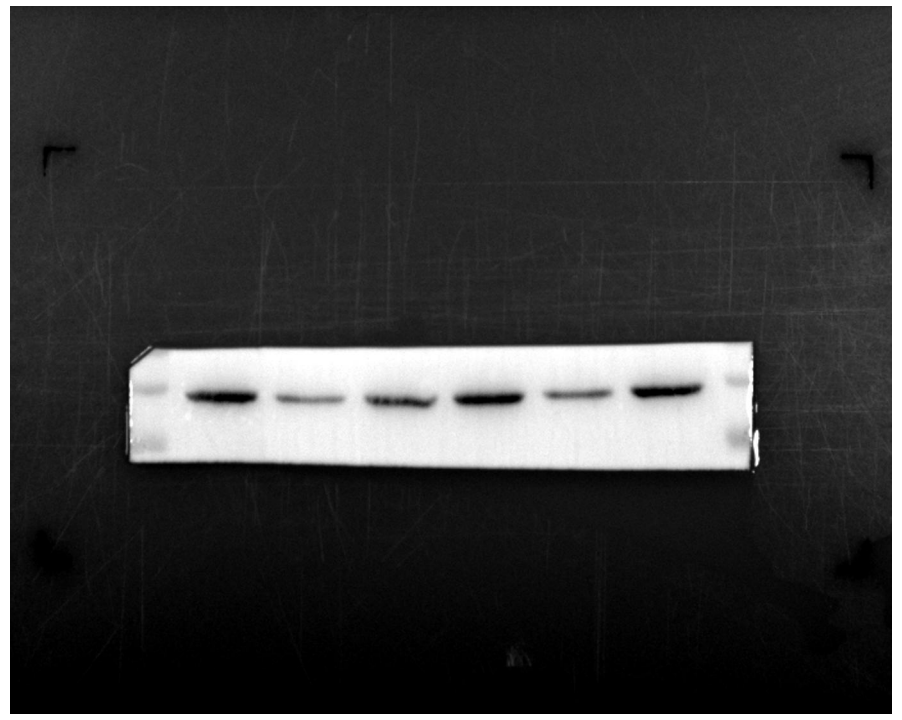

GAPDH

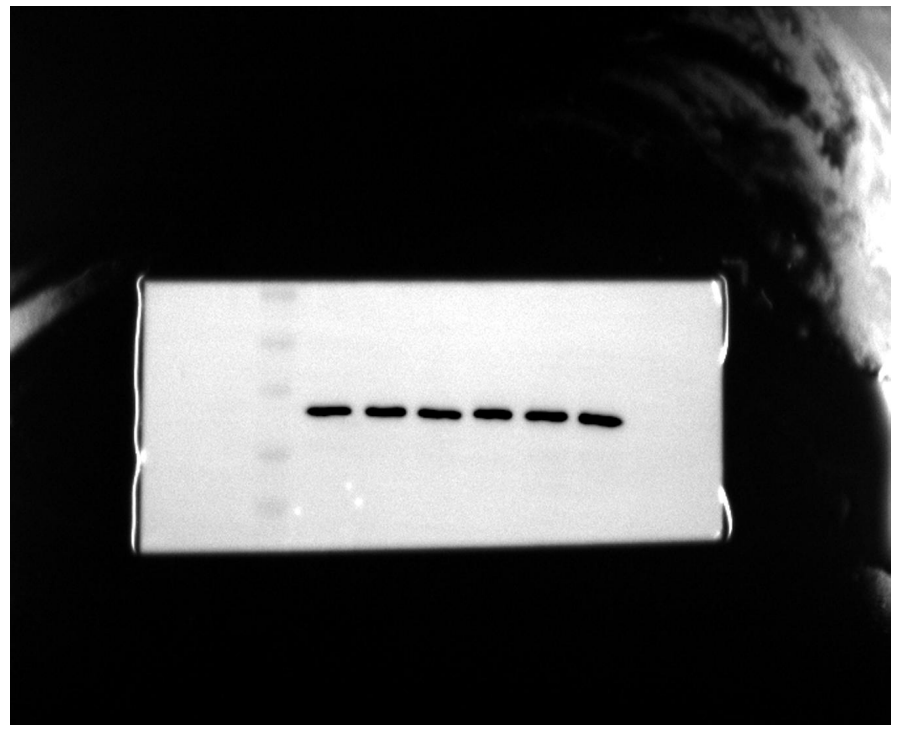

Figure 6E

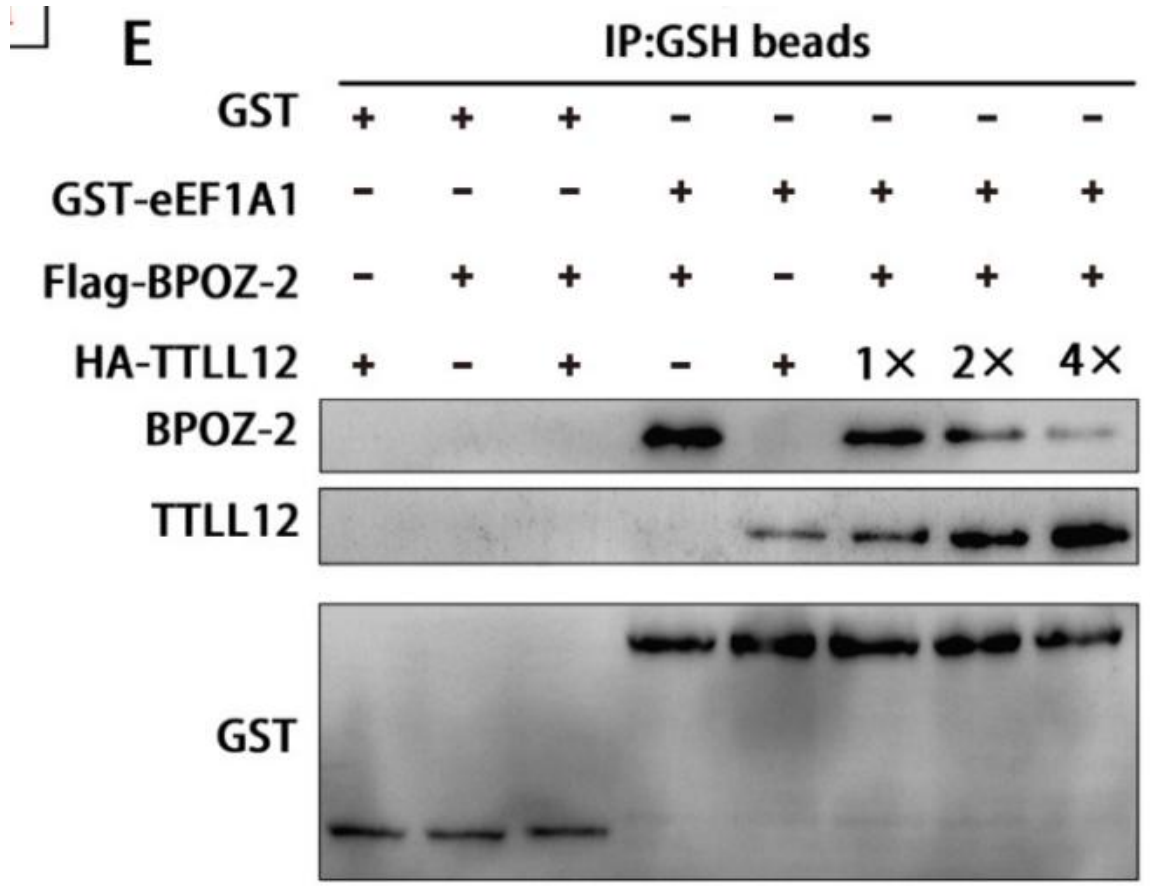

Flag-BPOZ-2

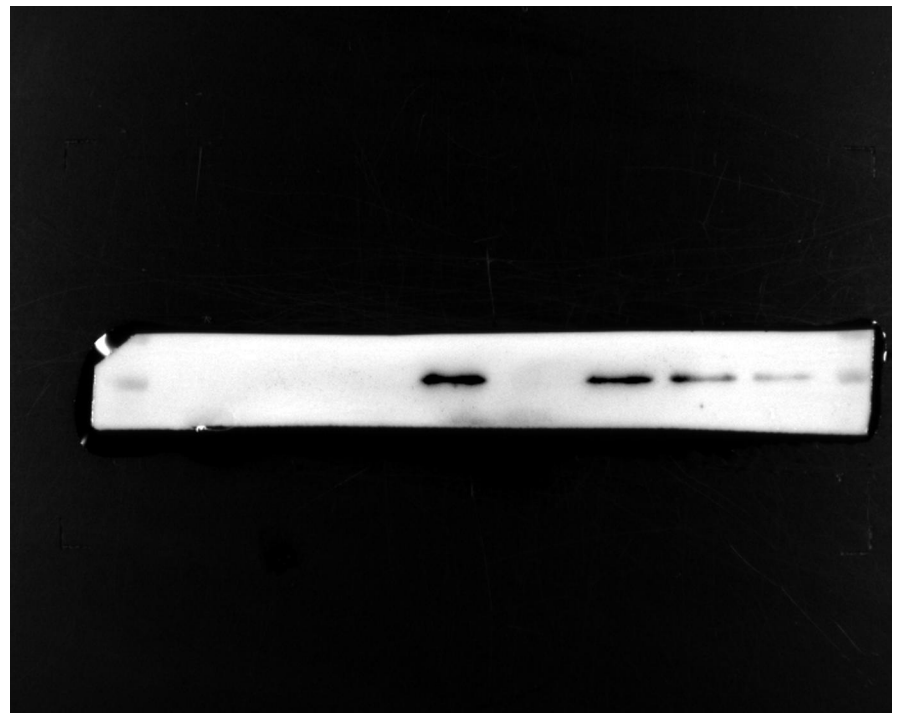

HA-TTLL12

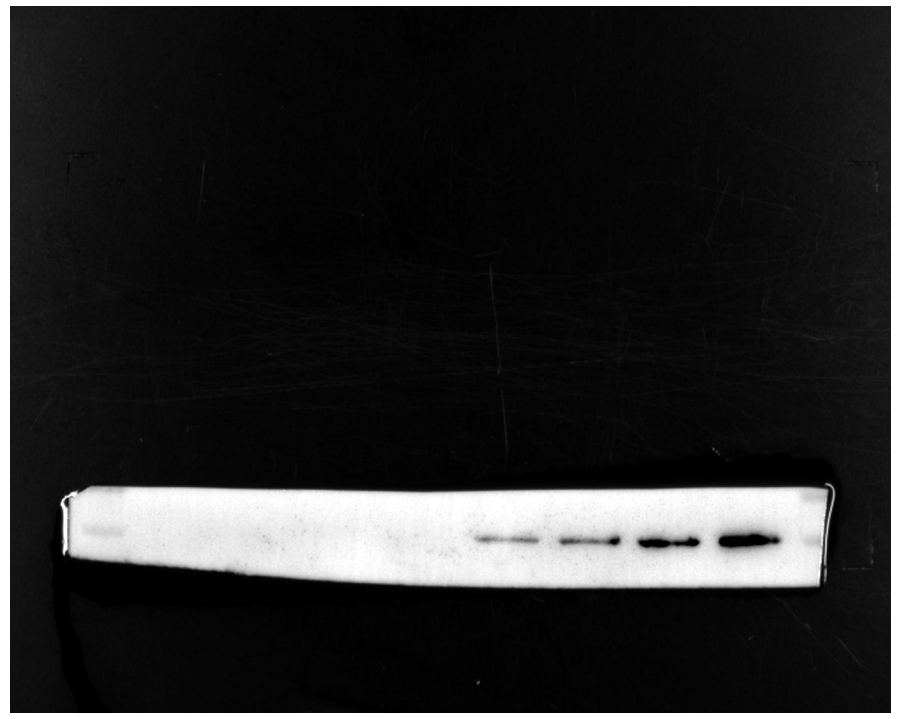

GST

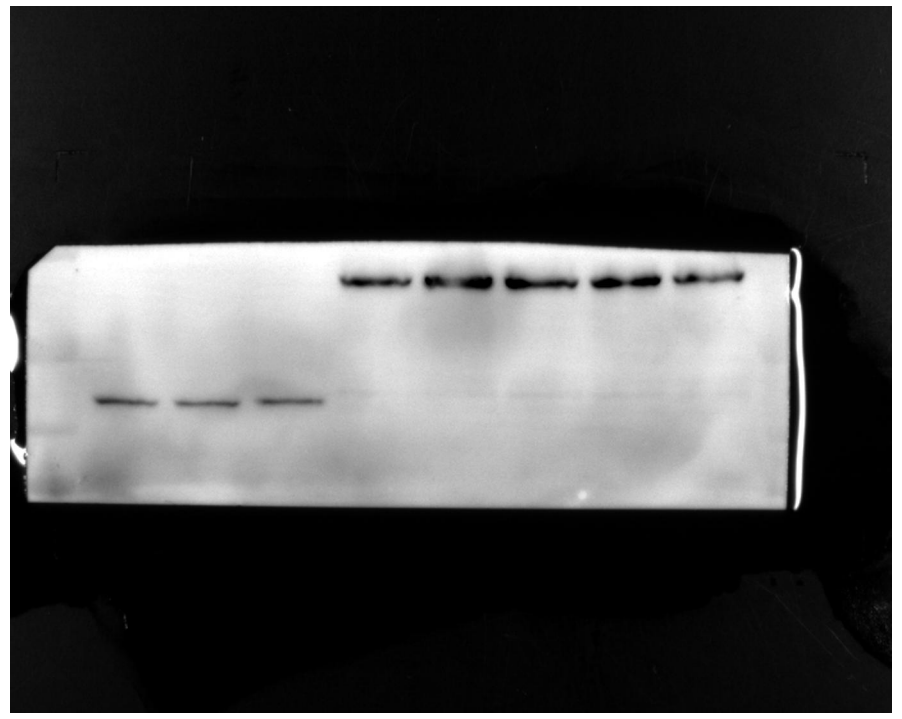

Figure 6F

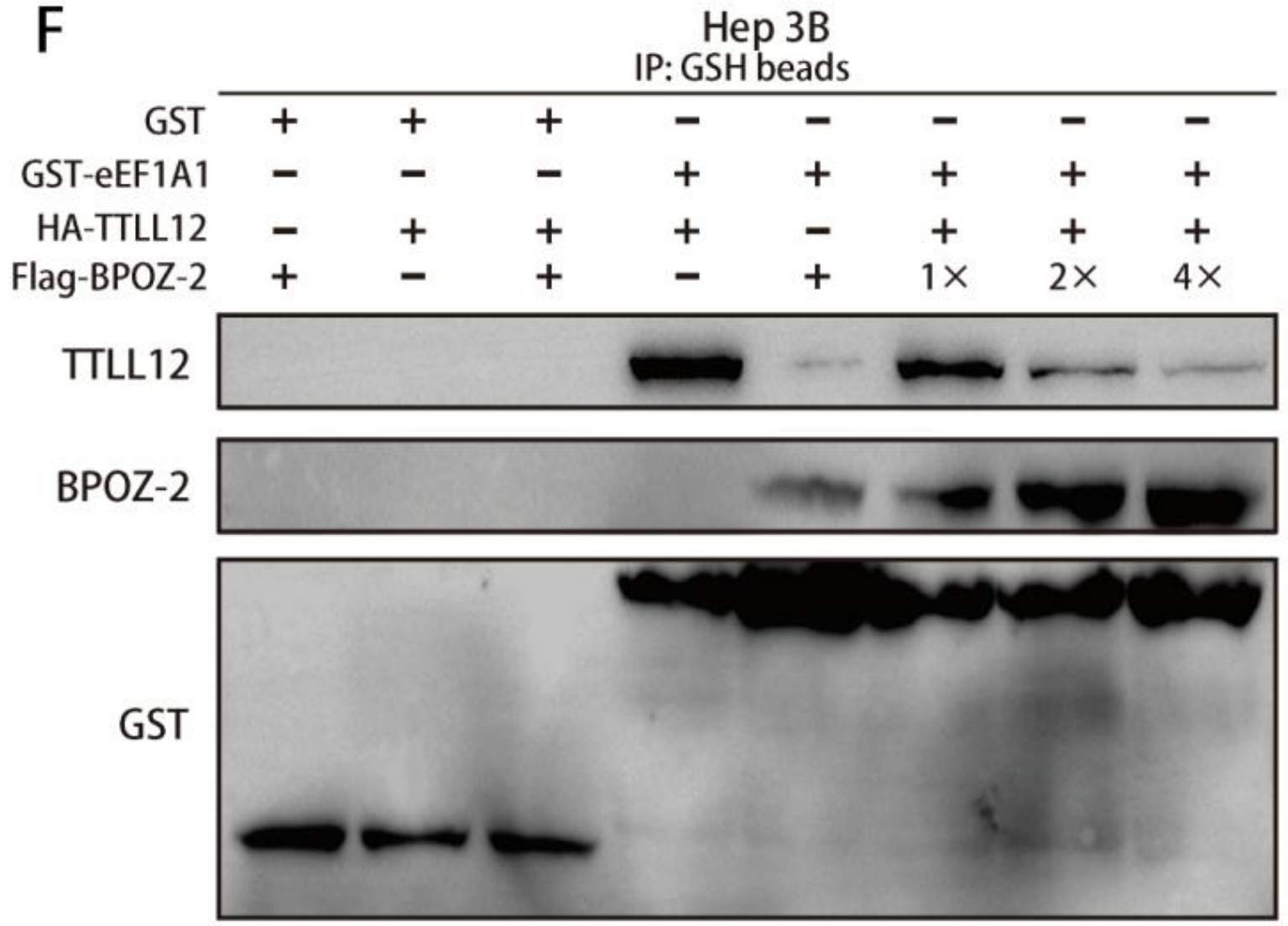

Flag-BPOZ-2

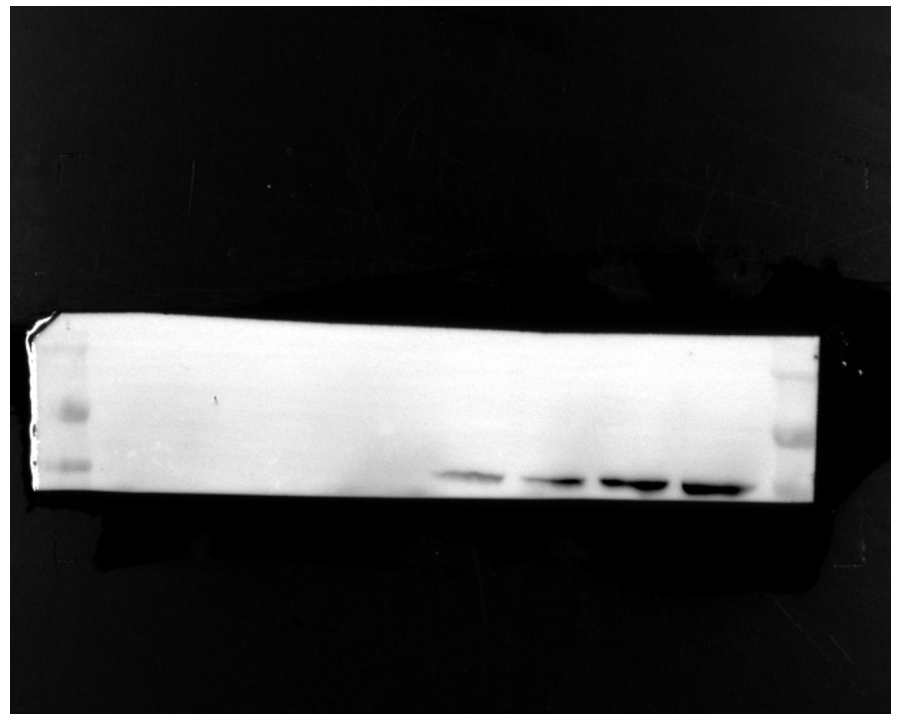

HA-TTLL12

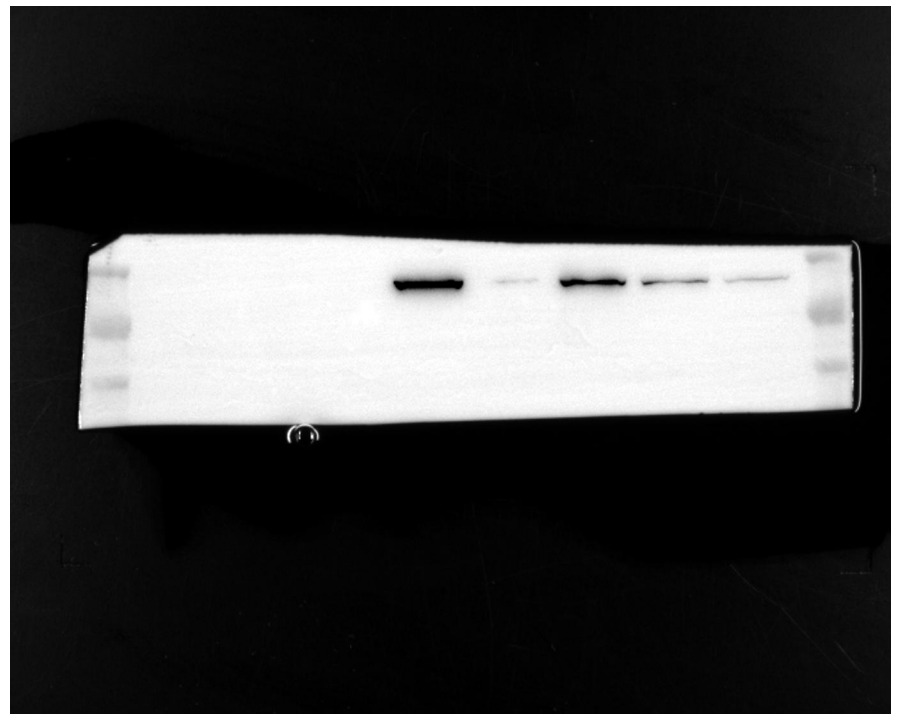

GST

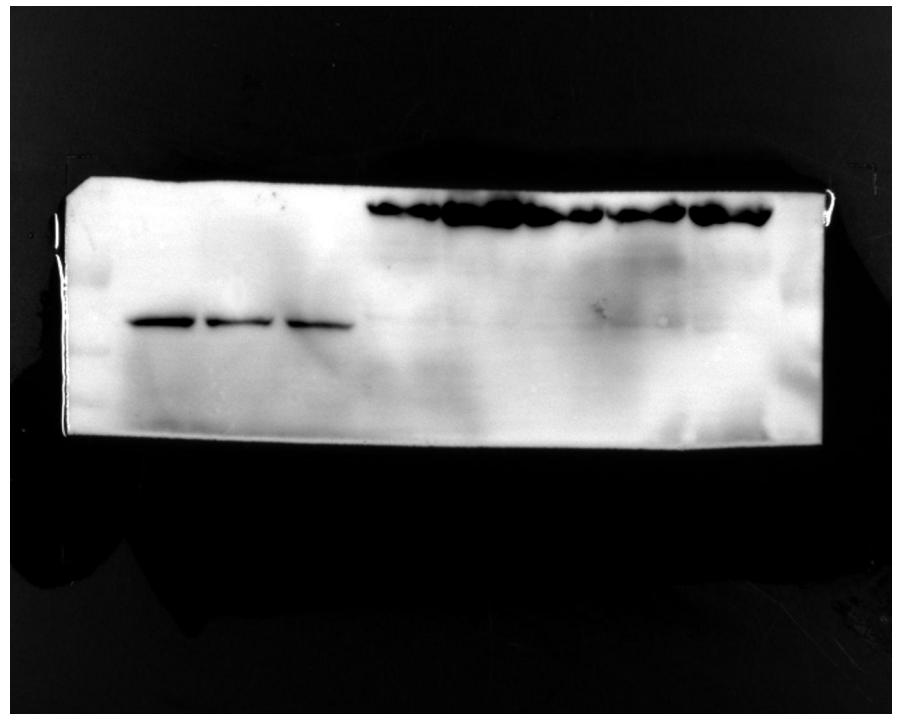

Figure 6G、 6H

eEF1A1 HA-TTLL12 GAPDH

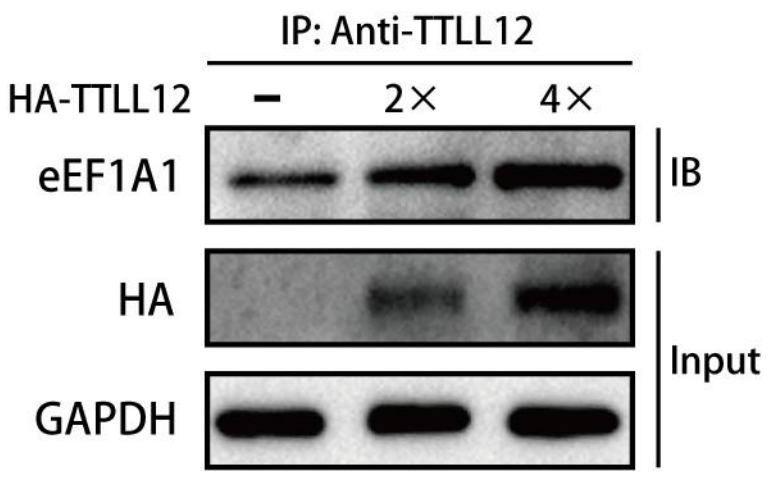

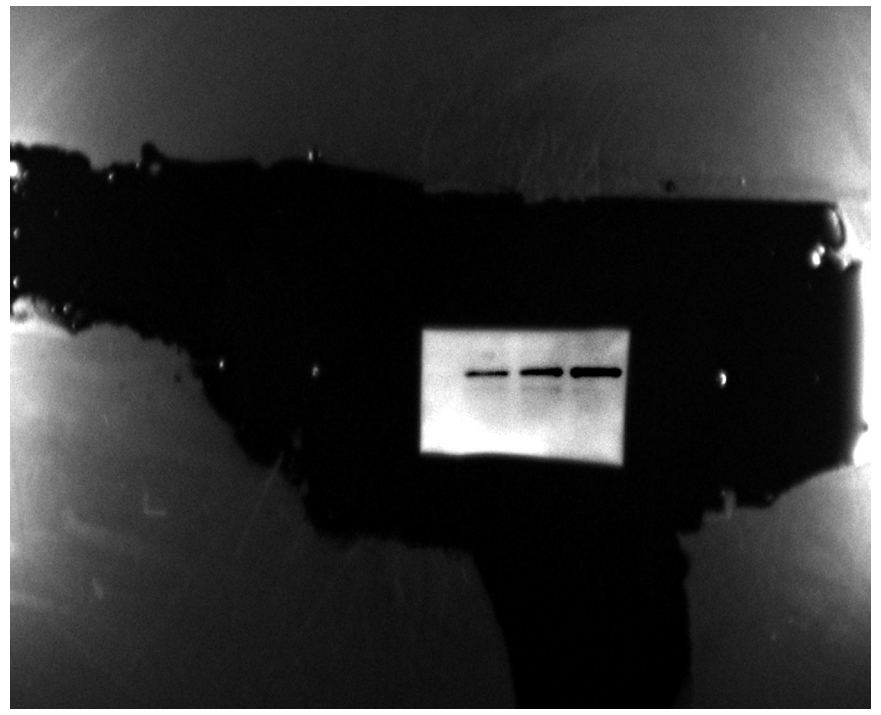

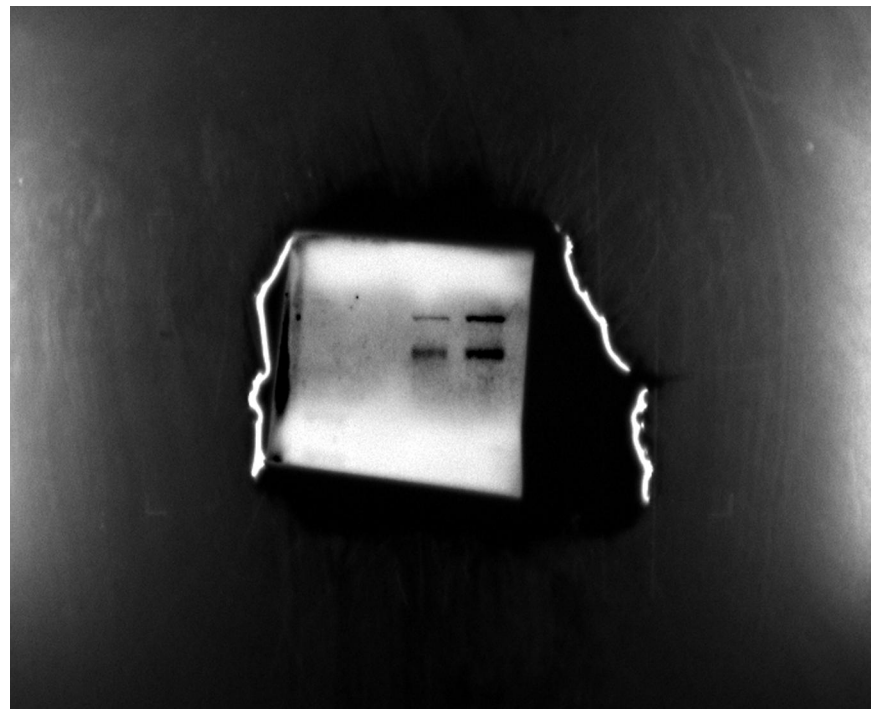

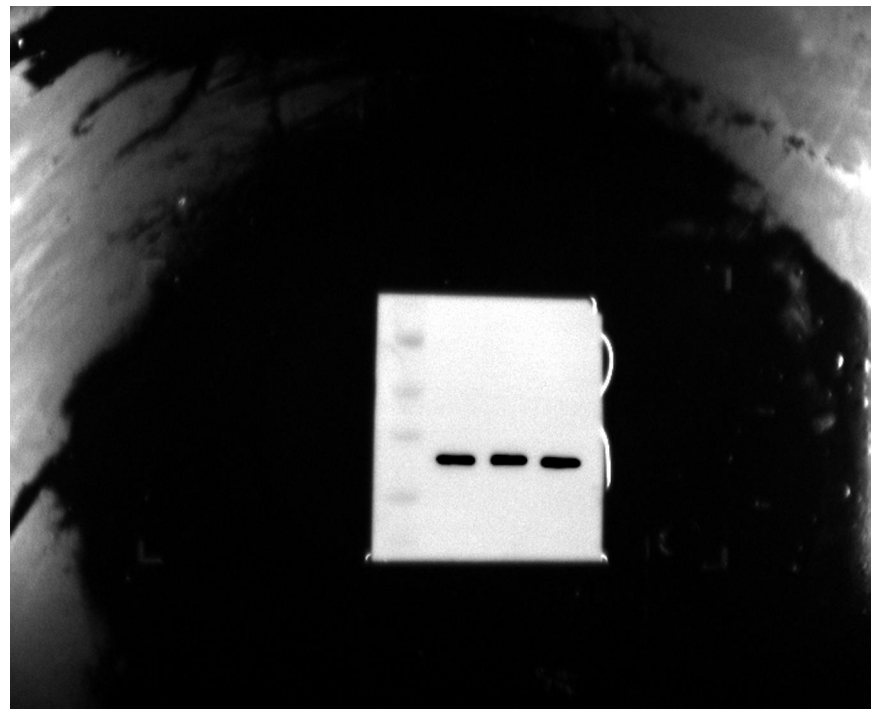

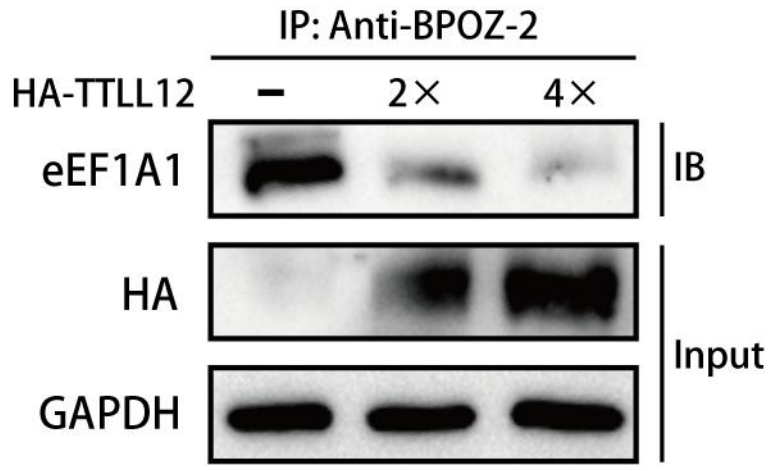

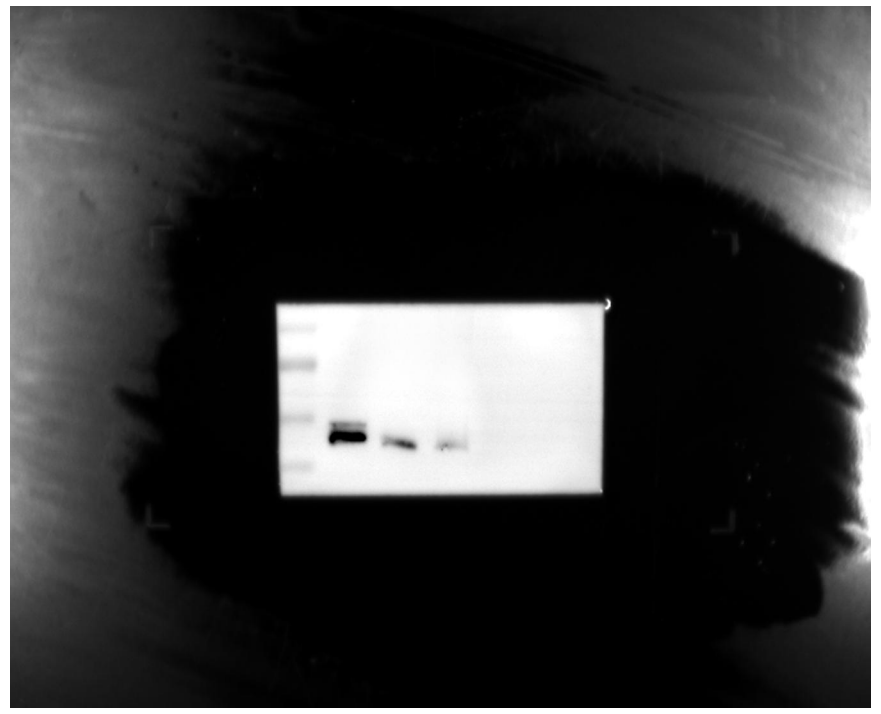

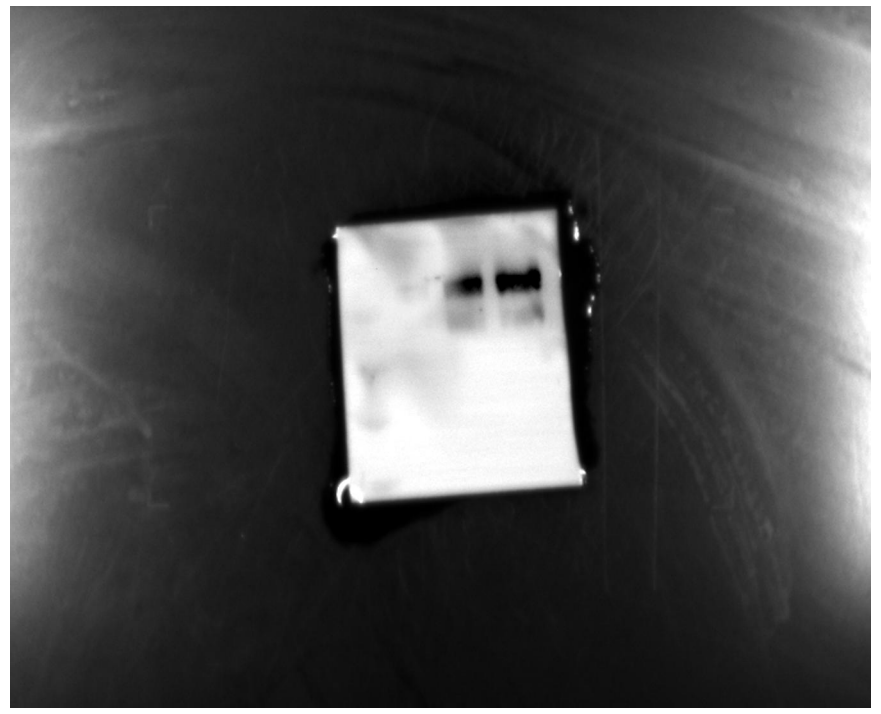

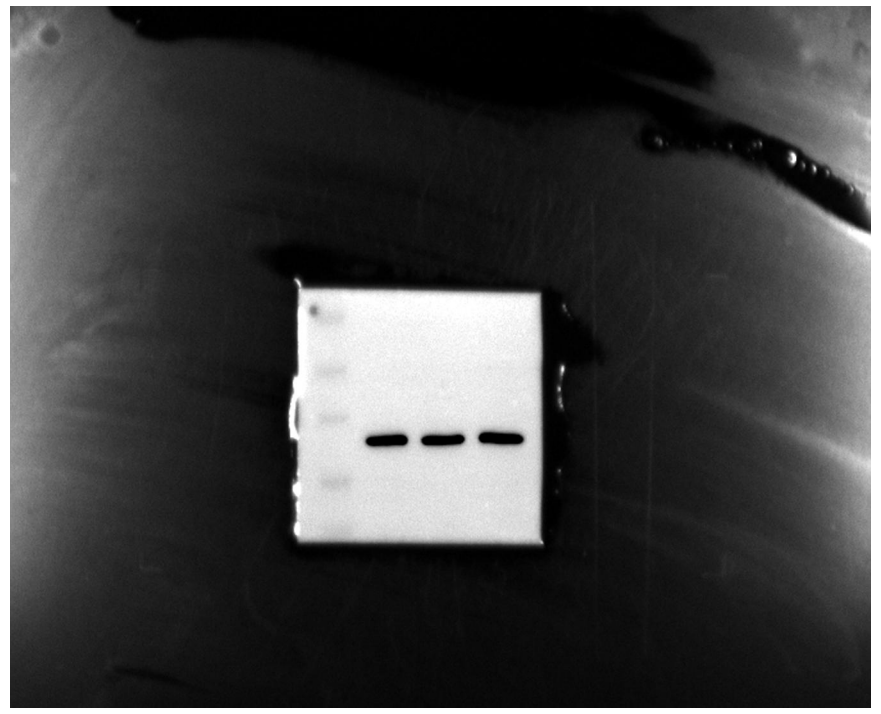

Figure 7B

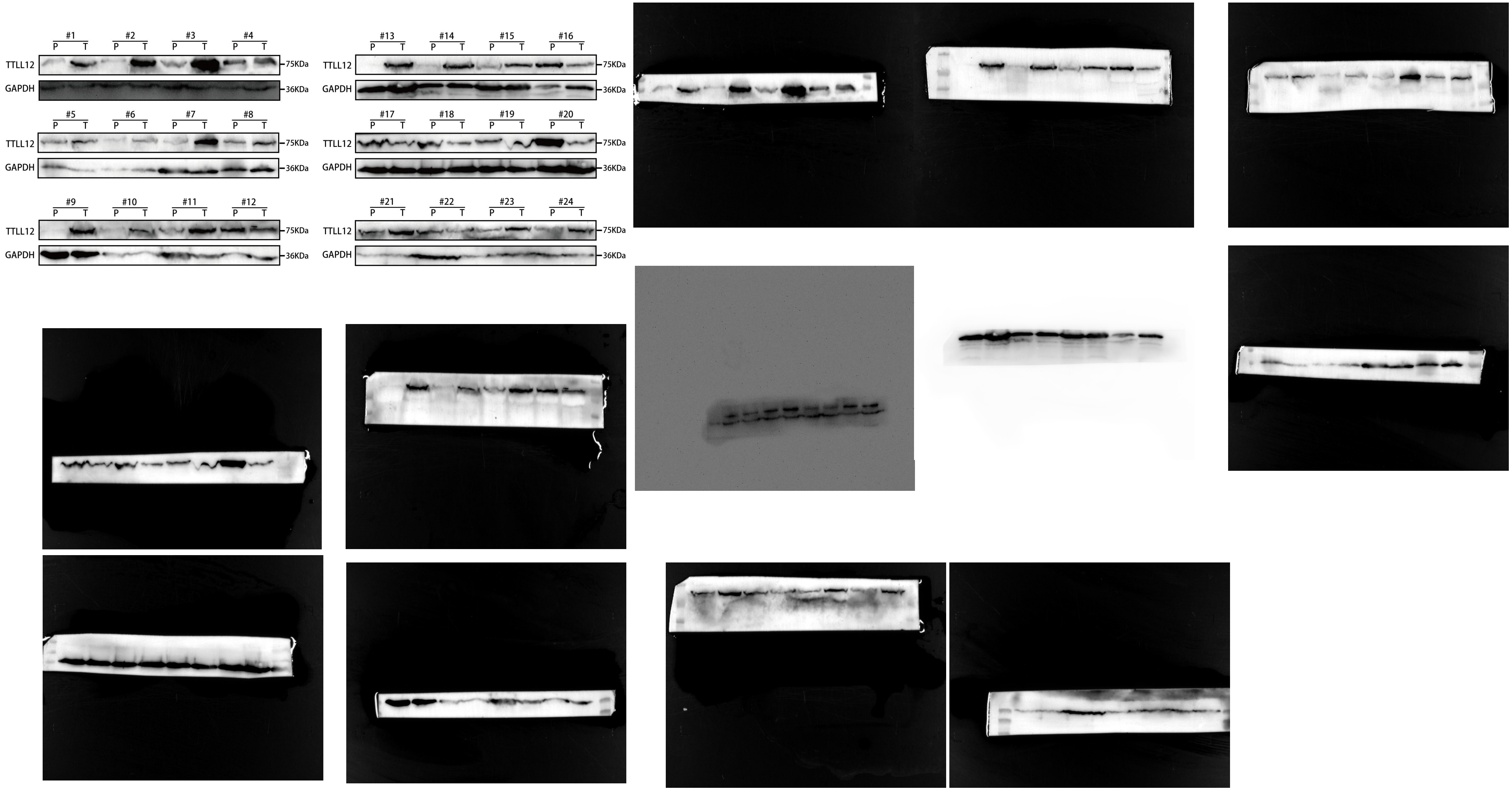
#1 #2 #3 #4
P T P T P T P T
TTLL12
75KDa
GAPDH
36KDa
#5 #6 #7 #8
P T P T P T P T
TTLL12
75KDa
GAPDH
36KDa
#9 #10 #11 #12
P T P T P T P T
TTLL12
75KDa
GAPDH
36KDa
#13 #14 #15 #16
P T P T P T P T
TTLL12
75KDa
GAPDH
36KDa
#17 #18 #19 #20
P T P T P T P T
TTLL12
75KDa
GAPDH
36KDa
#21 #22 #23 #24
P T P T P T P T
TTLL12
75KDa
GAPDH
36KDa
