# Supplementary material for: TTLL12 counteracts BPOZ-2 to stabilize eEF1A1 and promote hepatocarcinogenesis
Source: Cell Death Dis. 2026 Apr 21;17(1):837. doi: 10.1038/s41419-026-08694-1 (PMC13616918; doi:10.1038/s41419-026-08694-1)
Supplement: Supplementary file 2 [file 41419_2026_8694_MOESM1_ESM.docx]

**Table S1.** Primer sequences and shRNA oligonucleotides information.

**Table S2.** List of TTLL12-interacting proteins identified by mass spectrometry.

**Table S3.** Correlation analysis between TTLL12 expression and clinical characteristics in HCC patients.

**Figure S1.** Interaction between TTLL12 and eEF1A1.
**(A)** STRING network showing predicted interactions between TTLL12 and related proteins, including eEF1A1.
**(B)** Silver-stained SDS-PAGE gel of Co-IP results. TTLL12 was immunoprecipitated (IP) from cell lysates, and associated proteins were visualized. TTLL12 and eEF1A1 are indicated by red and blue arrows, respectively.

**Figure S2.** Relative expression of eEF1A1 in Huh7 and Hep3b cells following TTLL12 knockdown.

**Figure S3.** TTLL12 knockdown inhibits tumor growth in xenograft mouse model.

**Figure S4.** Hierarchical clustering heatmap analysis of differentially expressed genes (DEGs) in TTLL12 overexpression and control groups.

**Figure S5.** TTLL12 expression in normal and primary HCC tissues from TCGA database.
