# Supplementary material for: TTLL12 counteracts BPOZ-2 to stabilize eEF1A1 and promote hepatocarcinogenesis
Source: Cell Death Dis. 2026 Apr 21;17(1):837. doi: 10.1038/s41419-026-08694-1 (PMC13616918; doi:10.1038/s41419-026-08694-1)
Supplement: Supplementary file 3 [file 41419_2026_8694_MOESM4_ESM.docx]

| Table S3. Correlation analysis between TTLL12 expression and clinical characteristics in HCC patients. | | | | |
| --- | --- | --- | --- | --- |
| **Variables** | **N** | **TTLL12 expression** | | **P** |
|  |  | **Low** | **High** |  |
| **Age** |  |  |  | 0.787 |
| ≤55 | 51 (56.7%) | 26 (55.3%) | 25 (58.1%) |  |
| >55 | 39 (43.3%) | 21 (44.7%) | 18 (41.9%) |  |
| **Gender** |  |  |  |  |
| Female | 10 (11.1%) | 5 (10.6%) | 5 (11.6%) |  |
| Male | 80 (88.9%) | 42 (89.4%) | 38 (88.4%) | 0.881 |
| **Tumor differentiation** |  |  |  | 0.099 |
| High | 62 (68.9%) | 36 (76.6%) | 26 (60.5%) |  |
| Low | 28 (31.1%) | 11 (23.4%) | 17 (39.5%) |  |
| **TNM stage** |  |  |  | 0.012 |
| 1-2 | 48 (53.3%) | 31 (66.0%) | 17 (39.5%) |  |
| 3-4 | 42 (46.7%) | 16 (34.0%) | 26 (60.5%) |  |
