# Supplementary material for: TTLL12 counteracts BPOZ-2 to stabilize eEF1A1 and promote hepatocarcinogenesis
Source: Cell Death Dis. 2026 Apr 21;17(1):837. doi: 10.1038/s41419-026-08694-1 (PMC13616918; doi:10.1038/s41419-026-08694-1)
Supplement: Supplementary file 4 [file 41419_2026_8694_MOESM2_ESM.doc]

Table S1. Primer sequences and shRNA oligonucleotides information.

|  | Sequence |
| --- | --- |
| eEF1A1 | Forward primer: 5′-TGTCGTCATTGGACACGTAGA-3′  Reverse primer: 5′-ACGCTCAGCTTTCAG TTTATCC-3′ |
| GAPDH | Forward primer: 5′-GGGAGCCAAAAGGGTCAT-3′  Reverse primer: 5′-GAGTCCTTCCACGATACCAA-3′ |
| sh-TTLL12-1 | 5′-CCUUCUUCAACGACGUCUU-3′ |
| sh-TTLL12-2 | 5′-GGUAUAUCAUGGACGAGUU-3′ |
| sh-TTLL12-3 | 5′-GCCACAUCUUCAAGGUCUA-3′ |
| sh-NC | 5′-TTCTCCGAACGTGTCACGT-3′ |
